# An anatomical atlas of *Drosophila melanogaster—*the wild-type

**DOI:** 10.1093/genetics/iyae129

**Published:** 2024-09-06

**Authors:** Kai J Jürgens, Maik Drechsler, Achim Paululat

**Affiliations:** Department of Zoology and Developmental Biology, Osnabrück University, Osnabrück 49076, Germany; Department of Zoology and Developmental Biology, Osnabrück University, Osnabrück 49076, Germany; Center of Cellular Nanoanalytics (CellNanOS), Osnabrück University, Osnabrück 49076, Germany; Department of Zoology and Developmental Biology, Osnabrück University, Osnabrück 49076, Germany; Center of Cellular Nanoanalytics (CellNanOS), Osnabrück University, Osnabrück 49076, Germany

**Keywords:** scanning electron microscopy, flybook, anatomy, morphology, external markers

## Abstract

Scanning electron microscopy is the method of choice to visualize the surface structures of animals, fungi, plants, or inorganic objects at the highest resolution and often with impressive appeal. Numerous scanning electron microscope (SEM) images exist of *Drosophila melanogaster*, one of the most important model organisms in genetics and developmental biology, which have been taken partly for esthetics and often to solve scientific questions. Our work presents a collection of images comprising many prominent anatomical details of *D. melanogaster* in excellent quality to create a research and teaching resource for all Drosophilists.

## Why we present an SEM anatomy atlas

*Drosophila melanogaster* has been used in biological research for over 100 years. The first known laboratory experiments with *Drosophila* date back to Thomas Hunt Morgan and his scholars at Caltech in California around 1900 and the following years. Thomas Hunt Morgan published 22 books and over 300 scientific articles. The first spontaneous mutation, the *white* mutant, was discovered in his laboratory (Morgan 1910) and is still used today as one of the most popular phenotypic markers. Only a few years later, the *Bar* mutation, which severely affected eye morphology, was used to discover positional effects and cross-over (Sturtevant 1925). Two years later, techniques were found to induce mutants experimentally (Muller 1927). To all readers interested in the early history of genetic research, especially the significance of the work of Thomas Hunt Morgan and his collaborators, we recommend the book “A History of Genetics” by Alfred H. Sturtevant, one of Morgan's first Ph.D. students (Sturtevant 1966).

More than 100 years after the first *Drosophila* experiments by Morgan, more than 50,000 scientific papers have been published in renowned peer-reviewed journals in which *D. melanogaster* is at center stage. Countless photographs of wild-type and mutant animals have been published, and basic anatomical details have been described in detail in several pioneering textbooks, such as the famous “*Biology of Drosophila*” (Demerec 1950)—known in fly labs worldwide simply as the “*Demerec.*” To date, almost every external feature of *Drosophila* has been illustrated in an article or a book. Nevertheless, for our daily work in a *Drosophila* laboratory and the education of our young students, an atlas with excellent quality illustrations of the essential external features is beneficial. This gap has already been filled perfectly by Chyb and Gompel with their wonderful book “*Atlas of Drosophila Anatomy*” (Chyb and Gompel 2013). This book's light microscopic images of wild-type and mutant flies are brilliant and indispensable for our daily use in fly work. However, when analyzing new mutants, one often desires to view and study anatomical details at a much higher resolution, which can only be achieved by scanning electron microscopy. A recent reference work compiling SEM (scanning electron microscope) photographs of the major anatomical features of wild-type larvae and adult flies and selected mutants in a single atlas has been lacking since Hodgkin and Bryant published a collection of SEM images of the adult fly (Hodgkin and Bryant 1978). Therefore, we aimed to present the external morphology of flies in esthetic, particularly challenging photographs at the highest possible resolution.

We hope that readers will appreciate the images published in this article and that they will be helpful in the phenotypic analysis of new mutants and data presentation at scientific conferences or regular laboratory meetings. Therefore, we will make all images available as downloads with and without labels so they can be modified and annotated only for structures relevant to one's research work. Images will be available via Figshare at: https://figshare.com/projects/An_anatomical_atlas_of_Drosophila_melanogaster_–_the_wildtype/218623

We would also like to thank our department's technical assistants, who helped keep the fly stocks healthy and prepared excellent and nutritious food. Our SEM anatomy atlas will be a valuable and helpful tool for teaching and research and will be widely distributed among *Drosophila* research laboratories.

## Materials and methods

To establish a set of reference images of *D. melanogaster*, we exclusively used the genotype *Oregon-R* to acquire all the photos. We aimed to compile an atlas presenting esthetically pleasing and informative images that can be used for teaching and daily laboratory use. Therefore, we have paid particular attention to ensuring that all animals or parts of animals are carefully prepared and oriented in a natural position. The most time-consuming part of this work was not photographing the animals with the SEM but the perfect preparation and alignment of the objects and removing dust particles. Excellent photos were rarely taken on the first attempt. Therefore, we want to share our “tricks” to achieve the best possible images. Nevertheless, many attempts are usually necessary to produce excellent results. In particular, the legs and wings of flies presented unique challenges in depicting a natural posture.

### Preparation of larvae

Flies were grown on a standard medium at room temperature. We ensured that the breeding tubes in which we kept our fly strains were never overpopulated and were cleaned to prevent debris from accumulating on specimens. Larvae in overcrowded conditions are known to be smaller than usual, which would be undesirable for our studies. We used animals for this atlas at the end of the third instar larval stage and in the migratory phase. These third instar wandering larvae were carefully picked up from the wall of the breeding vial and killed by submerging the entire animals in 60°C hot water for thirty seconds. This proved detrimental to ensuring that animals fully relax and straighten out. Larvae were fixed in 4% formaldehyde in PBT (PBS + 0.2% TritonX-100). Subsequently, animals were transferred into reaction cups and rinsed with five washes in phosphate-buffered saline [PBS (15 min each)]. After fixation, the specimens were dehydrated via increasing ethanol series.

### Preparation of pupae

The pupae were carefully detached from the substrate using a dissecting needle and spring steel tweezers. Pupae were extensively washed in PBS to clean the animals. Some pupae were additionally treated with one drop of dish soap to enhance the cleaning process. This drop of dish soap was added to all the PBS washing steps, including agitation for 10–15 min using an orbital shaker. PBS was changed at least five times or until no more impurities were visible on the surface of the puparium, using a stereomicroscope for control. After washing, the specimens were treated with an increasing ethanol series.

### Preparation of adult flies

Animals selected for electron microscopy remained in clean, fresh culture tubes for at least 24 h before preparation to largely avoid contamination of body surfaces with food particles or dirt. Flies were anesthetized and killed with diethyl ether. Afterward, the visual appearances of all specimens were evaluated under a stereomicroscope to exclude individuals with apparent defects that may have occurred upon handling. Flies with broken wings that were otherwise well preserved were used to image the intact body parts. For further treatment of adult flies, we followed Bag and Mishra's protocol, although we optimized several steps for our analyses (Bag and Mishra 2019). In brief, flies were fixed in 4% formaldehyde in PBT (PBS + 0.2% TritonX-100) for at least two hours before washing three times in PBS on an orbital shaker. Fixation could be extended for up to 24 h without impairing the quality of the results. After washing, the specimens were treated with an increasing ethanol series of 30%, 50%, and 70% for ten minutes each with agitation. Next, the flies were treated with 90% ethanol for 15 min and 100% ethanol for 10 min. The ethanol was then replaced with fresh 100% ethanol. At this stage, specimens could be stored at 4°C for weeks or months.

### Critical point drying

For Critical point drying (CPD), all samples were transferred from ethanol to liquid carbon dioxide. We used a Leica CPD 300 at 10°C cooling temperature with “CO_2_ in” set at medium, “exchange” at 24, “heat” at 40°C, and “Gas out” at fast.

### Fixation of specimens on sample stands

After CPD, specimens were placed on SEM stumps prepared with double-sided carbon stickers to hold them in place. This was one of the most critical steps. The carbon stickers provide a firm grip on the samples. However, the initial placement on the stumps had to be perfect, since removing flies, or individual structures, from the adhesive surface, often resulted in the deformation or breaking of structures.

### Sputtering

Larvae and adult flies were then sputter-coated with gold top-down and at a 15° angle. The first coating produced a 15 nm thickness, and the coating at an angle produced a 5 nm thickness. A Leica ACE600 was used for gold vapor deposition.

### Acetone spraying for imaging of complete adult flies

During our work, we discovered that the supposedly most straightforward images—such as the fly with its legs and wings in a natural position—were the most difficult to obtain. For these images, we had to deviate from the above-mentioned procedure and optimize the anesthetizing and killing of the adult animals specifically for our imaging purposes. Lightly anesthetized flies were transferred into a large Petri dish, where another, smaller Petri dish was placed, to fix the fly in its natural body position. This smaller Petri dish contained a paper strip soaked with chloroform. Using two dishes was essential to ensure the flies did not contact the chloroform. The flies were kept in this environment for 3–5 min. Using this method, the legs and wings occasionally, but not always, remained in their natural positions. The flies were then transferred to a stack of conventional paper sheets and sprayed with acetone until thoroughly soaked. This step was performed with care and under a fume hood. Each specimen had to be sprayed with acetone 4–6 times. Each time, we had to wait until the acetone evaporated before adding a new spray layer. It is essential to spray not only on top of the flies but also to alter the angles to ensure the coating of the whole specimen. Failure to achieve this would destroy specimens once they entered a vacuum chamber. Once spraying was completed and the flies were immobilized, they were mounted on stumps. Optimal positioning and alignment were attained by carefully placing the specimen on the adhesive carbon stickers on the SEM stump. Afterward, specimens could be inserted into the sputter chamber immediately without CPD. This way, we imaged the fly in its natural crawling position.

### Imaging and image processing

All our images were captured with the JEOL-JSM-IT200 microscope, exported as TIFF files, and processed using Affinity Photo. Voltages for imaging varied from 10 to 15 kV with a typical working distance of 13 mm. Image plates were arranged and labeled using Affinity Designer. For some overview images, mosaics of several independent images were assembled using Fiji (Schindelin *et al*. 2012).

### Anatomical features of the larvae

The larvae of flies, commonly known as maggots, constitute worm-like animals of about 1 to 5 mm in length. In many species, larvae exhibit a pale white to nearly translucent appearance. *Drosophila melanogaster* larvae undergo three distinct developmental stages—L1 to L3. The first instar (L1) develops during embryogenesis and directly hatches out of the embryonic chorion and vitelline membrane by the end of embryogenesis. After about 24 h, the first instar sheds its old cuticle and is referred to as the second instar (L2), which undergoes another molting after an additional 24 h. Before entering metamorphosis, the third larval stage (L3) persists for about 72 h with no additional molting. However, the animals grow substantially during this final larval stage and become highly mobile just before pupation. Hence, the terminal stage of larval development is often called “wandering larva.” This can easily be observed in healthy *Drosophila* cultures, as many animals usually start crawling on the walls of the culture vials shortly before they pupate. The most apparent difference between the larval stages might be the size of the animals. Nevertheless, there are more subtle changes to the outer morphology of the larvae as they further develop. As one of many examples, the morphology of the anterior mouth hooks, the “teeth” of the larvae, undergo visible changes during larval development (Demerec 1950; Wipfler *et al*. 2013).

Since this paper focuses on the morphology of the imago, we will not cover the morphological details of each larval stage here. Nevertheless, larvae serve as an important model system for studying basic genetic principles of development, such as sensation, segment patterning, axis formation, and planar cell polarity. Thus, we will briefly overview the primary outer anatomy of the third larval stage.

#### The outer anatomy of the larva

**Fig. 1.**
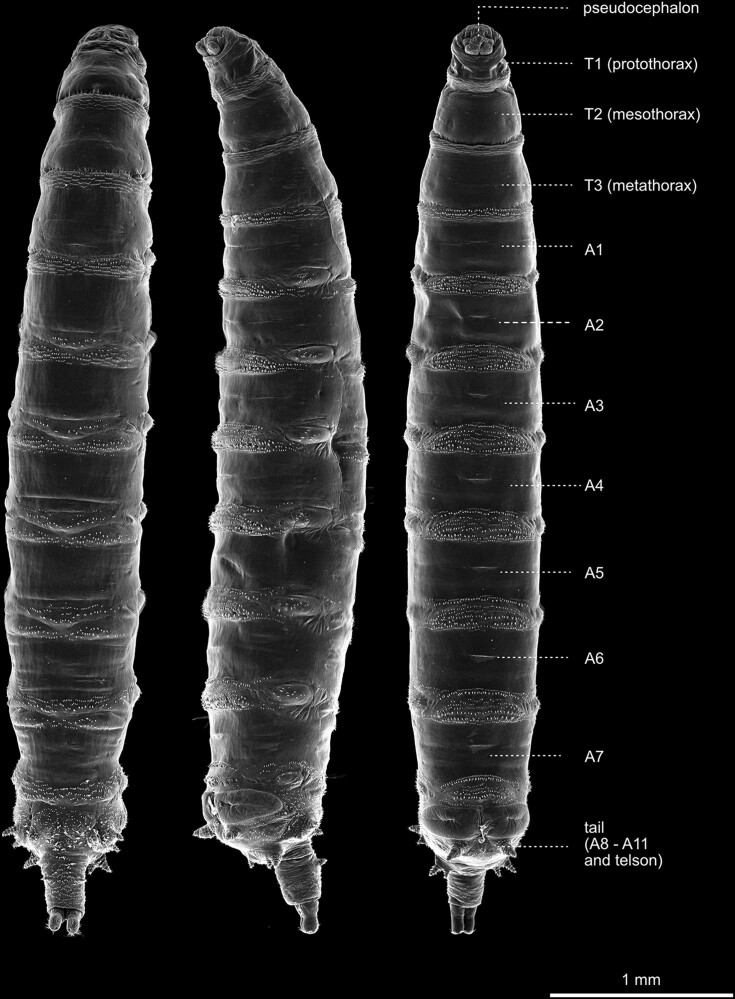
Overview of the habitus of the third instar larva. The animals are viewed from dorsal (left), lateral (middle), and ventral (right). The anterior of the animals is aligned to the top and posterior to the bottom. The ordered appearance of denticle belts indicates the metameric order of segments. The respective segments are annotated in the right-most animal. The segment borders roughly coincide with the denticle belts, while the majority of each posterior compartment in each segment carries denticles (see details in Figs. 2 and 3). In the thoracic segments, the rows of denticles are thinner, and individual denticles are smaller and display a relatively uniform appearance. The dorsal denticles are less dense and ordered than the ventral side. The prominent bulges along the lateral side of the animals correspond to the attachment sites of body wall muscles (see Fig. 2). At the anterior, the pseudocephalon can be seen from lateral (middle image) and ventral (right picture), but is obstructed by the prothorax from a dorsal perspective (left image). At the posterior, the abdominal spiracles can be seen at the very tip of the animal, while the anal pads are visible from ventral and lateral views but not from the dorsal (for details, see Fig. 5).

**Fig. 2.**
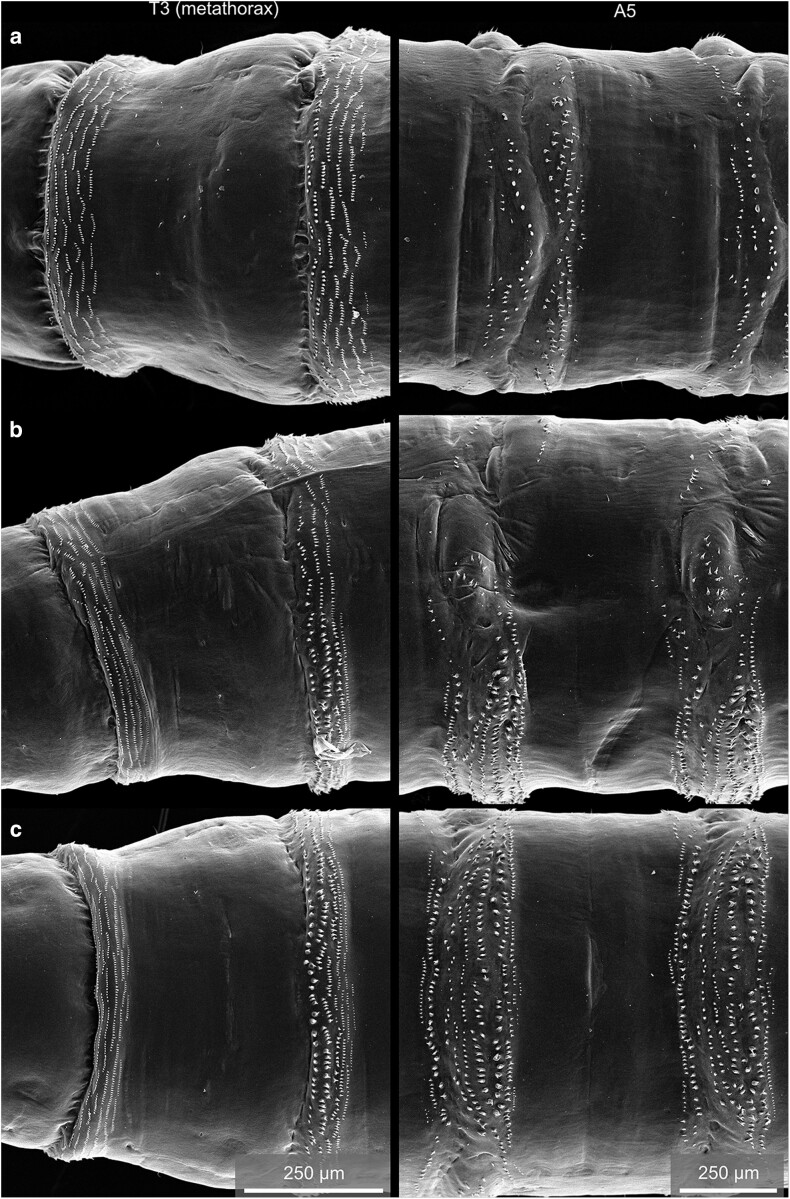
The denticle belts of the larva. Overview of the denticle belts of the metathorax (T3) and abdominal segment A1 (left) and the denticle belts of the abdominal segments A5 and A6 (right). The animals are viewed from dorsal a), lateral b), and ventral c). In all segments, the denticle belts localize to the segment border, where the posterior compartment of one segment overlaps with the anterior compartment of the following one (Saavedra *et al*. 2014). Thus, the genetic segment boundary runs through the denticle belt itself. However, the denticle belts can serve as morphological landmarks to identify the segment borders. In the thoracic segments, the denticles appear very uniform in size and orientation. They are organized in rows resembling palisades, mostly pointing toward the posterior. The following abdominal segments display denticles that vary in size and orientation, depending on the identity and planar polarization of the epidermal cells that form the respective denticle. While the dorsal belts are relatively sparse in the number of denticles, at the ventral side, denticles form uneven rows of densely packed structures.

**Fig. 3.**
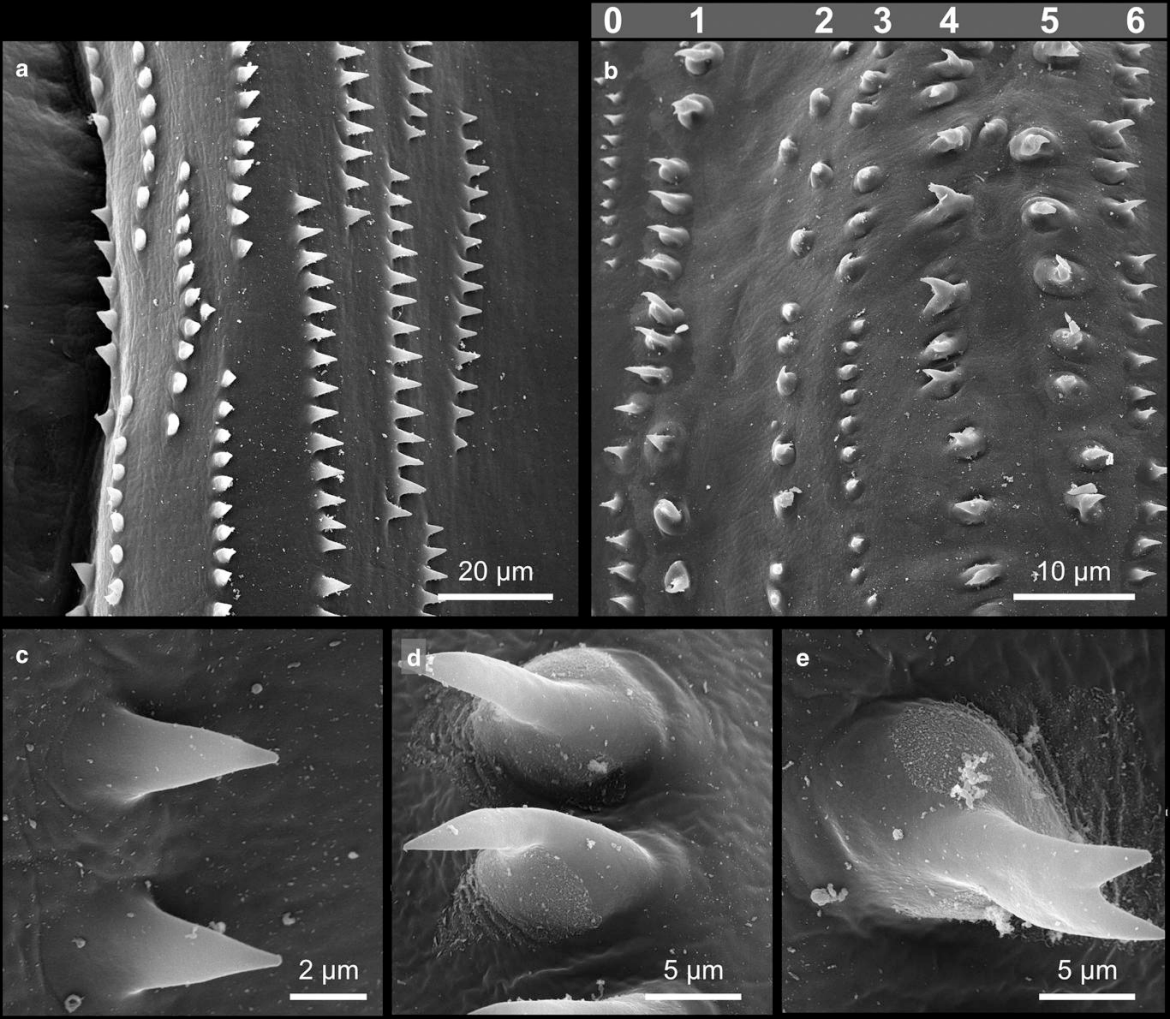
Detailed morphology of larval denticles. High magnification views of the ventral cuticle with denticle belts in the metathorax T3 a) and abdominal segment A5 b). In the metathorax a), the morphology of individual denticles displays only minor variations. In the most anterior row, denticles appear as sharp, triangular structures that point toward the anterior. All other metathoracic denticles point posteriorly c). The most anterior row is followed by 2–3 rows of stubby denticles. Finally, the last 4–5 rows of denticles appear, similar to the anterior ones (detail in bottom left). In the abdominal segments b), denticles are more variable in size, form, and orientation. Abdominal denticles are organized in seven uneven rows [row 0–6, (Saavedra *et al*. 2014)]. The denticles in rows 0, 1, 4, and 5 point toward the anterior, while those in rows 2, 3, and 6 point posteriorly. This orientation is due to the polarity of the epidermal cells below and has been extensively used to study the mechanisms underlying planar cell polarity. While the denticles in row 0 are small and display a similar morphology compared with thoracic denticles, denticles in other rows are substantially larger and exhibit a hook-shaped appearance, with a clear base and a 1- or 2-pointed sharp end d) and e).

The most obvious hallmark of the larval habitus is likely the pronounced segmentation of the animal along its anterior-posterior axis, which is most notably highlighted by the metameric arrangement of bristles and denticle belts along the body (Fig. 1). The larval body exhibits ten prominent trunk segments—3 thoracic segments (T1–T3) and seven abdominal segments [A1–A7 (Wipfler *et al*. 2013)]. The segment borders along the body can be clearly distinguished by the repetitive arrangement of denticle belts. Denticles constitute chitinous structures secreted by epidermal cells that adopt a triangular shape, with pointy hooks at their most distal ends (Figs. 2 and 3). The pattern of these belts and the orientation of each denticle varies along both body axes of the animal and are specific for each segment. At the ventral side of the larva, thin rows of small denticles localize to the segment borders of the thoracic segments (T1–T3), interlaced by stretches of naked cuticle (Figs. 2 and 3). The ventral denticle belts of the abdominal segments (A1–A7) are arranged in a pattern resembling a trapezoid and display specific orientations within the individual rows. Usually, seven rows of denticles are found at the border between each abdominal segment [Figs. 2 and 3 (Saavedra *et al*. 2014)].

**Fig. 4.**
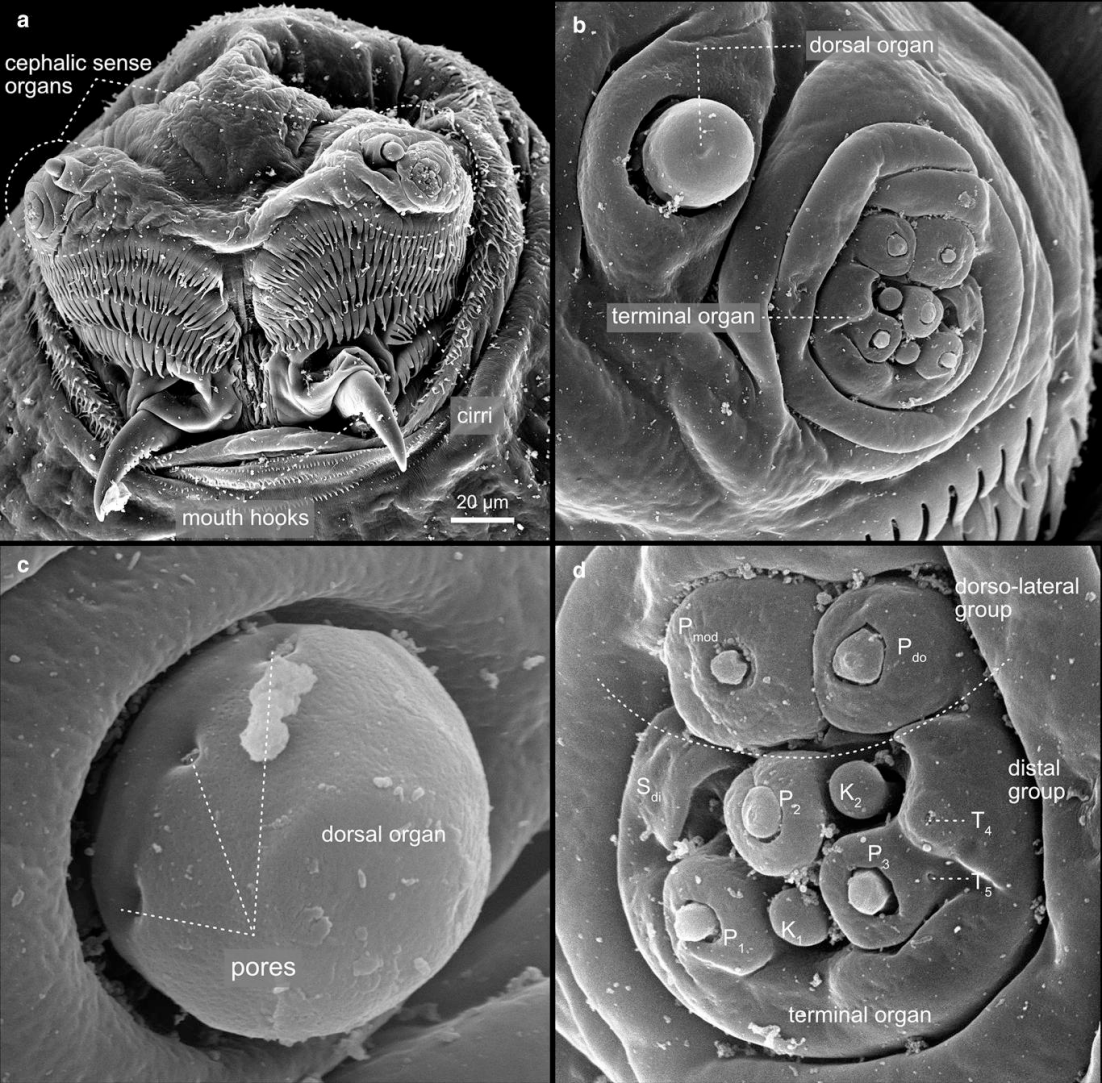
Detailed morphology of larva head. a) Anterior side with mouth opening, mouth hooks, and sense organs; dorsal side is up, ventral side is down—caudal view. The cirri surround the mouth opening. Two mouth hooks, which are used to hold on various substrates. The mouth hooks are cuticular buildings of the epidermis. b) The bilaterally cephalic sense organs belong to the chemosensory system of the larva. c) and d) Each cephalic organ is composed of a dorsal and a terminal organ. c) The dorsal organ is the primary olfactory organ of the larvae. Olfactory sense neurons project into the dome with multiple tiny perforations (not visible) and the base part of the dorsal organ. Three of the seven molting pores (pores), which are remains of ecdysis, are visible. d) The terminal organs house several taste neurons. Fourteen external sensilla, not all of them are visible in the image, with different morphologies, appearing as papilla-, pit-, knob- or spot sensilla are grouped into a dorsolateral group with two chemosensory organs and a distal group with several chemosensory organs. For a detailed description of the dorsal and the terminal organ, we refer to current literature (Apostolopoulou *et al*. 2015; Richter *et al*. 2023). P, Papilla; K, Knob; S, Sensilla; T, Pit.

**Fig. 5.**
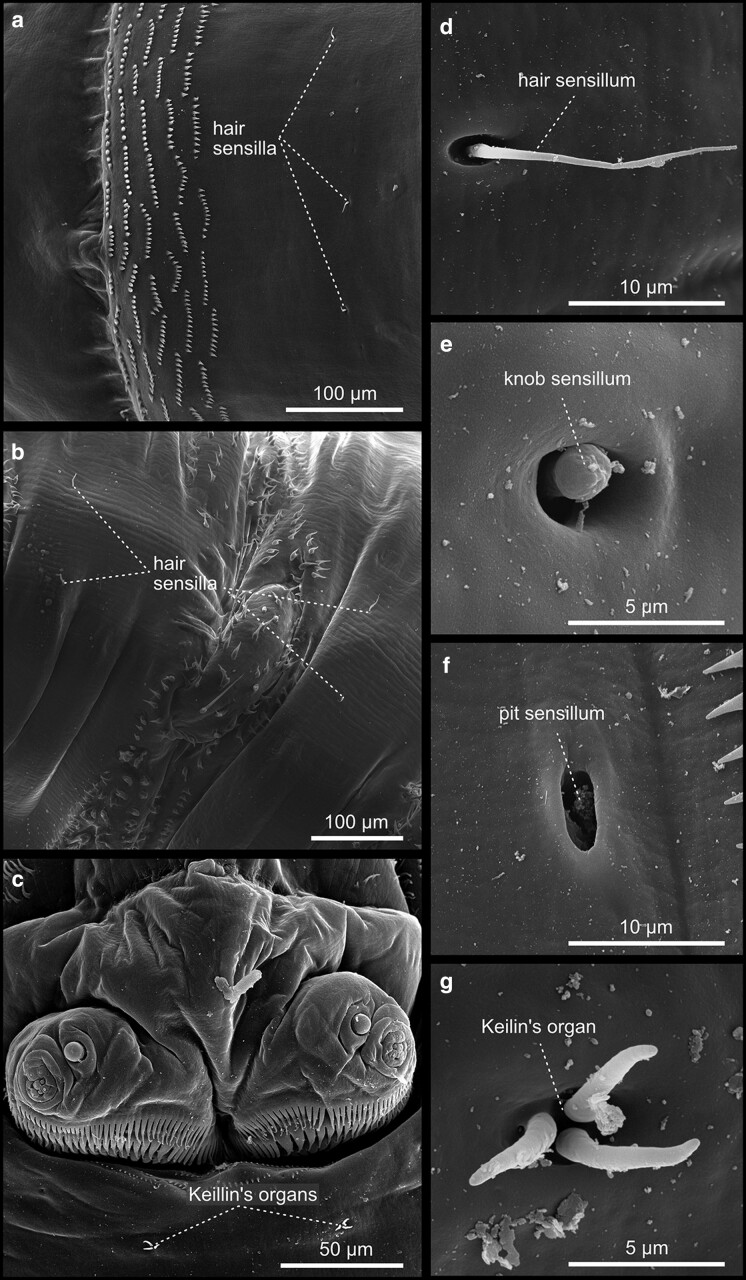
Larval sensilla. a) and b) The larva hosts several externally visible sensilla, which are responsible for different types of sensation. Here, we show some examples of typical sensilla that appear in the form of hair-like d) or knob-like e) structures or as a depression (pit) f) with or without an internal dome structure. c) Keilin's organ, named after the first person to describe it, is known in the literature as a hygrosensory receptor. Keilin's organ occurs in pairs on each thoracic segment and consists of a pit-shaped depression from which 3 hair-shaped sensilla arise g).

In addition to the trunk, the larva exhibits two terminal structures—the anterior pseudocephalon and the posterior tail. These most distal termini of the larva, or if you like the head and the tail, are built by rudimentary segments that undergo a complex development (Jürgens 1987). Although larvae appear acephalic (without a visible head), the anterior part of the animal develops from a head compartment, comprising 7 terminal segments determined during embryogenesis (Diederich *et al*. 1991). However, the head primordium relocates into the larval body through a process referred to as head involution. A series of complex morphogenetic movements characterize head involution. For details, we refer to (Jürgens and Hartenstein 1993). Consequently, only a few head structures remain on the surface of the larval head (also called pseudocephalon). The most recognizable structures include the dorsal (antennal) and terminal (maxillary) sense organs, the cirri, the prominent mouth hooks, and the actual mouth opening (Fig. 4). Furthermore, the pseudocephalon carries a variety of sensory papillae, receptors of various kinds that allow the animal to sense temperature, smell, taste, and navigate its environment (Komarov and Sprecher 2022; Richter *et al*. 2023). From a frontal view, the most prominent features seen on the larval pseudocephalon are the cirri, cuticular comb-like structures surrounding the animal's mouth opening (Fig. 4). Ventrally of four rows of cirri, the cephalopharyngeal sclerites, or mouth hooks, project out of the mouth opening (Fig. 4), which the larva uses to dig into, and move within the substrate. The pseudocephalon also harbors major sites for larval taste, smell reception, and thermosensation. The dorsal organ (also known as the antennal sensory organ) is composed of a prominent dome structure within a single socket (Fig. 4). Sometimes, molting pores can be observed at the dome's base (Fig. 4). Adjacent to the dorsal organ lies the more complex terminal organ, which comprises 14 external sensilla with different morphologies, appearing as papilla-, pit-, knob- or spot sensilla (Fig. 4). Along the animal's body, one can observe many more sensilla, which are responsible for various types of sensation and display varying morphologies (Fig. 5). These sensilla are classified by their morphology as hair sensilla, knob-type sensilla, or pit sensilla, which can all be found along the body surface of the larva (Fig. 5).

**Fig. 6.**
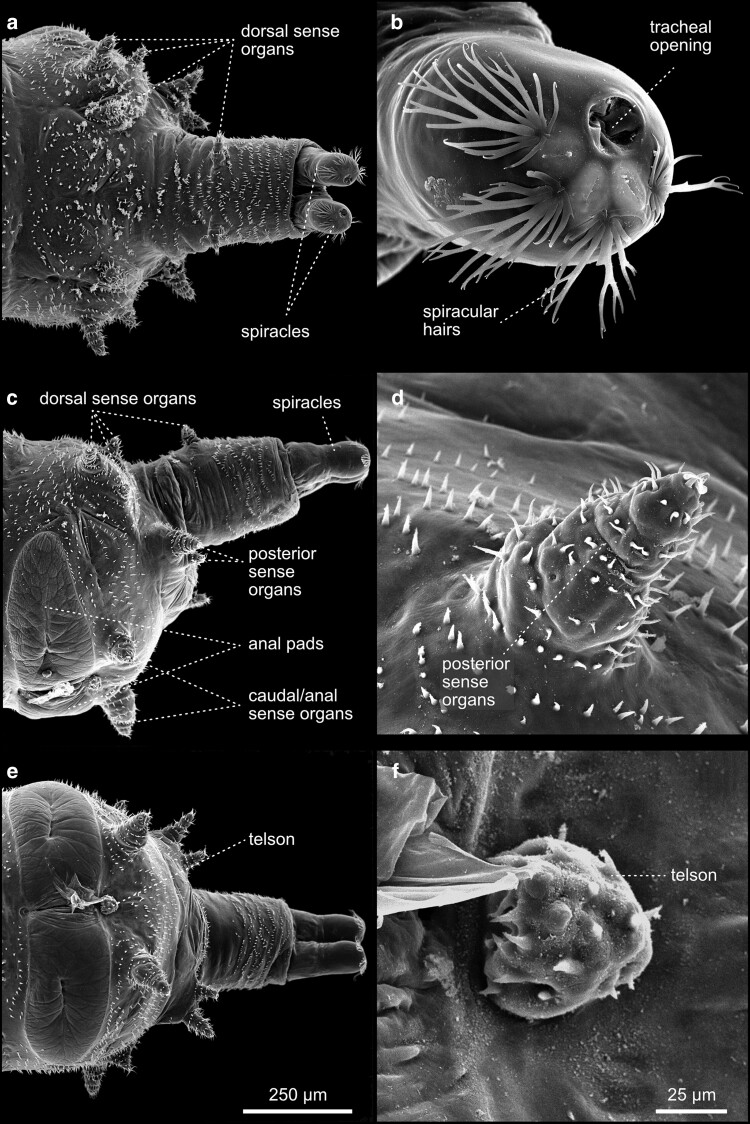
Detailed morphology of the larva tail. Features of the posterior, terminal segments of third instar larvae. Dorsally, a) the 2 spiracles of the tracheal system emerge from the animal. These spiracles harbor an opening, functioning as the exchange opening for respiratory gases b). Four tufts of chitinous spiracle bristles line the tracheal openings, which exhibit leave-like branches. c) Lateral view. The larval terminus harbors 16 cone-shaped sense organs (eight on each hemisphere), which are annotated according to their location in four dorsal, two posterior, and two anal sense organs on each side of the animal c). The organs share a similar organization and are built by concentric deposits of the cuticle, forming a peg-like organ. Each ring and tip is covered by a single row of denticles d). e) Ventral view. Segment A8 exhibits a different morphology compared to the other abdominal segments and contains two large anal pads, which surround the anal opening e). Posterior to the anus lies a tuft of denticles, which represents the true telson of the animal, and thus corresponds to the actual last segment of the larva f).

A morphologically particular type of sensilla is the paired Keilin's organs, composed of three sensilla emanating from a central pit (Fig. 5). These sensilla are located within the thoracic segments of the larva. It has been assumed that Keilin's organ serves as a hygro- and thermosensory organ (Hafez 1950; Benz 1956) or as a mechanosensory organ associated with crawling behavior (Lakes-Harlan *et al*. 1991). For further readings on sensilla, their internal morphology, and function, we refer to (Apostolopoulou *et al*. 2015; Friesen *et al*. 2015; Richter *et al*. 2023). The posterior end of the animal is built by segments A9–A11, the anal pads, and the telson [Fig. 6 (Jürgens and Hartenstein 1993)]. It is prominently characterized by 2 large abdominal spiracles, which represent the openings of the tracheal system and, thus, a major breathing opening of the larva (Fig. 6). The opening of the tracheal system localizes acentric at the distal tip of each spiracle. They are surrounded by four tufts of spiracular bristles (Fig. 6). Two anal pads surround the anal opening at the posterior in abdominal segment A8. The anal pads are surrounded by sense organs—4 dorsal, 2 posterior, and 2 anal (also called caudal) sense organs. These cone-shaped organs project out of the cuticle, each occupied by concentric rings of denticles and a distal sensilla tuft (Fig. 6). Finally, the anal opening is as well accompanied by a less pronounced sense organ, which represents the rudiments of the real final segment, the telson (Fig. 6).

### Anatomical features of the pupae

As a holometabolous insect, *Drosophila* undergoes a complete transformation from larva to adult, known as metamorphosis. In *Drosophila*, this developmental step becomes visible at the end of larval development through the white prepupa, when the transition from the mobile larva to an immobile state of development marks the beginning of pupation. During metamorphosis, numerous organs emerge that are only reserved for the adult animal, such as complex legs, wings, or eyes.

**Fig. 7.**
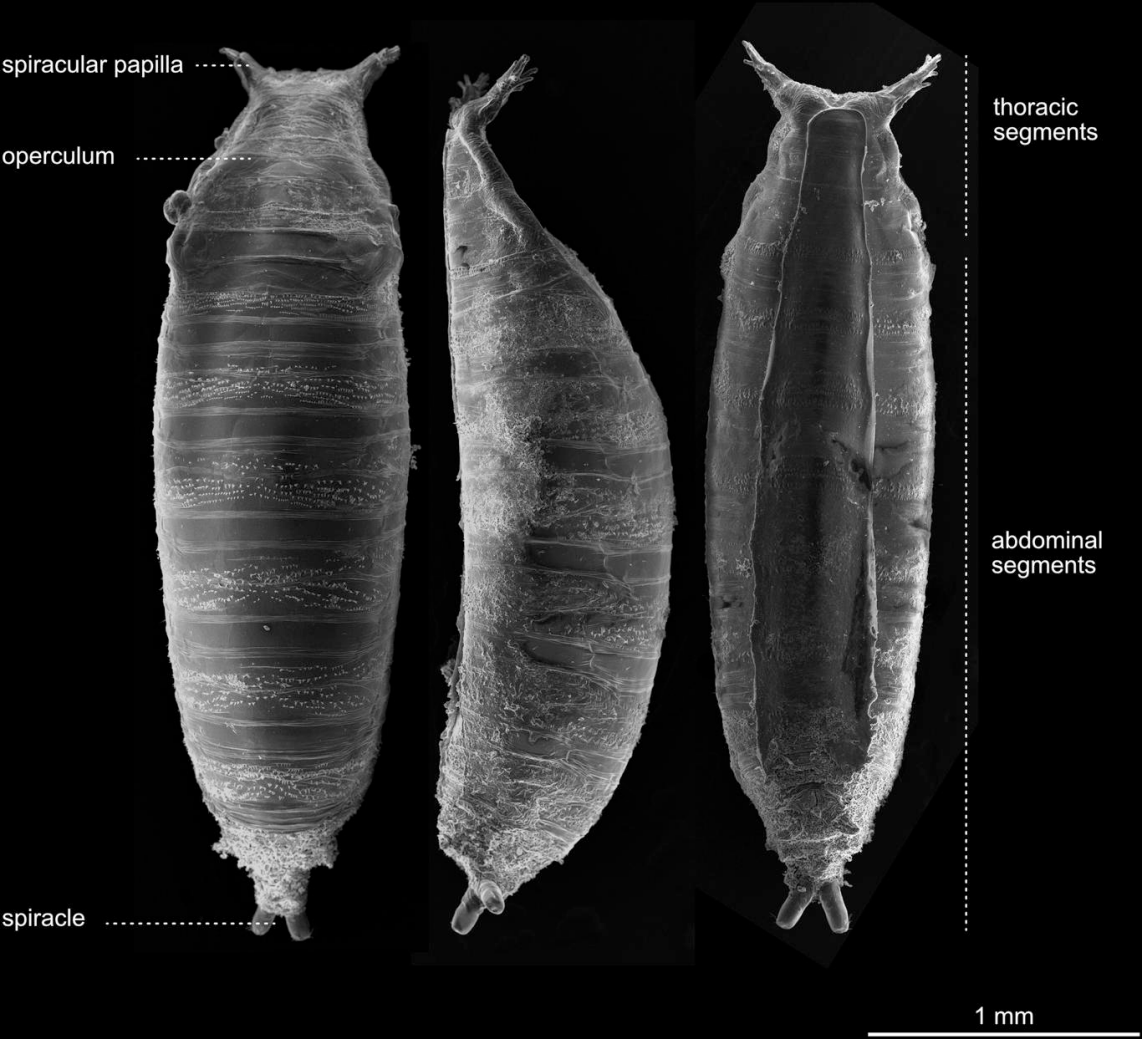
Anatomical overview of the pupae. Puparia from different views. Anterior is always to the top. a) Dorsal view. b) Lateral view. The remains of the surface structures of the former larval cuticle are still visible. c) Ventral view. Note that this pupa has been removed from the wall of a breeding vial. Thus, this image shows the surface area that has had contact with the plastic wall of the vial. A *Drosophila* pupa remains inside the exoskeleton of the third instar larva, which later becomes the puparium. About four hours after puparium formation, the animal inside has separated its epidermis from the puparium. The larval denticles, arranged in cuticle belts, are still visible at the surface of the puparium. The hatching cup serves as a circular aperture or lid through which the adult fly escapes the puparium (operculum). The hatching fly bursts the lid off with its frontal bladder, called the ptilinum, into which it squeezes hemolymph.

**Fig. 8.**
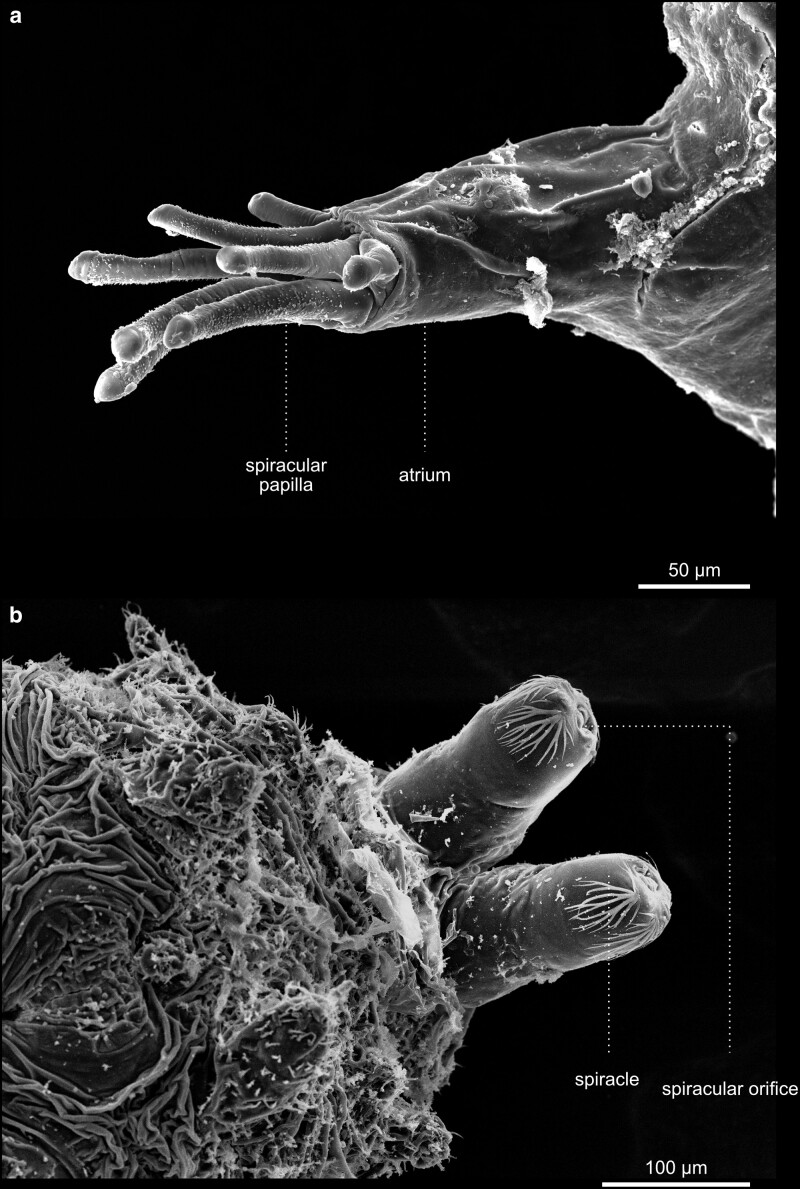
Pupal terminalia. Aspects of puparium anatomy. a) Close-up of the anterior spiracles. b) Close-up of the posterior spiracles. In larvae, spiracles are the anatomical structures for respiration. Here, the image shows the cuticular remains of the larval organs.

The adult fly ready to hatch, still in the puparium, is called the pharate adult (Figs. 7 and 8). The cuticle of the last larva forms the puparium. This begins with the formation of a procuticle. The larva shortens during this process and becomes immobile. The outer layer of the cuticle is tanned and forms a rigid structure known as a puparium. The formation of the puparium is often referred to in textbooks as pupariation. After the final apolysis, the head and appendages of the fly pupa or adult, which were previously hidden under the epidermis of the larva, are turned outwards, and the pupal cuticle is secreted. The 2 spiracular papilla formed by the pupa allow gas exchange even when the rest of the animal is surrounded by substrate (Fig. 8). Since this paper focuses mainly on the morphology of the imago, we will present only a view image of the outer structure of pharate adult pupae.

### Anatomical features of the adult

**Fig. 9.**
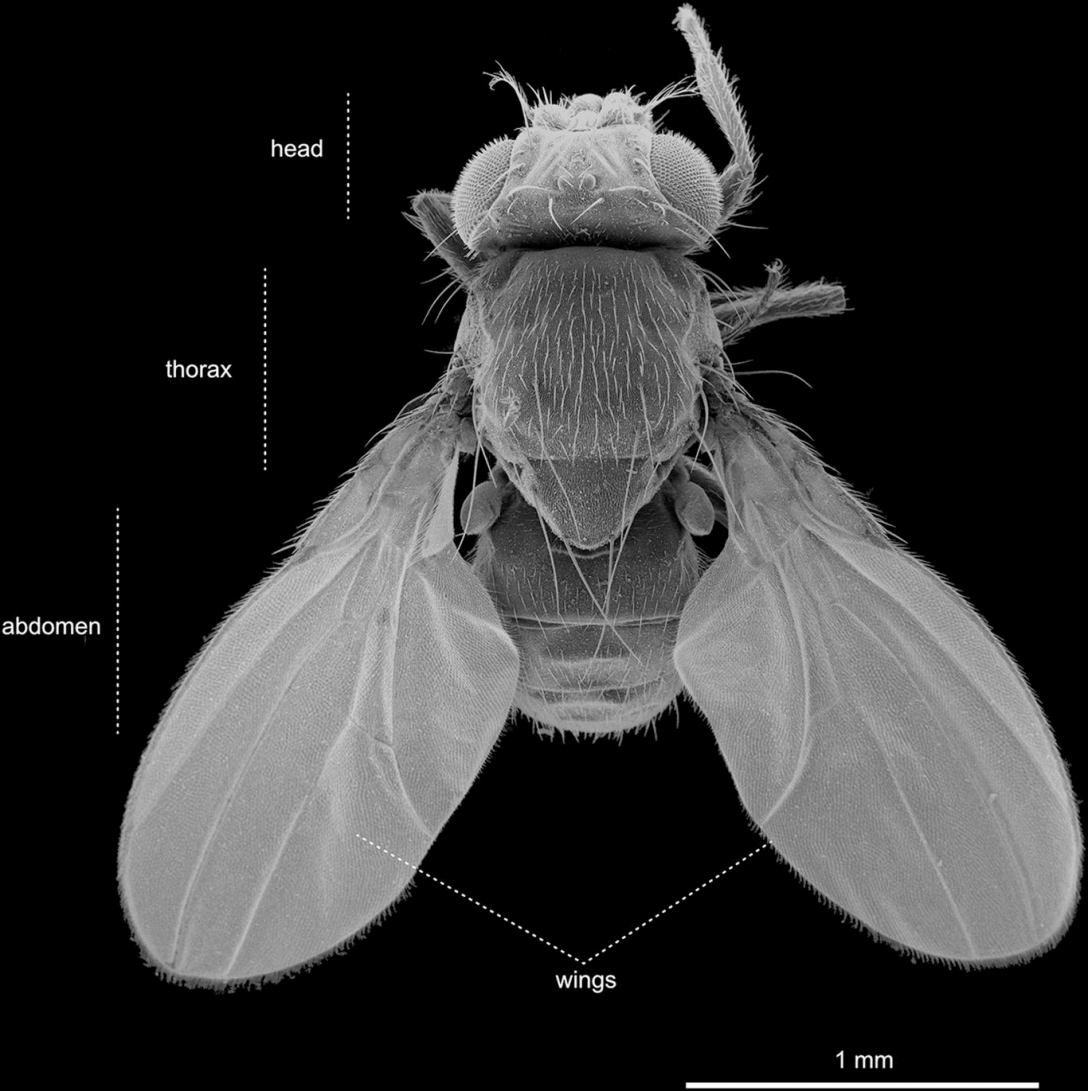
Dorsal view of an adult fly. The overview shows the three body parts of the fly: head, thorax, and abdomen. Scutum, scutellum, wings, and halteres are visible. The scutum forms the anterior part of the thorax and is a critical component in flight muscle attachment. The scutellum, located posteriorly, contributes to balance during flight. These thoracic segments are crucial for the fly's flight capabilities and coordinated movement. The bristles located at the posterior end of the scutellum in *Drosophila* serve as mechanosensory structures. These specialized sensilla detect mechanical stimuli and provide the fly with crucial information about its environment, aiding in flight control and navigation.

**Fig. 10.**
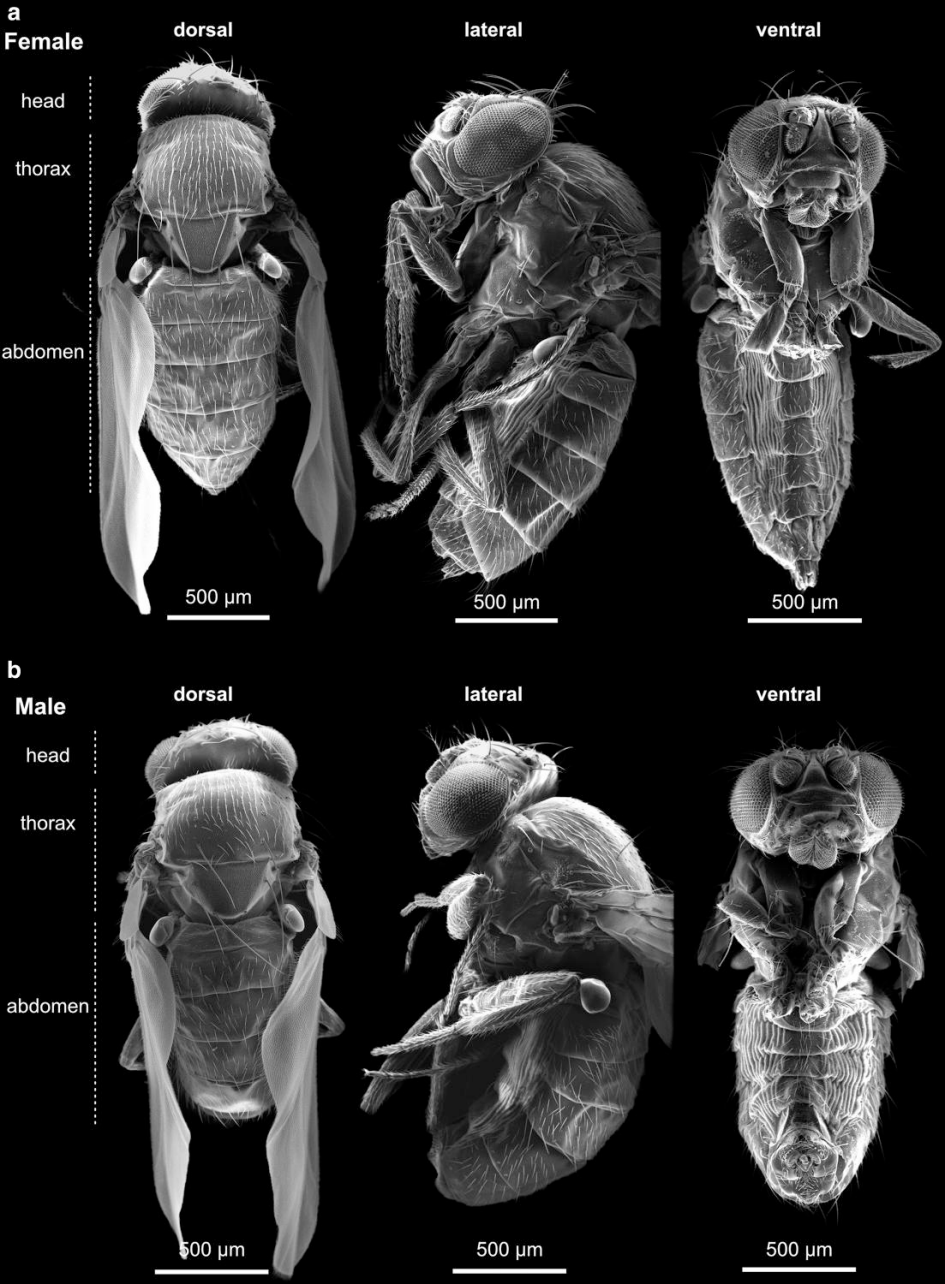
Overview of a) adult female and b) adult male specimens from dorsal (left), lateral (middle), and ventral (right) views. Anterior is always to the top.

As with all arthropods, the fly's imago divides into three major body parts: the head, the thorax, and the abdomen (Figs. 9 and 10). In the following sections, we will briefly discuss the significant morphological hallmarks of each body part and its appendages.

## The head

**Fig. 11.**
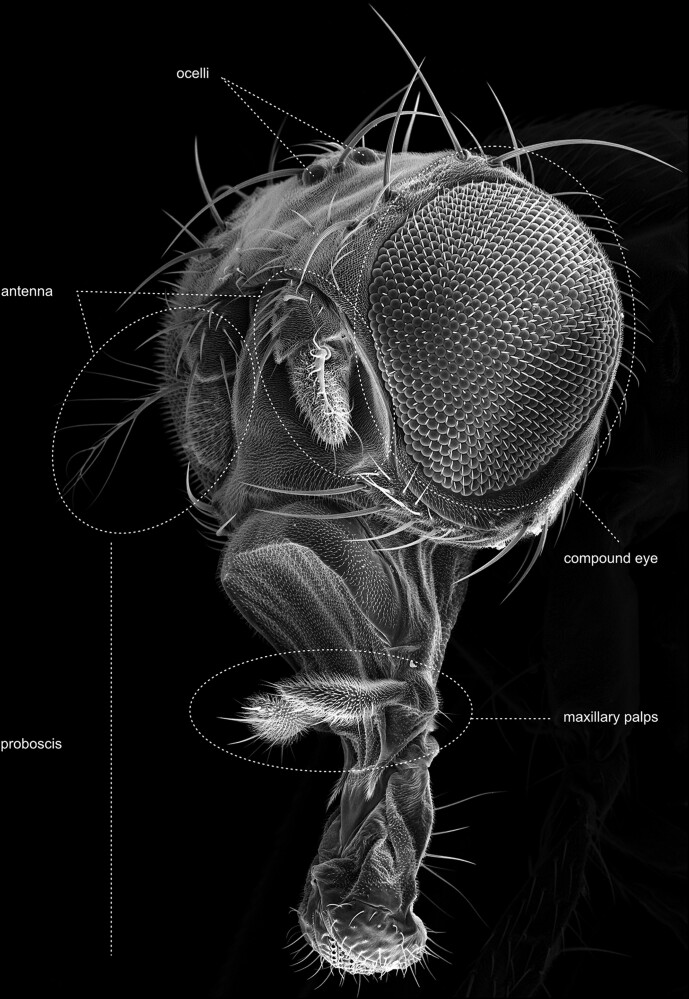
Head—main parts—overview. Oblique-frontal view of a dissected head and with evaginated mouthparts. The image illustrates the segmented organization of the head and general structures, including the mouthparts (proboscis and clypeus) and the head capsule with antennae, compound eyes, and dorsal ocelli.

The head of the fly comprises an acron (not considered as a segment) and 6 segments, bearing the primary sensory organs of the fly (Fig. 11). The 6 segments contain, from anterior to posterior, (1) the compound eyes, (2) the antennae, and (3) the clypeolabrum, which is built by the labrum and the clypeus. The labrum is a movable plate connected to the border of the clypeus, which is a frontal piece of the head. The fourth to sixth segment comprises three pairs of mouth appendages: (4) the mandible, (5) the maxillae with maxillary palps and lobes, and (6) the labium. In flies, the maxillae form an elongate sucking tube, called the proboscis. The ground pattern of the labium is modified distally into a pair of pronounced porous lobes, the labial palps [labellum (Fig. 11)].

**Fig. 12.**
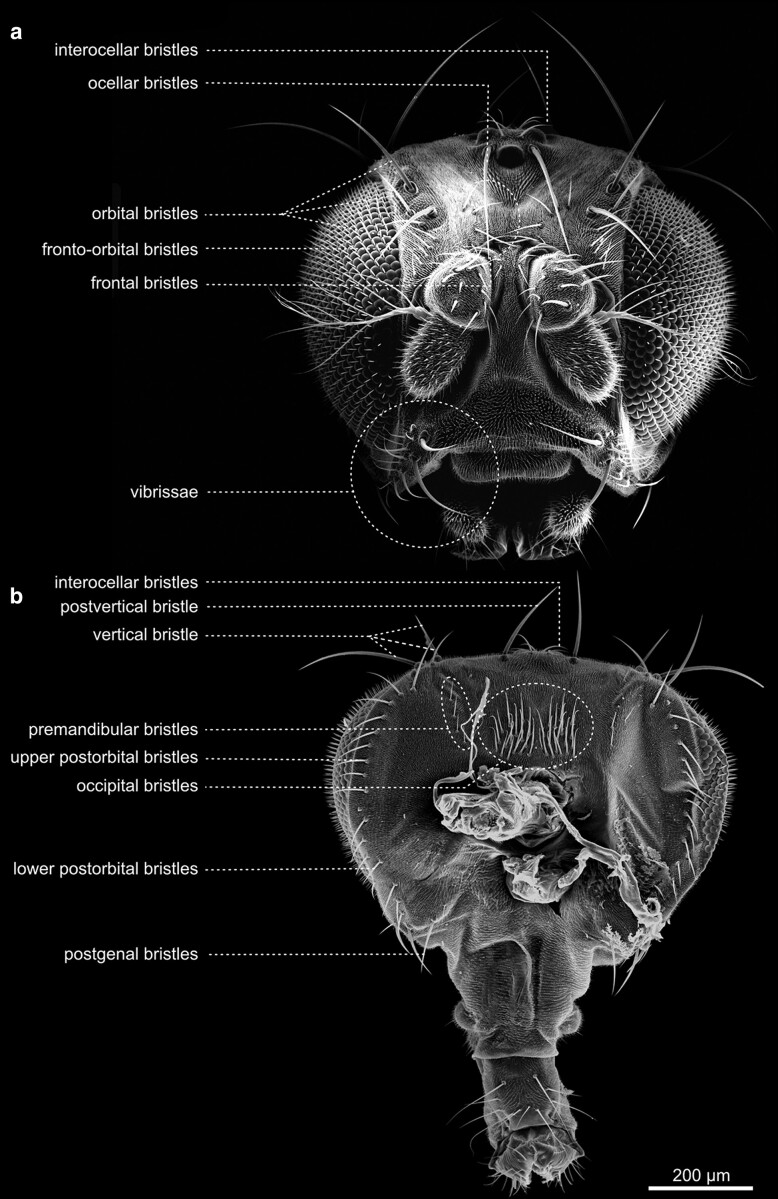
Head—frontal and caudal view—chaetotaxy. a) Frontal view of the head with mouthparts retracted. b) Caudal view of a head detached from the thorax. The proboscis is seen from the back. The head hosts several visible sensilla, which are responsible for different types of sensation, mostly mechanoreception. All bristles are arranged stereotypically.

**Fig. 13.**
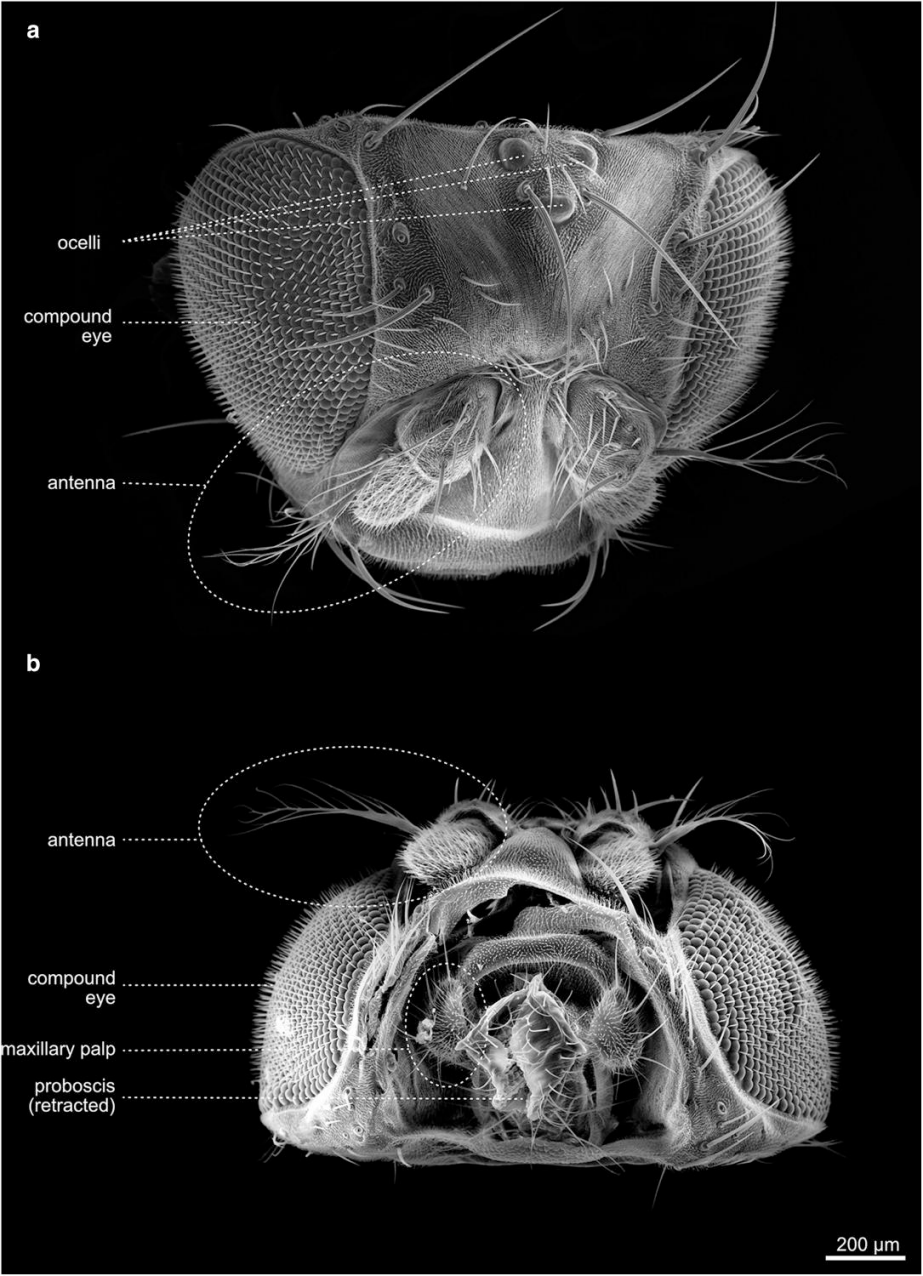
Head—Dorsal and ventral view. Dorsal a) and ventral b) views of the *Drosophila* head. a) Dorsolateral view of the head, prominently illustrating the compound eyes of the fly, as well as the three dorsal ocelli and the anterior antennae with aristae. b) The ventral view of the head shows the invaginated proboscis with prominent labium and maxillary.

The most prominent features of the fly's head are likely the large compound eyes. Except for some parts of the proboscis (mouthparts) and the eyes, the entire head is densely covered by tiny bristles (microtrichia). Furthermore, it exhibits a stereotyped pattern of more robust bristles (Fig. 11). The bristles vary in size and occur isolated or in groups. The largest bristles are the vertical bristles on the hindhead and the orbital bristles on the forehead. The smaller bristles include the occipital bristles on the back of the head and, for example, the bristles on the maxillary palps. All bristles are named according to their position on the head segments. There are antennal bristles, frontal bristles, frontal-orbital bristles, interocellar bristles, lower postorbital bristles, occipital bristles, ocellar bristles, orbital bristles, palpal bristles, postgenal bristles, premandibular bristles, postvertical bristles, postorbital bristles, vertical bristles, vibrissae, labial bristles, and several more (Figs. 12 and 13). The morphogenesis of the adult head has been described in several publications, for example, in the study by Haynie and Bryant (1986).

###  

#### The eyes and Ocelli

**Fig. 14.**
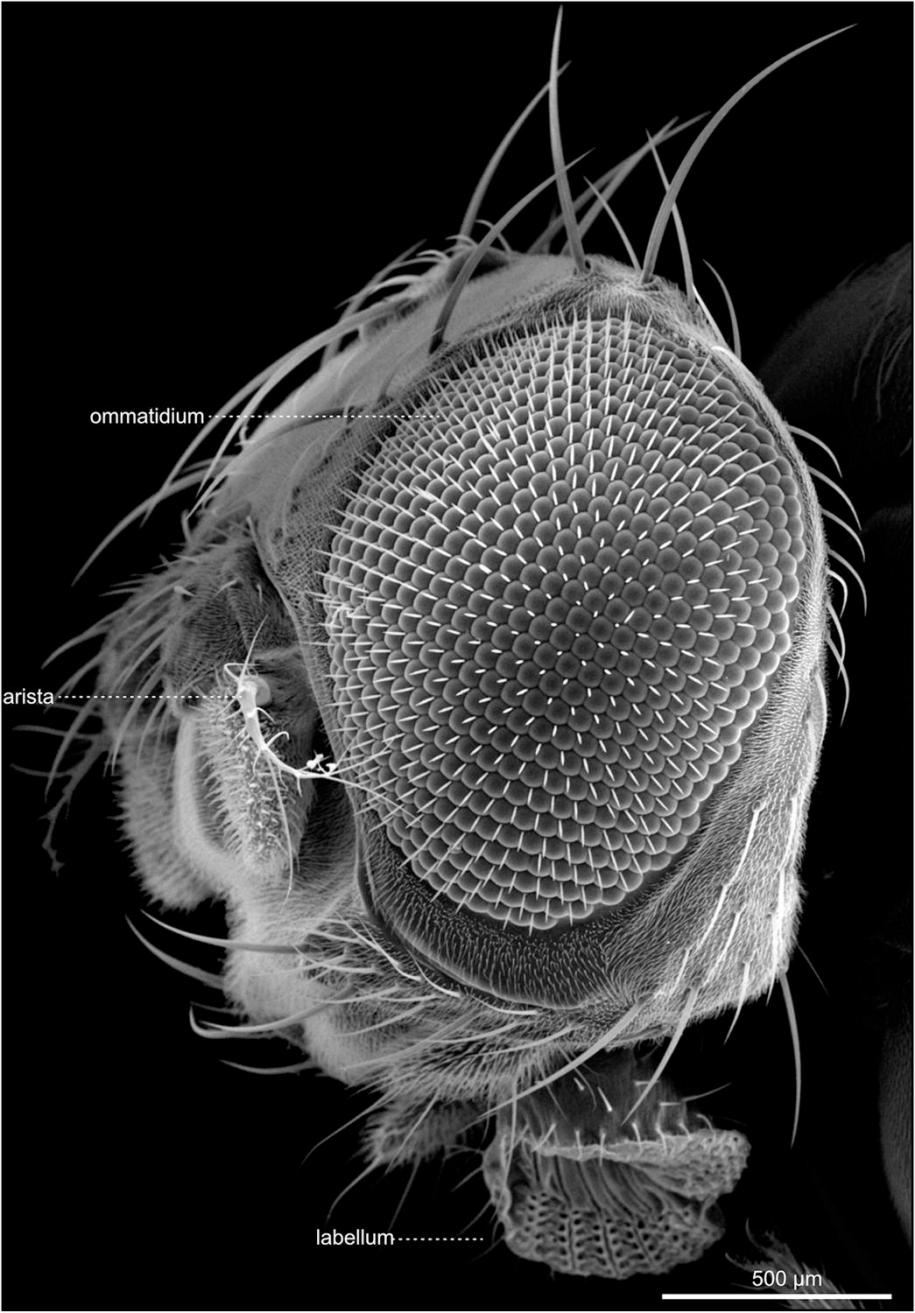
Head—compound eye—overview. High magnification view of the *Drosophila* compound eye. The eye constitutes a semi-sphere, with about 700 (male) to 750 (female) single ommatidia, each accompanied by a single mechanosensory bristle. Each ommatidium contains about 20 specific cells, including eight photoreceptor cells. On the eye's surface, each ommatidium consists of a transparent chitinous cuticle, which forms the cornea.

**Fig. 15.**
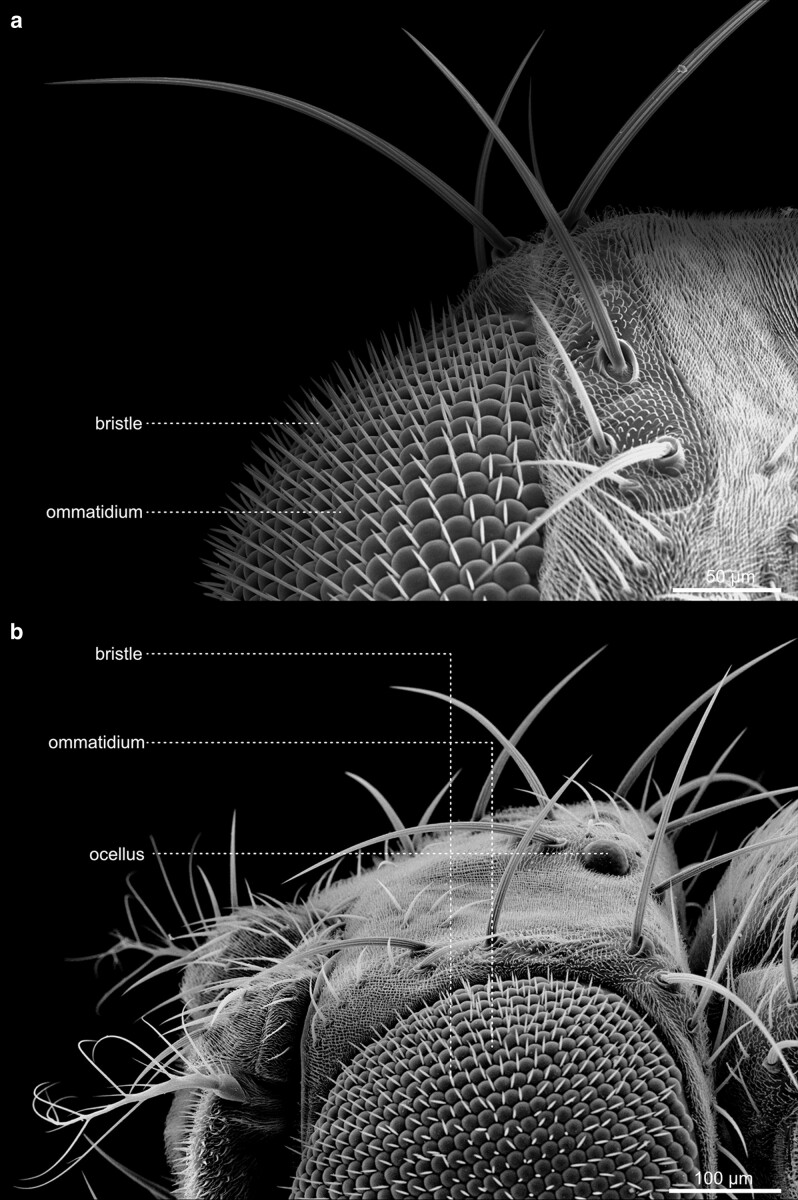
Head—compound eye—details. a) Dorsal view of the head. The image illustrates the large dorsal bristles at the eye's border. Each mechanosensory organ consists of four cells: the socket cell (so), the shaft cell (sf), the sheath cell (st), and the neuron, with the first three being visible. b) Lateral view. The image features the dorsal bristles and anterior aristae of the antennae. It also shows the transition of the head into the thorax of the animal.

**Fig. 16.**
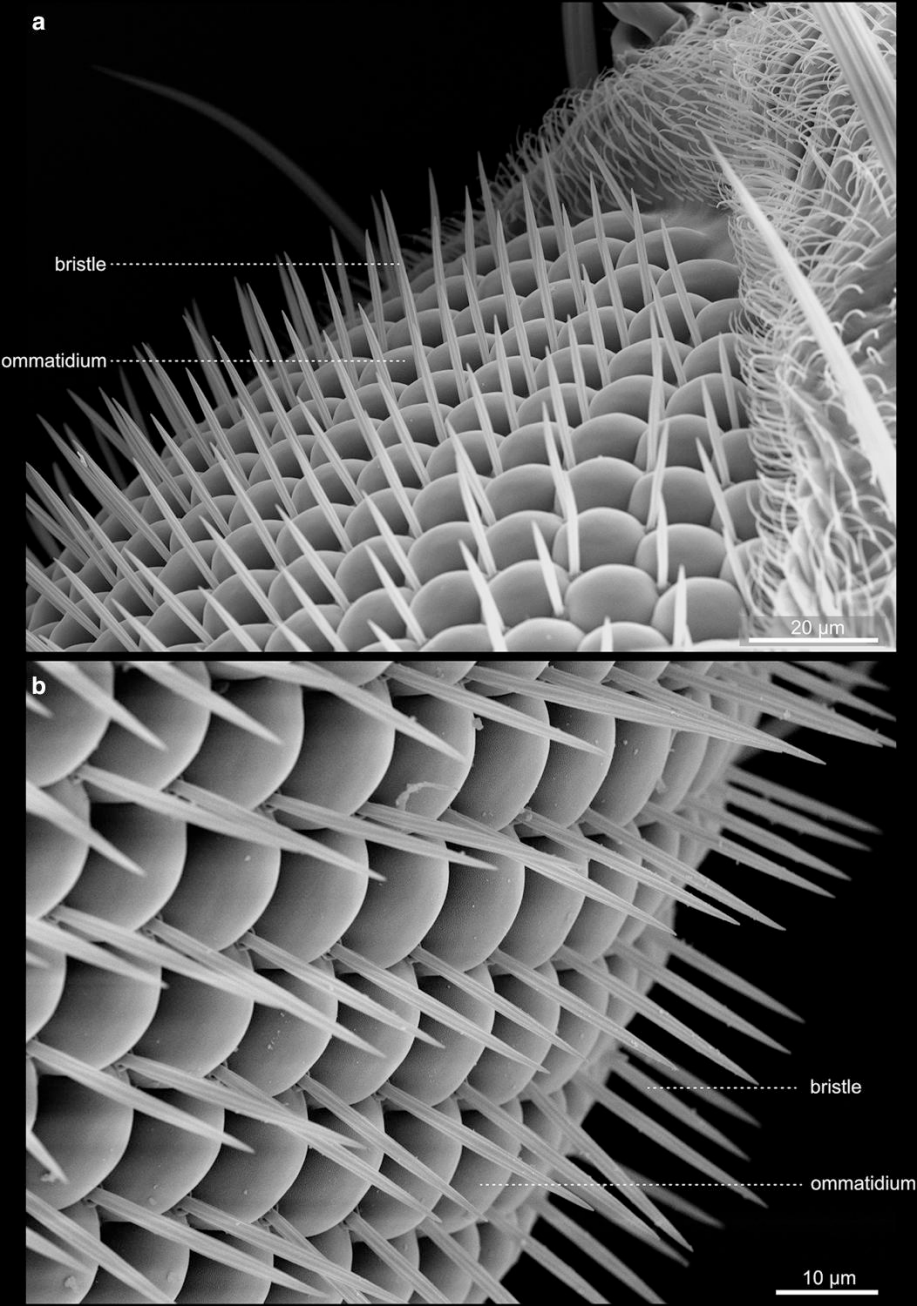
Compound eye—Ommatidia. a) and b) Ommatidia and bristles, from different views. The image illustrates the regular organization of ommatidia throughout the organ.

**Fig. 17.**
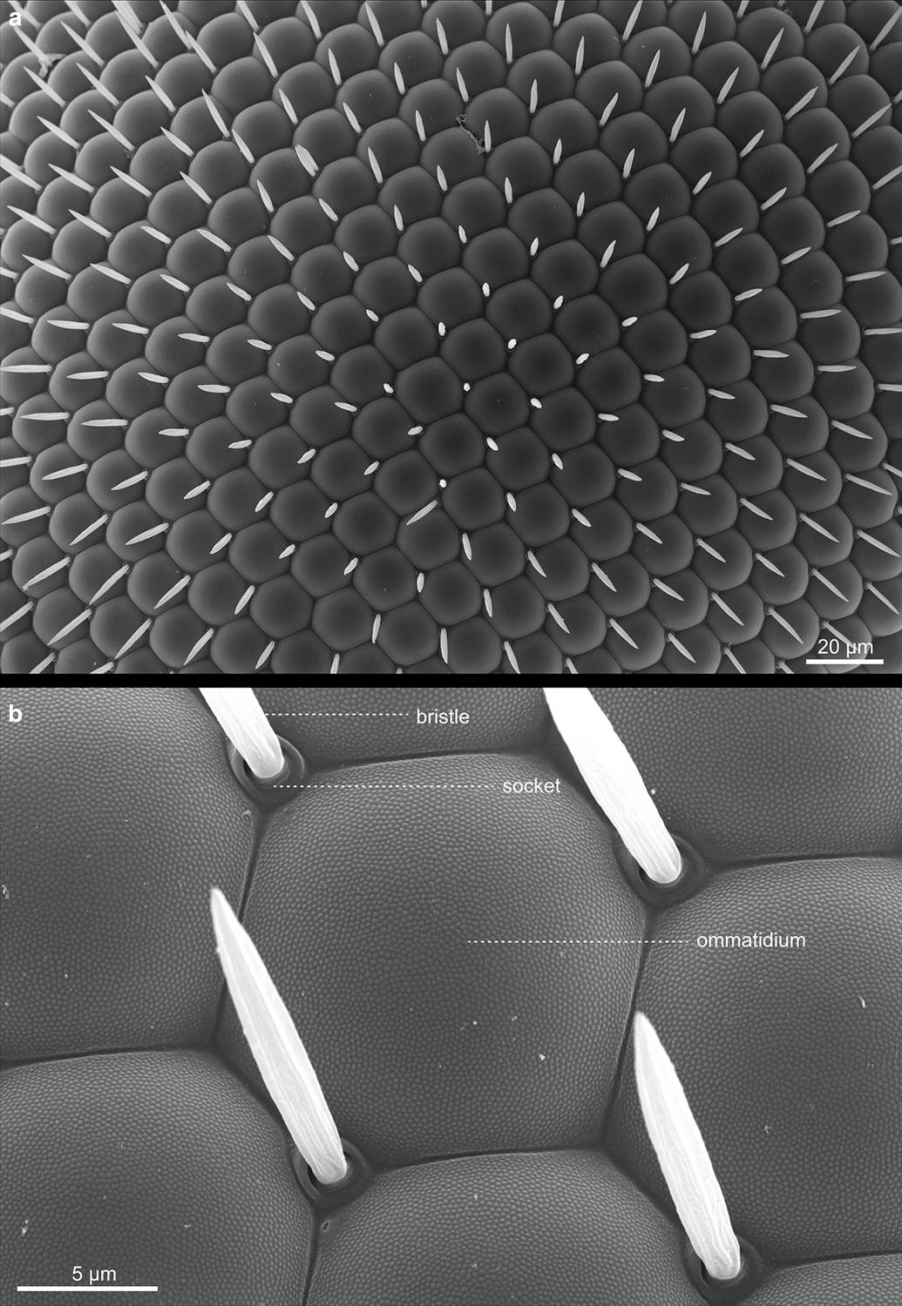
Compound eye—Ommatidia—detail. a) Overview, Ommatidia, and bristles seen from the top. b) High magnification views of a single ommatidium, showing the hexagonal organization of a single cornea and the intermediary mechanosensory bristles.

The compound eyes contain approximately 700 (male) to 750 (female) single ommatidia, or facets, and are situated within the orbital domain of the vertex, laterally at each side of the head capsule [Figs. 14–17 (Jean-Guillaume and Kumar 2022)]. While the number of ommatidia per eye is slightly variable in the wild-type, their pattern within the retina is highly ordered (Cagan 2009). Each ommatidium exhibits a nearly perfect hexagonal shape. Furthermore, each facet harbors a single sensory bristle, usually localized at its posterior corner (Fig. 17). Of note, there can be variations to this pattern, where sometimes a single bristle is located at a different position, or two bristles are formed between two facets (Fig. 16). However, such imperfections are relatively rare compared with the hundreds of facets per eye.

**Fig. 18.**
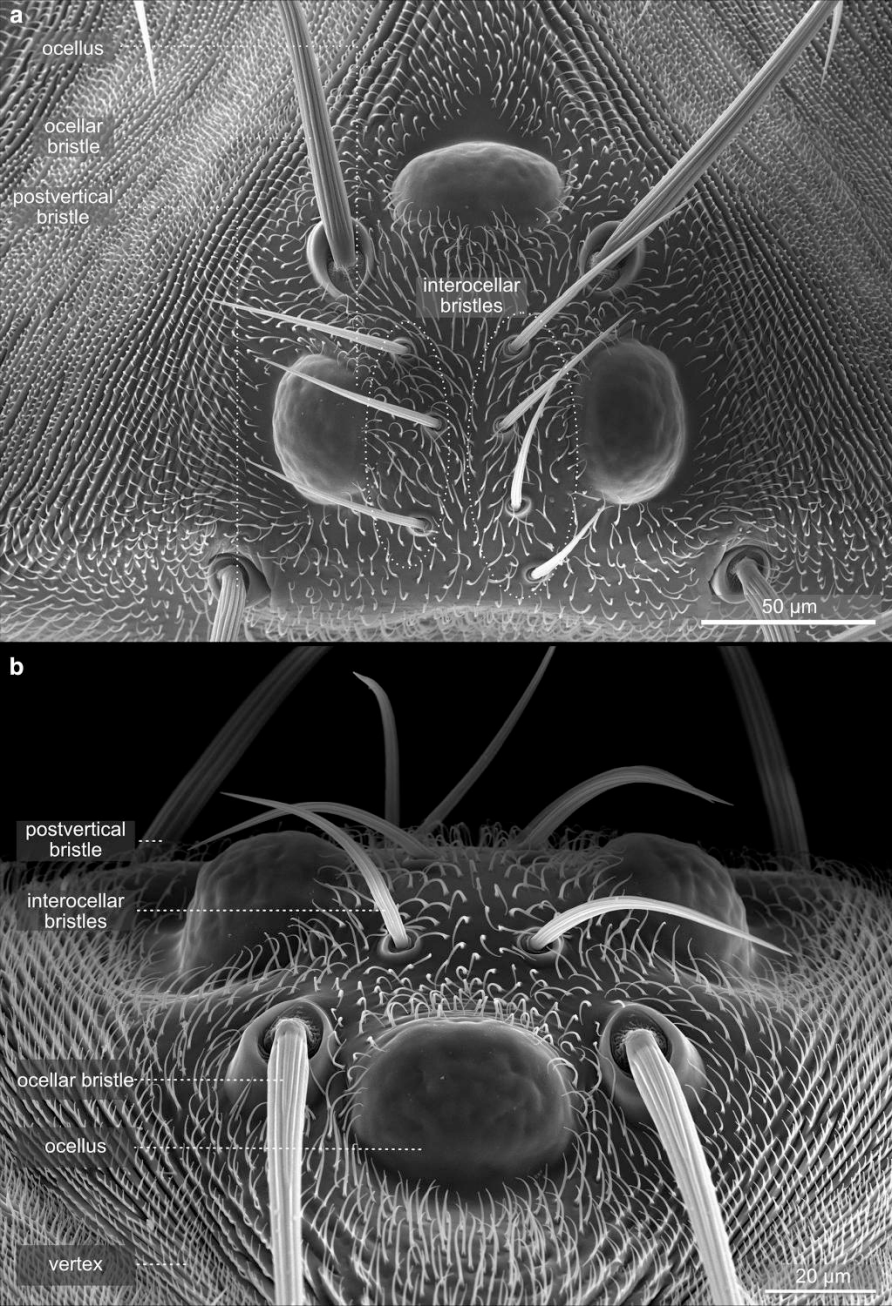
Ocelli eye—Overview. High magnification views of dorsal ocelli of the *Drosophila* head. These light-sensing organs are located in a group of three in the dorsal middle of the head. Ocelli serve as sensory organs and are involved in sensing the horizon, flight stabilization, and phototaxis. a) Slightly tilted view from posterior. The ocelli are surrounded by postvertical, ocellar, and interocellar bristles. b) Dorsal view of ocelli, illustrating the localization of bristles. The image also demonstrates the organization of bristles in vertices, indicating the planar polarization of cells.

**Fig. 19.**
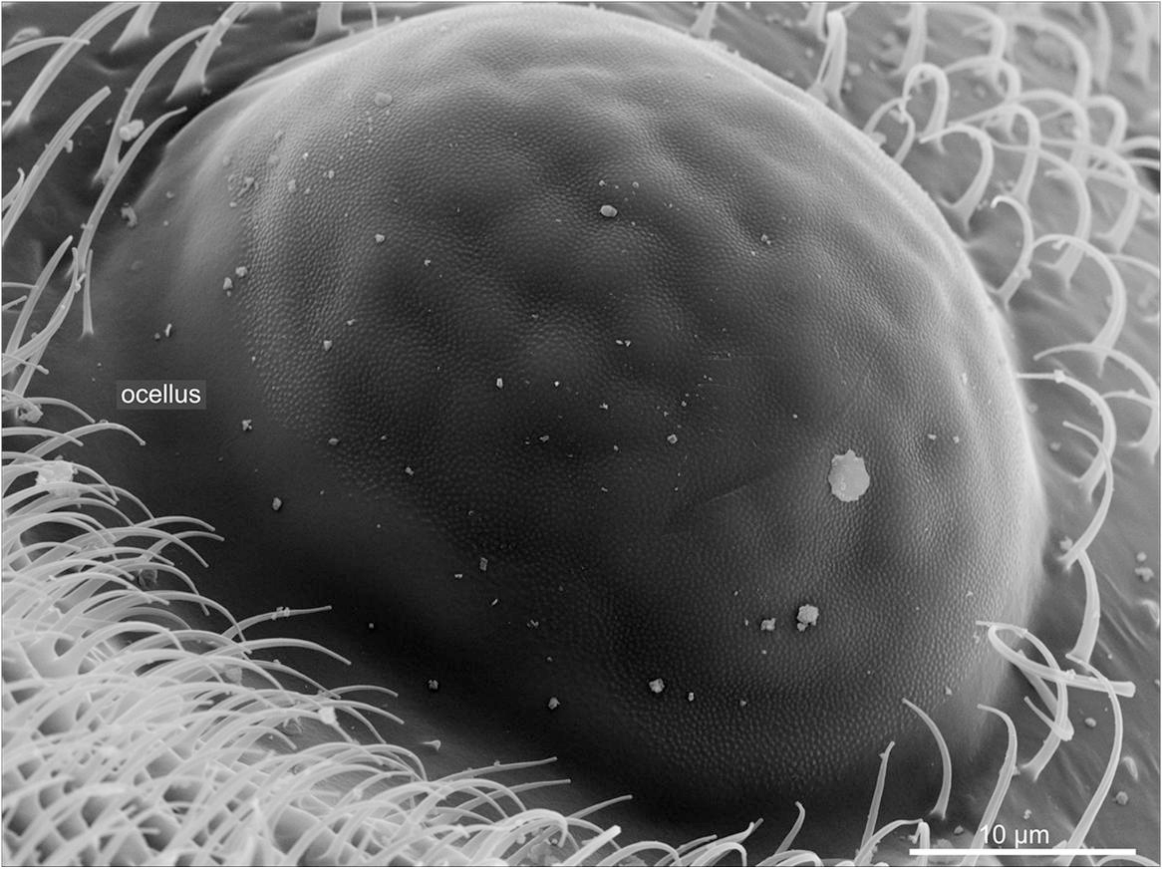
Ocellus—detail. High magnification of a single dorsal ocellus. Compared with the compound eye's complex organization, the ocellus's surface appears rather smooth. The cornea forms a single lens through which light is collected and projected to photoreceptors.

In addition to the compound eyes, the head bears small light-sensing organs at its medial-dorsal side—the ocelli (Figs. 18 and 19). In flies, there is always a triad of ocelli, a median one facing the anterior and a pair of ocelli facing the posterior (Fig. 18). The ocelli are situated in a distinct area called the interocellar cuticle, which also harbors a specific set of 6 to 8 interocellar bristles, which are located between the ocelli. The area is surrounded by 2 large ocellar and 2 postvertical bristles (Fig. 18). An ocellus uses a single lens, built by the cornea, to collect light and project it onto a layer of photoreceptors (Fig. 19). The ocelli contribute to various behaviors, including phototaxis, flight stabilization, entrainment of the circadian clock, color choice, and horizon sensing. For a detailed review of the morphology, neurobiology, and function of the visual system of the fly, we refer to (Jean-Guillaume and Kumar 2022) and the readings referred to within. The evolutionary ground pattern in insects is the presence of four ocelli. In Diptera, the single anterior ocellus may have arisen by a fusion of two.

#### The antennae and maxillary palps

**Fig. 20.**
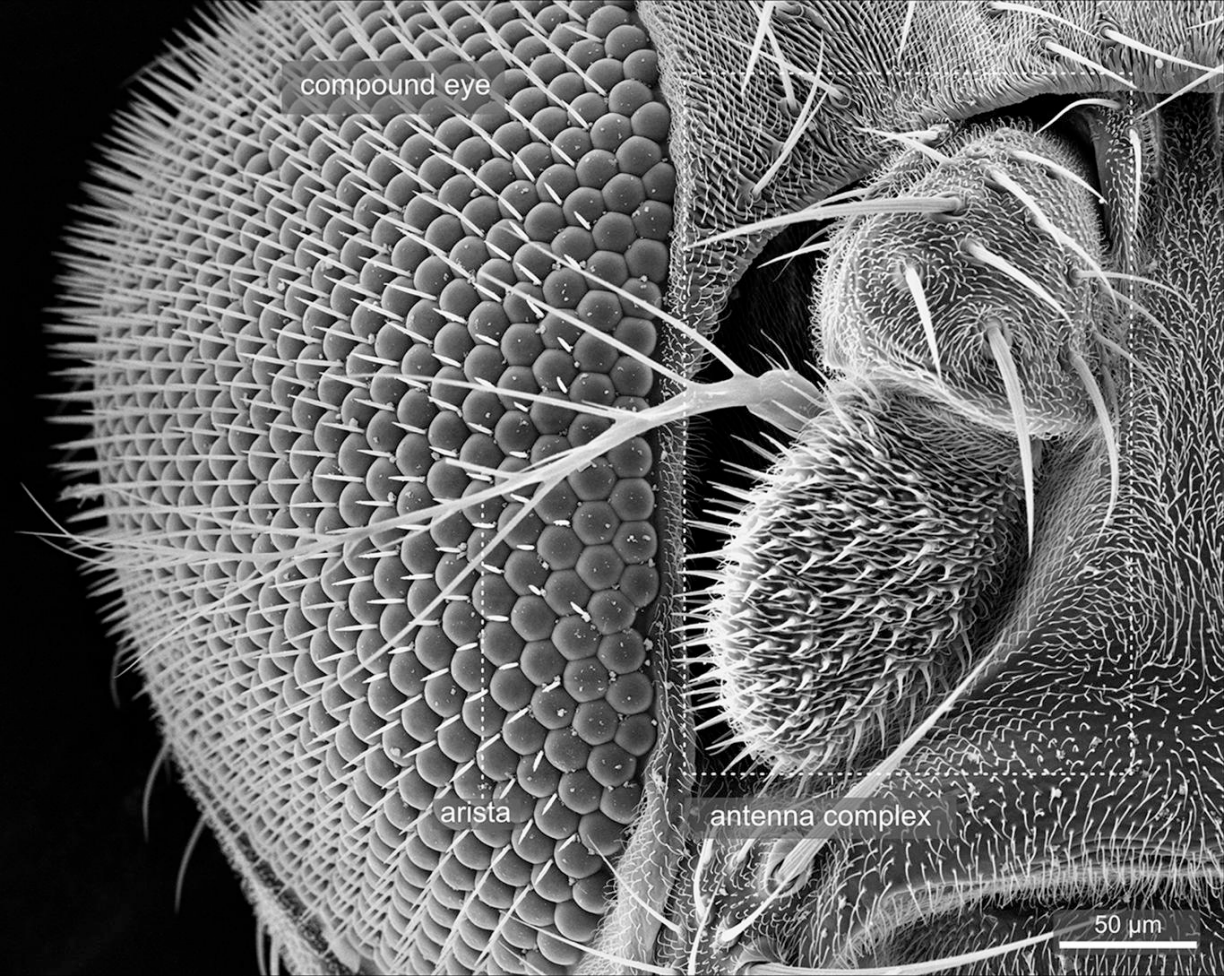
Head—eye—Antenna—overview. Frontal view of the head with the right eye, antenna, and arista in its normal position.

Adult flies possess two prominent olfactory organs—the antenna and the maxillary palps (Figs. 20^23^). From an anterior view, the paired antennal organs are located medially between the compound eyes, while the maxillary palps are situated at the retractable proboscis (Figs. 24–28). Together, they constitute the major olfactory sense organs of the fly.

**Fig. 21.**
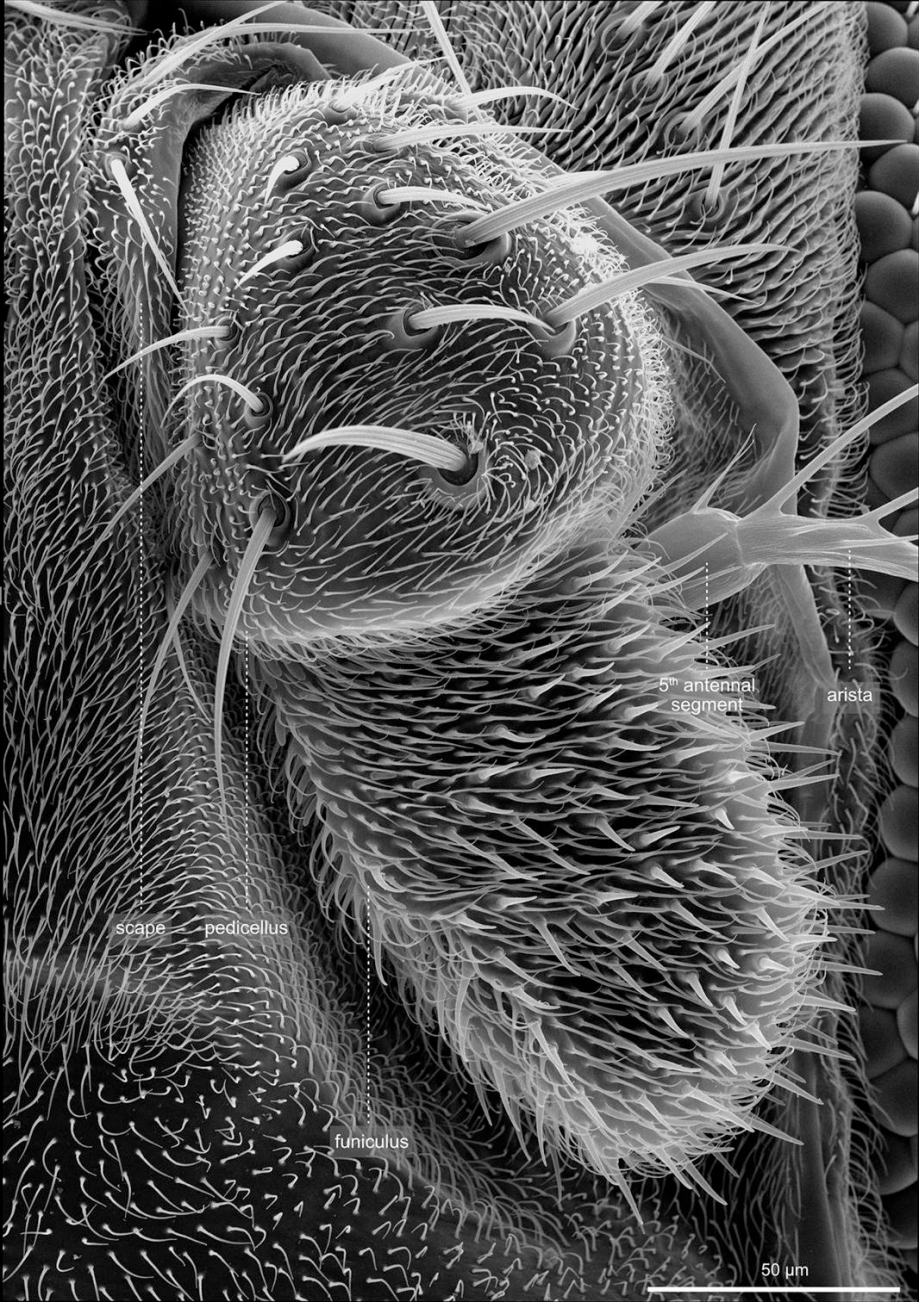
Head—Antenna. High magnification views of the antennae. The antennae emerge at the head's central anterior aspect and derive from the second head segment. Antennae comprise three main anatomical units: scape, pedicel, and the multijointed sensory flagellum, the funiculus. The scape forms a ring that holds the entire structure. The pedicel appears larger than the scape and has an irregular, swollen shape. It is prominently covered by large bristles. The funiculus appears more elongated and is covered with smaller bristles. The funiculi carry the aristae, giant modified bristles that receive “sound.” Each funiculus also comprises a large variety of olfactory sensilla.

**Fig. 22.**
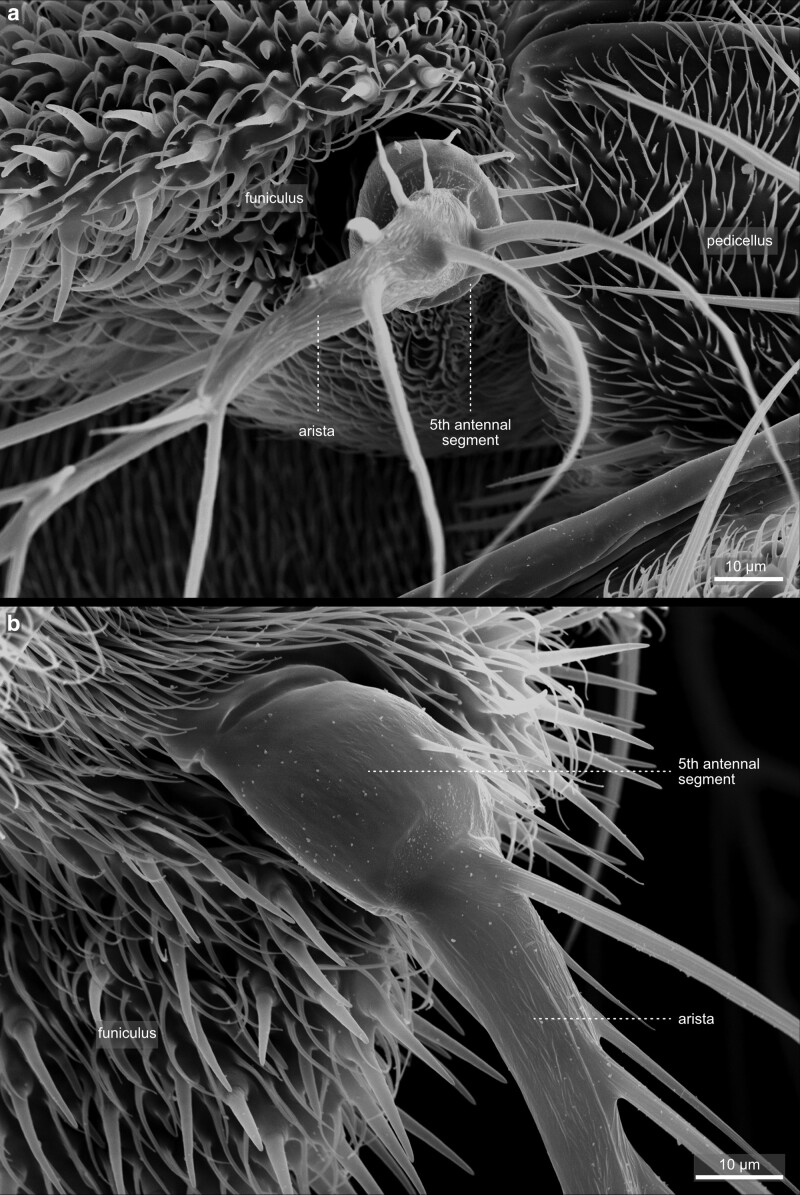
Head—Antenna—Arista—Base. a) The arista base from the top and b) from a lateral view. Note the branching of the arista sensory bristle a) and the central core area b) at the base. The core region is usually around 300 µm long and 10–20 µm in diameter (Foelix *et al*. 1989).

**Fig. 23.**
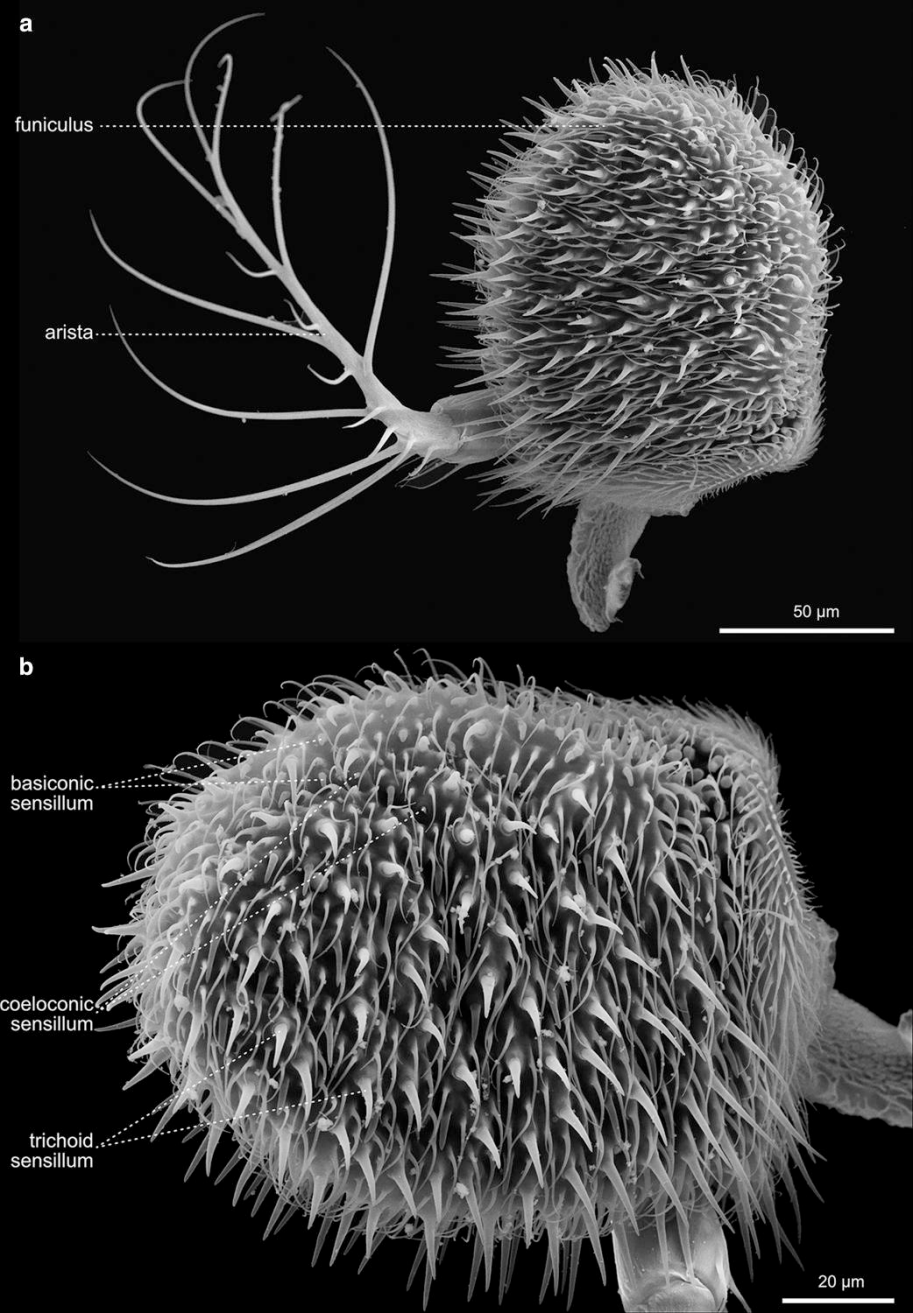
Head—Antenna—Arista and Funiculus. The funiculus belongs to the primary olfactory sense organs of the fly. The female funiculus is slightly longer than the funiculus of males. a) A dissected funiculus with olfactory sensilla and the left, highly branched arista. b) Higher magnification funiculus with various types of sensilla.

**Fig. 24.**
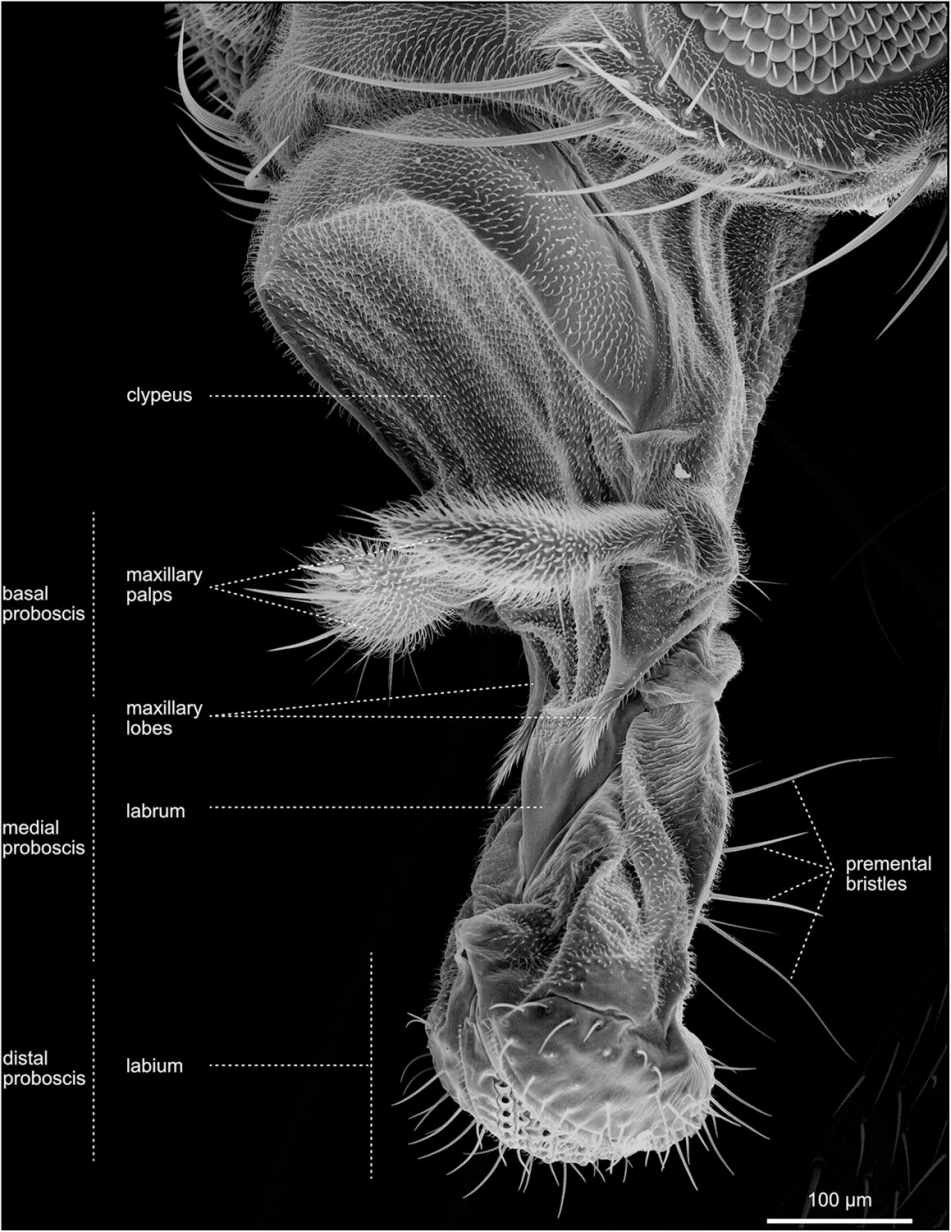
Head—Antenna—Proboscis—extended. Evaginated proboscis, containing, from its base, the clypeus, maxillary palpi (reduced in Diptera), maxillary lobes, labrum, and distal labium, consisting of two swollen labial palpi. The proboscis is moved by a complex array of muscles, allowing evagination and retraction of the entire structure and feeding by licking food from the substrate surface.

**Fig. 25.**
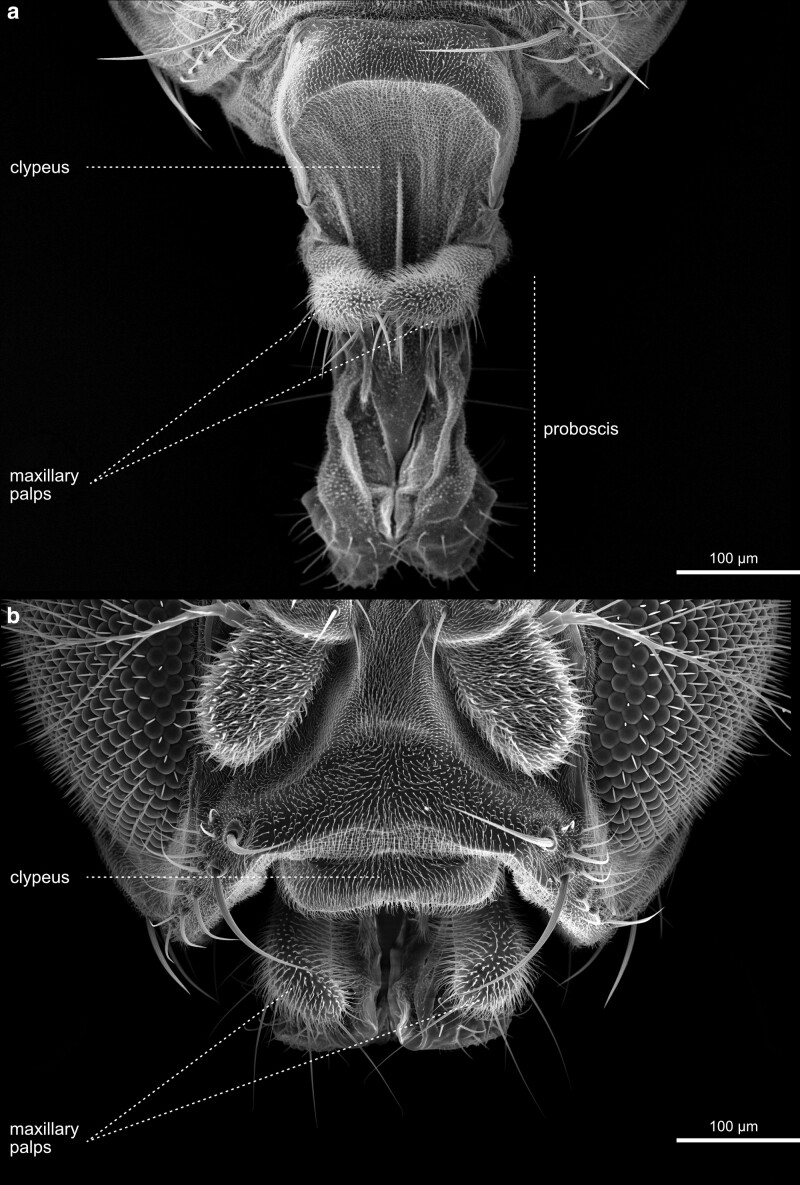
Head—Antenna—Proboscis—extended–retracted. Frontal view. Head with the extended a) and retracted b) proboscis.

**Fig. 26.**
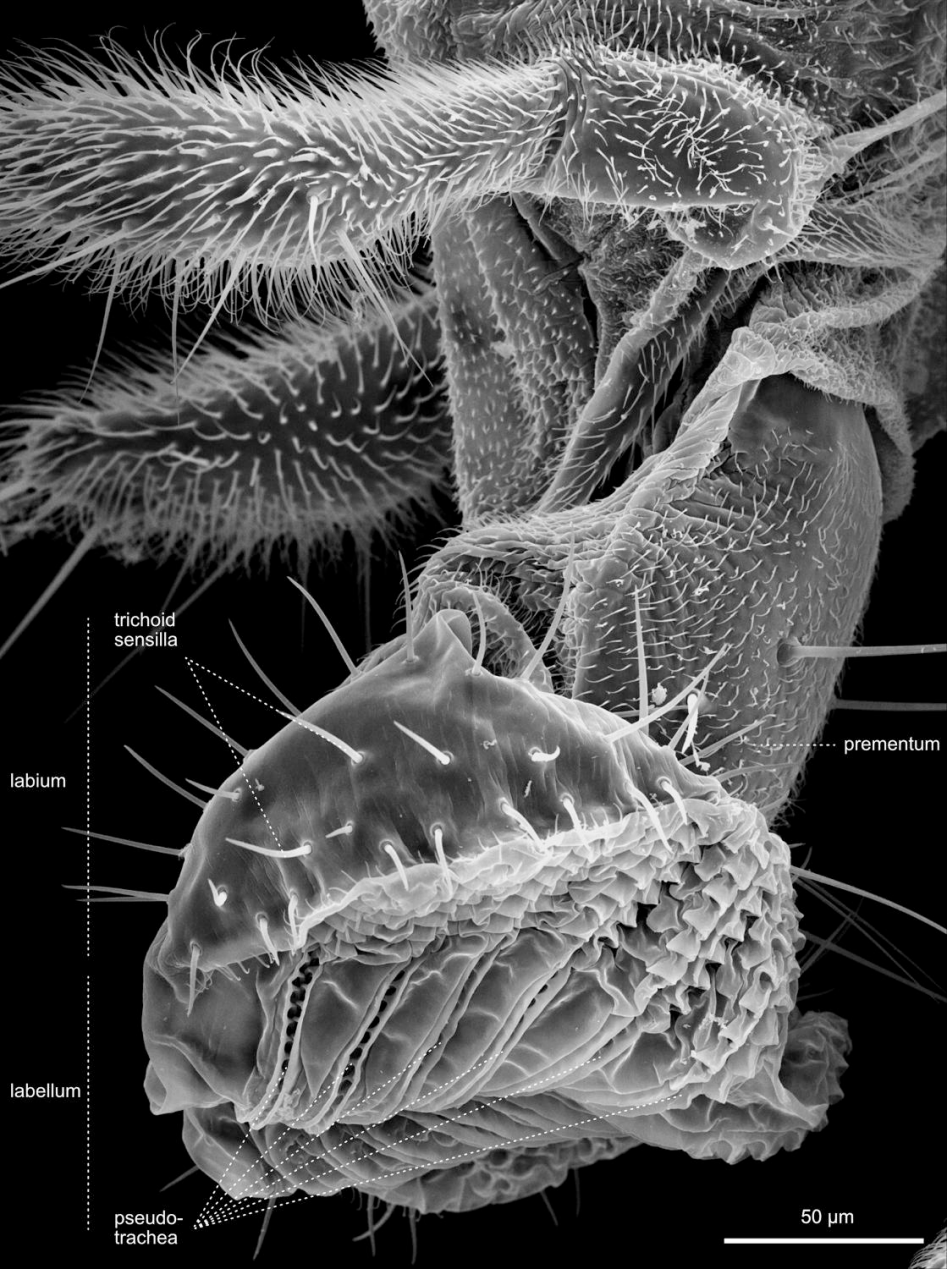
Head—Labrum and Labellum. High magnification view of the labrum, the labellum, and the maxillary palpi. Each labial palp contains six major grooves called pseudotrachea.

**Fig. 27.**
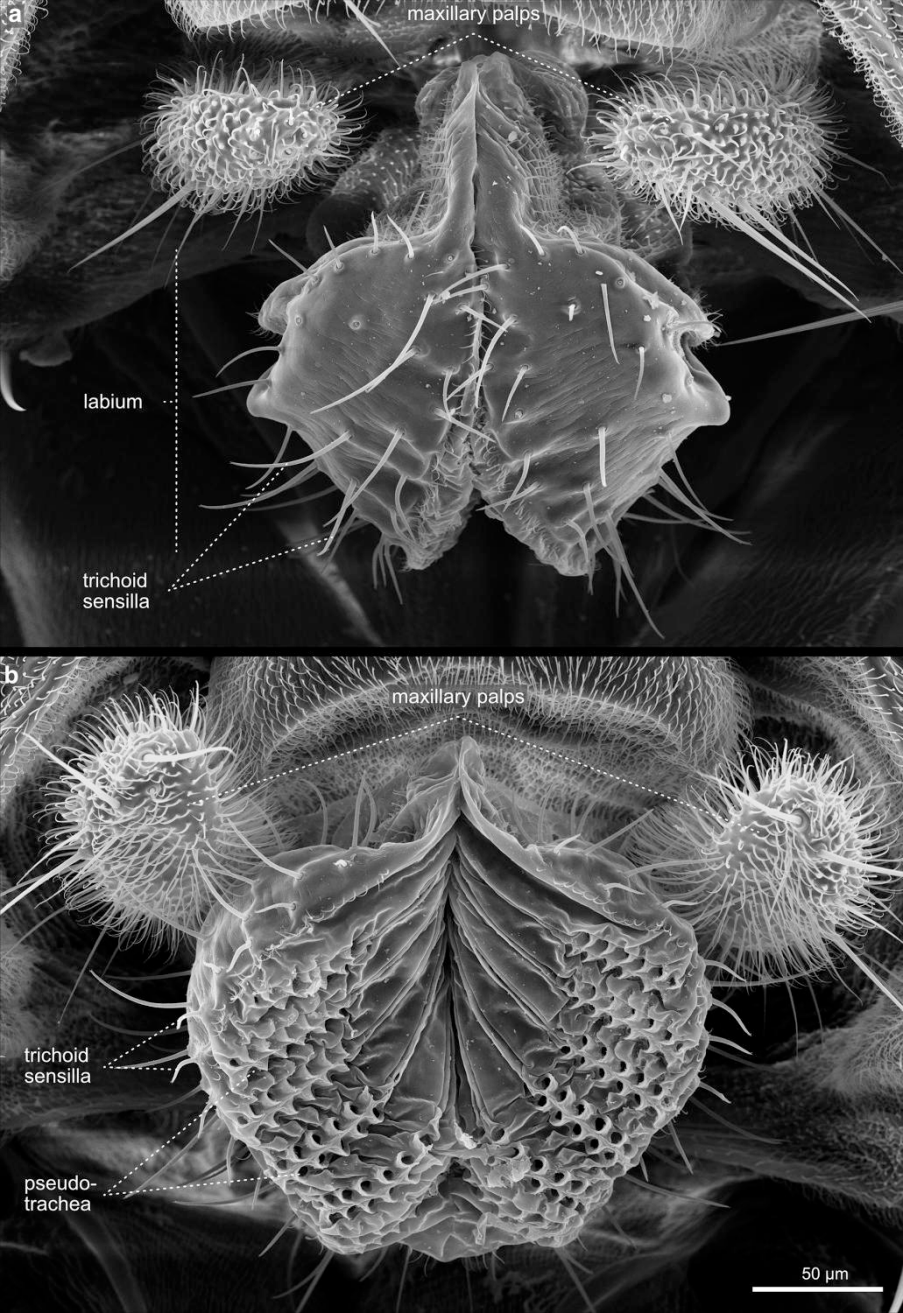
Head—Labellum—closed and open. a) Frontal view of invaginated mouthparts, with maxillary palpi visible. b) High magnification ventral view of the labium with clearly distinguishable labial palpi. These membranous grooves, called pseudotrachea, act as sponges, allowing liquid and food particles to uptake from the substrate.

**Fig. 28.**
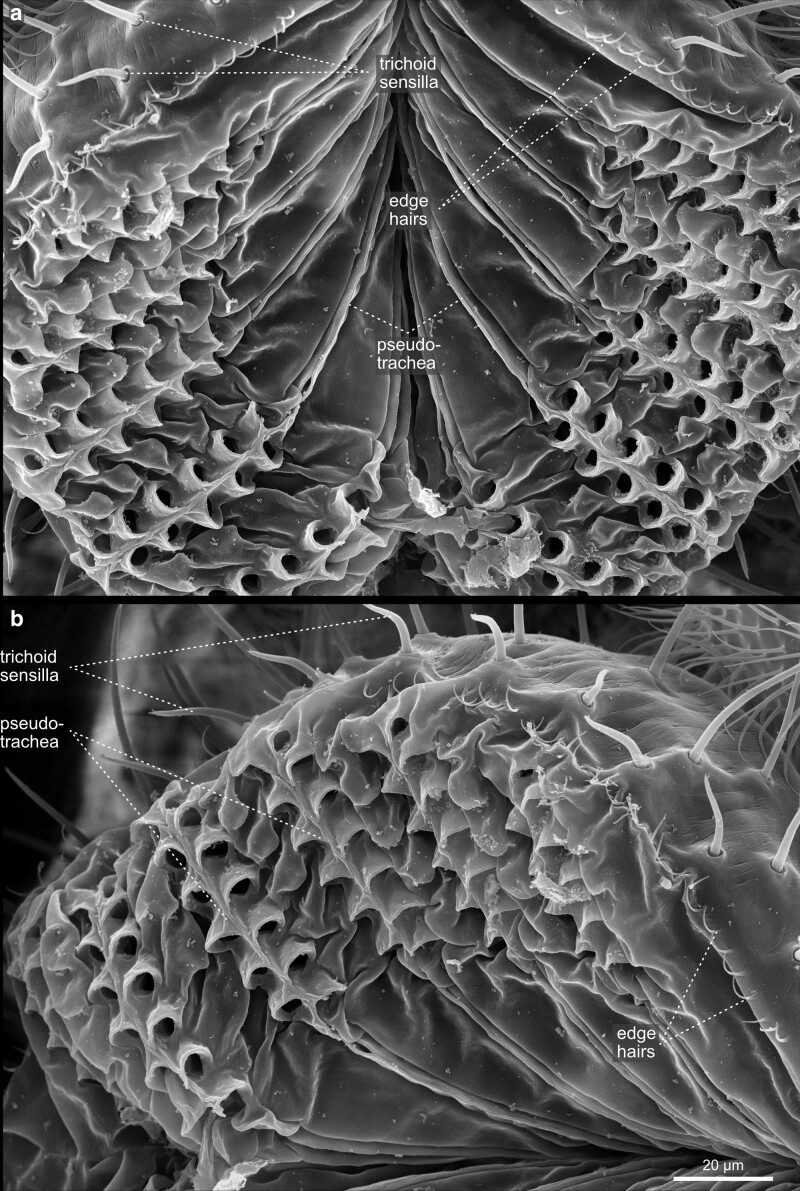
Head—Labellum—open—close-up. The images show the labellum at higher magnification from a a) frontal and b) lateral view.

The antenna exhibits a segmental organization (Foelix *et al*. 1989). The comparatively small first segment (scape) attaches to the head capsule (Fig. 21). It is followed by a large second segment (pedicellus), exhibiting a prominent, stereotyped bristle pattern (Figs. 21 and 23). The third antennal segment (funiculus) constitutes the actual olfactory sensory unit of the antenna. It harbors about 500 funicular sensory bristles for olfactory reception, grouped into three morphologically distinct classes—basiconic sensilla, trichoid sensilla, and coeloconic sensilla [Stocker 1994; Carlson 1996; Shanbhag *et al*. 2000 (Fig. 23)]. Basiconic sensilla appear club-shaped, and their surface is characterized by a dense network of tiny pores penetrating the cuticle. On the other hand, coeloconic sensilla are much shorter and exhibit a pointed end (Fig. 23b). On the surface of the cuticle, they exhibit regular longitudinal groves along the sensillum instead of pores (Fig. 22). Lastly, trichoid sensilla, the largest of the three classes, have a similarly pointed appearance as coeloconic sensilla. However, they arise from a relatively thick basal socket and harbor tiny pores along each sensillum (Fig. 22).

The funiculus connects to two more, smaller antennal segments (segments 4 and 5), which further connect to the prominent branched arista at the antenna's most distal tip (Figs. 22 and 23). The arista detects air movements and thus responds to sound by interacting with vibrating air particles. The arista is used to “hear” the courtship (males) and copulating songs (females), the sound produced by the vibration of the wings. The arista consists of three parts—a basal segment close to the funiculus, a cylindrical connective segment, and a highly branched, feathered shaft (Fig. 23). Together with the funiculus, the arista can function as a sound receiver, transforming sound into a rotary movement and thus transducing it to sensory input (Göpfert and Robert 2002).

The maxillary palps are a pair of organs located at the proximal portion of the proboscis (Figs. 24–26) and constitute the second olfactory sense organ situated at the head of the fly. They can easily be overlooked when the proboscis is retracted (Fig. 25). Maxillary palps exhibit a different morphology than the antenna and appear unsegmented. Furthermore, they harbor only 2 types of sensory sensilla, basiconic and trichoid sensilla, which have a similar morphology to their antennal siblings.

#### The proboscis

The retractable proboscis, or the mouthparts of the fly, constitutes the animal's feeding organ (Figs. 24–27). In principle, the proboscis constitutes a tube-like extension of the head, which allows food uptake from the substrate and harbors various sensory sensilla. From proximal to distal, the proboscis can be subdivided into a basal, medial, and distal part [Fig. 24 (Ferris 1950; Wildermuth and Hadorn 1965)]. The connection between the head and proboscis is formed by the clypeus, a large sclerotized joint that allows the backfolding of the entire feeding apparatus (Figs. 24 and 25). The attachment of the maxillary palps characterizes the basal proboscis. The medial proboscis exhibits a naked cuticular shield (labrum) at its dorsal side and a larger sclerotized cuticle plate (prementum) on its ventral side (Figs. 24 and 26) (Wildermuth and Hadorn 1965). As in most areas of the head, the prementum is covered by many microtrichia and holds eight to ten large bristles. Since we could not find a precise nomenclature for those bristles, we suggest referring to them as “premental bristles.” The most distal part of the proboscis descends into the actual mouth-opening structure of the fly, the labium. The labium is divided into a sclerotized outer surface, covered by trichoid sensilla [Figs. 26–28 (Struhl 1981)]. The inner face of the labium, the labellum, consists of two opposable cushions, the labial palps, which are covered and not visible from the outside when the labium is closed (Fig. 27).

Each labial palp exhibits six channels (pseudotracheae), which allow the flow and subsequent uptake of liquid food and mucus (Fig. 28). Morphologically, the inner and the outer surfaces of the labial palps are separated by a single row of bristles called the edge hairs (Fig. 28). In addition to the edge hairs, the labial palps harbor distinct rows of gustatory sensory sensilla (trichoid sensilla) involved in the taste reception of the fly (Figs. 26–28). These sensilla have a very conserved arrangement throughout the labellum, even between individuals, while each sensillum protrudes from a single cuticular socket. Another set of gustatory sensilla is present between the pseudotrachea of the labial palps. However, since they are primarily buried within the grooves of these structures, they are barely visible in SEM images. Together, these sensilla form the major taste-processing structures at the head of the fly (Falk *et al*. 1976; Nayak and Singh 1983; Stocker 1994; Ebbs and Amrein 2007).

## The thorax

**Fig. 29.**
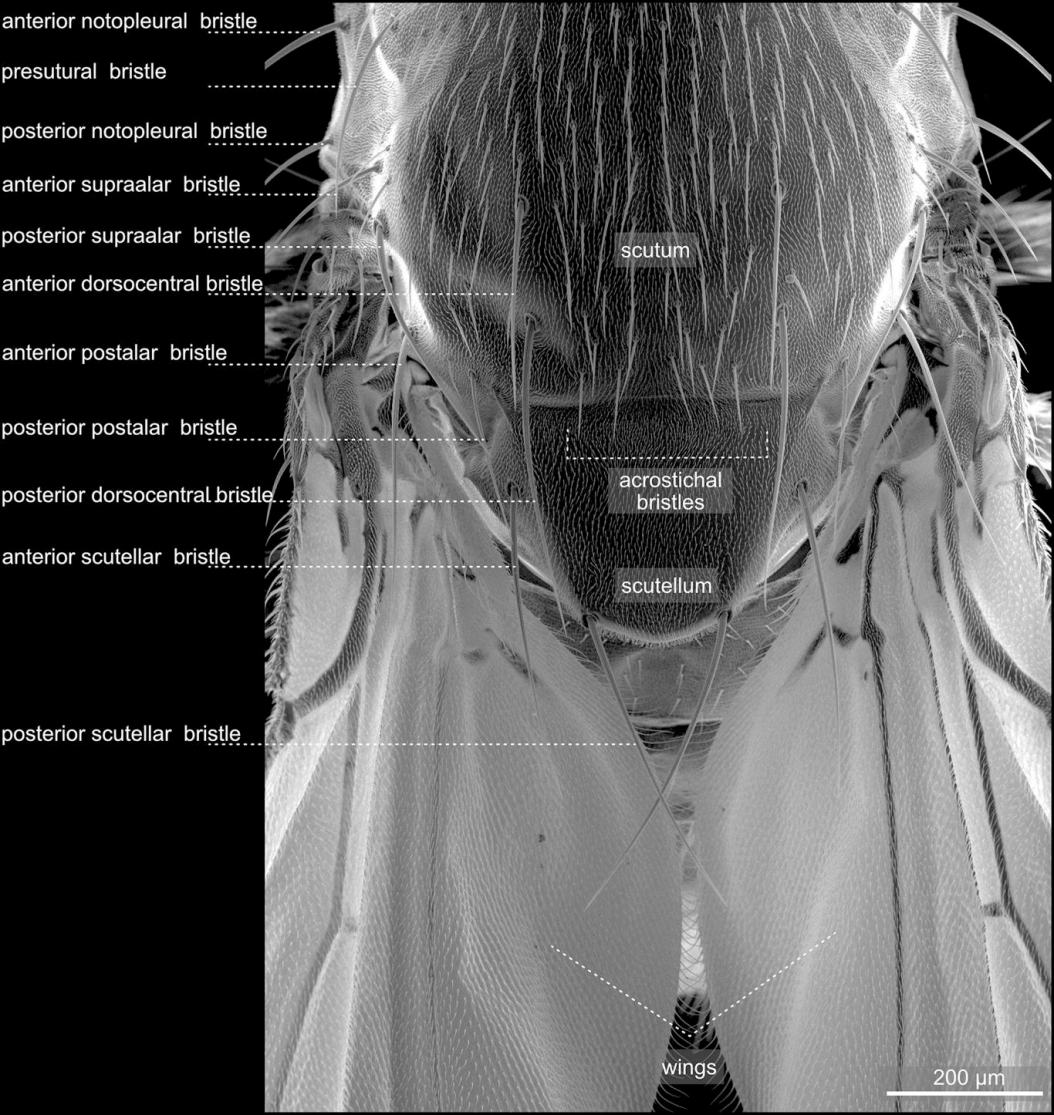
Thorax—abdomen transition—dorsal. Dorsal view of the scutum, scutellum, and wings. The scutum forms the anterior part of the thorax and is a critical component in flight muscle attachment. The scutellum, located posteriorly, contributes to balance during flight. These thoracic segments are crucial for the fly's flight capabilities and coordinated movement. The bristles located at the posterior end of the scutellum in *Drosophila* serve as mechanosensory structures. These specialized sensilla detect mechanical stimuli and provide the fly with crucial information about its environment, aiding in flight control and navigation. *Drosophila* is a relatively bristleless fly species. As Ferris (1950) pointed out, “a complete terminology of the bristles in the more “hairy” flies is much more elaborate than that here employed” for *D. melanogaster*. For a detailed description of the thoracic bristles, we refer to the studies by Demerec (1950), Ferris (1950), and Fabian *et al*. (2016).

**Fig. 30.**
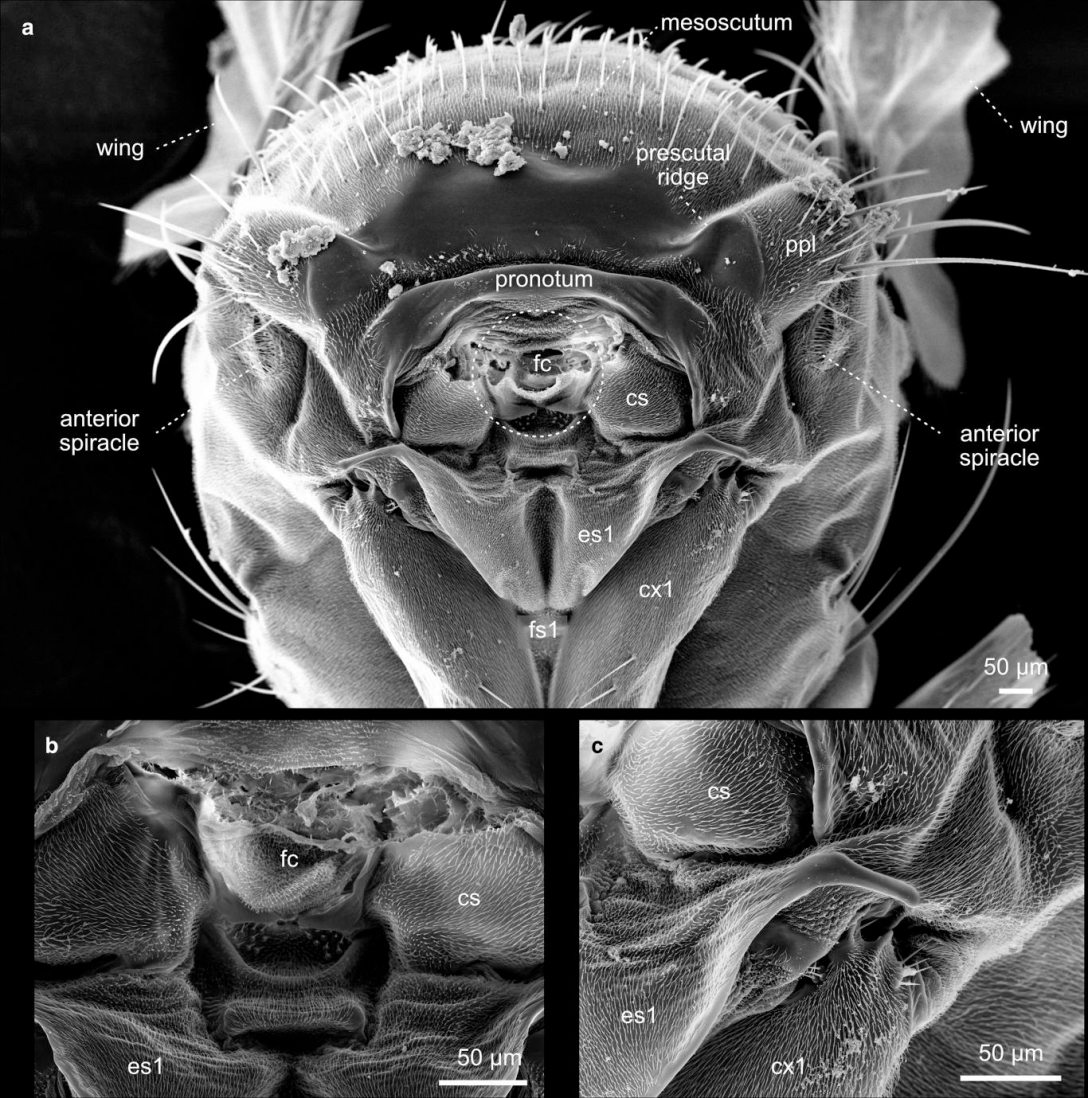
Thorax—frontal view. a) Frontal view of a thorax detached from the head. b) and c) higher magnifications. Cs = cervical sclerite, cx1 = procoxa, es1 = proepisternum, fc = foramen cervicale, fs1 = profurcasternum, ppl = postpronotal lobe.

**Fig. 31.**
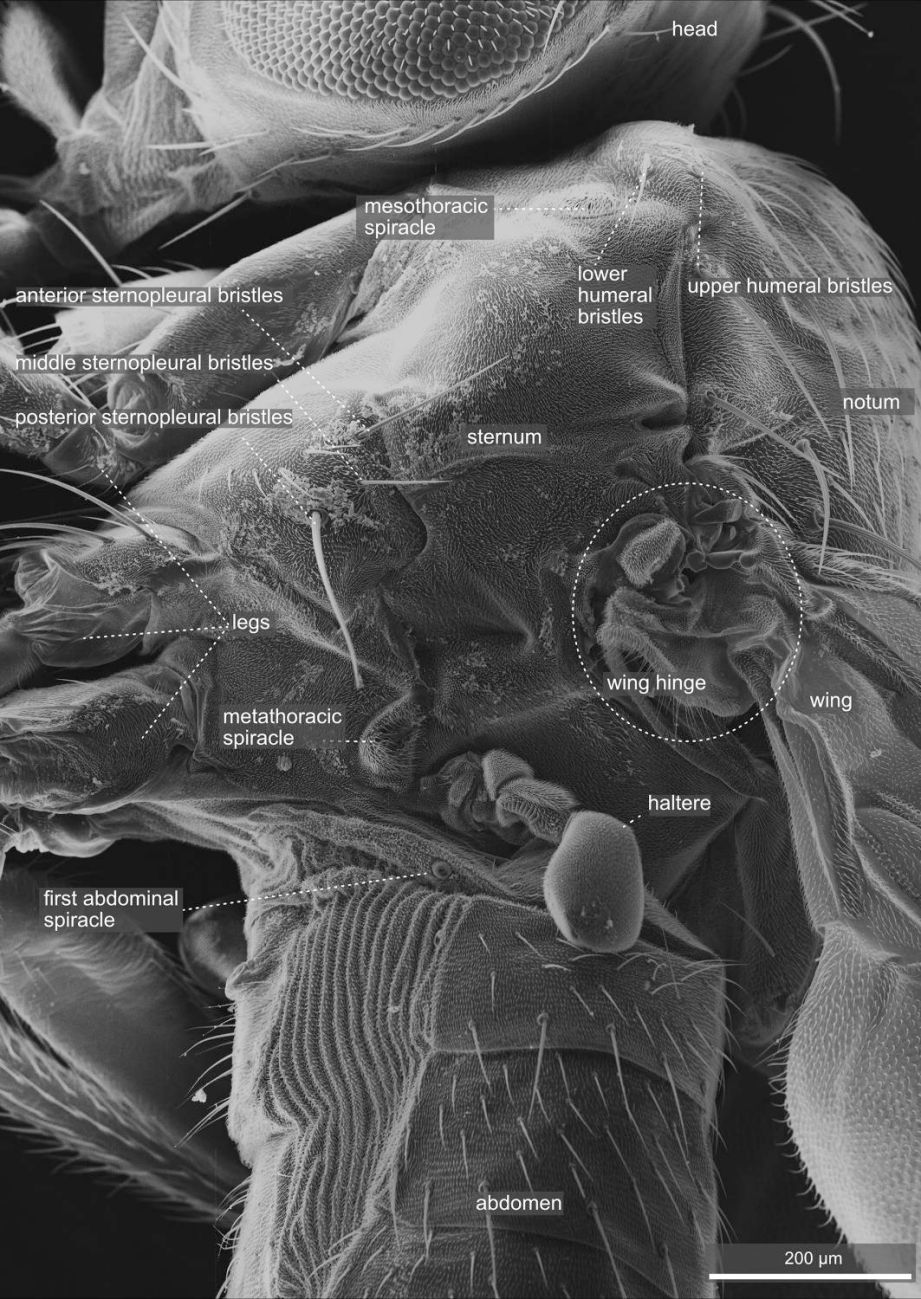
Thorax—lateral. Lateral view of the *D. melanogaster* thorax and anterior part of the abdomen. The anterior is up and dorsal to the right. Cells on the surface are yeast remains from the food vial. The image illustrates the anatomy of the wing hinge and the haltere insertion. The haltere, morphologically speaking, is the equivalent of a metathoracic wing, a gyroscopic sensor for maintaining balance and coordinating flight maneuvers. In addition, the upper and lower humeral bristles next to the head can be observed.

**Fig. 32.**
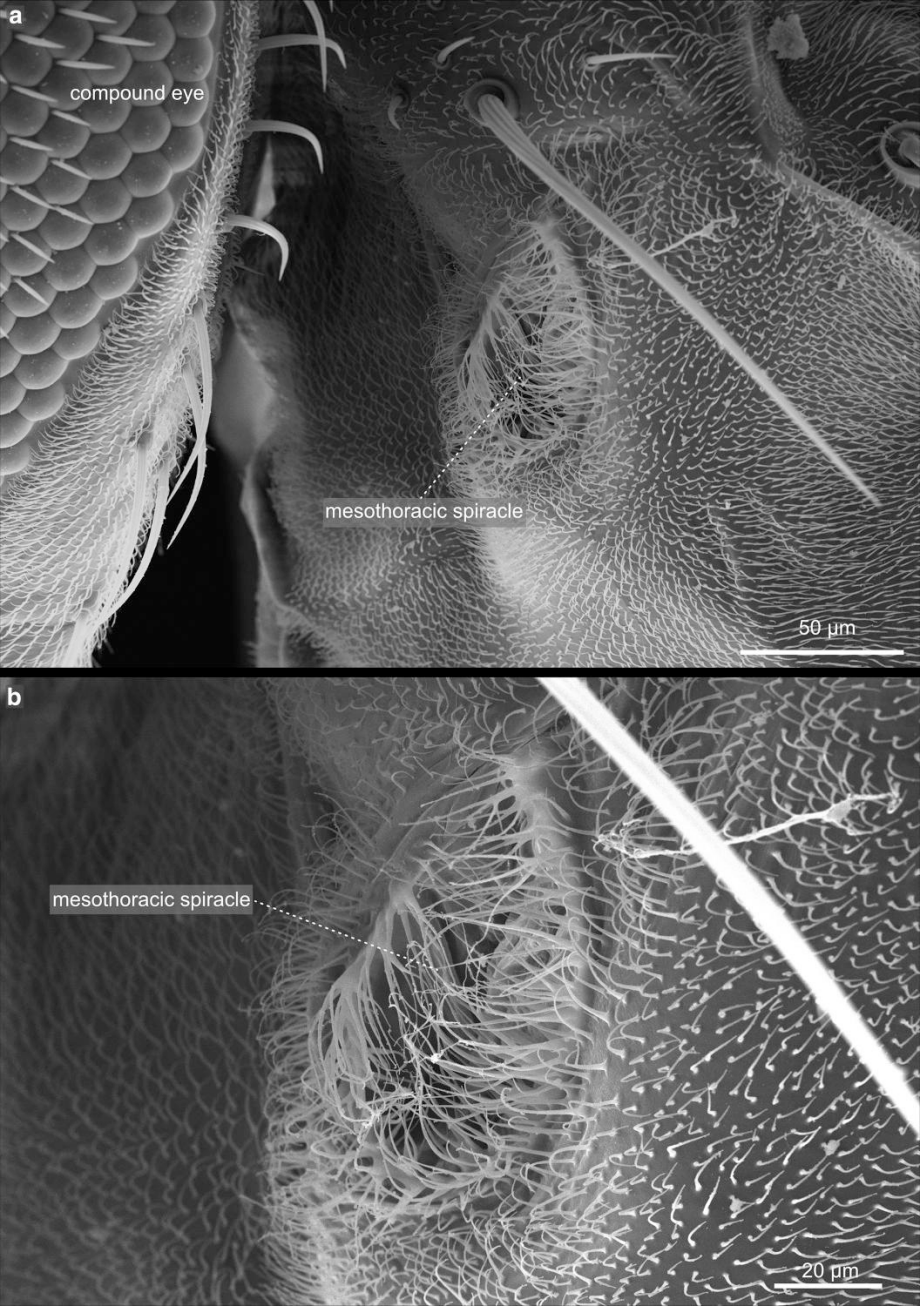
Thorax—Mesothorax—spiracles. a) Lateral view of the mesothorax with the tracheal opening (spiracle) visible. b) The mesothoracic spiracle at higher magnification. The relatively large aperture represents the entry to the tracheal system of the fly and is closed by a dense network of flexible bristles to prevent the entry of unwanted material, including parasites.

**Fig. 33.**
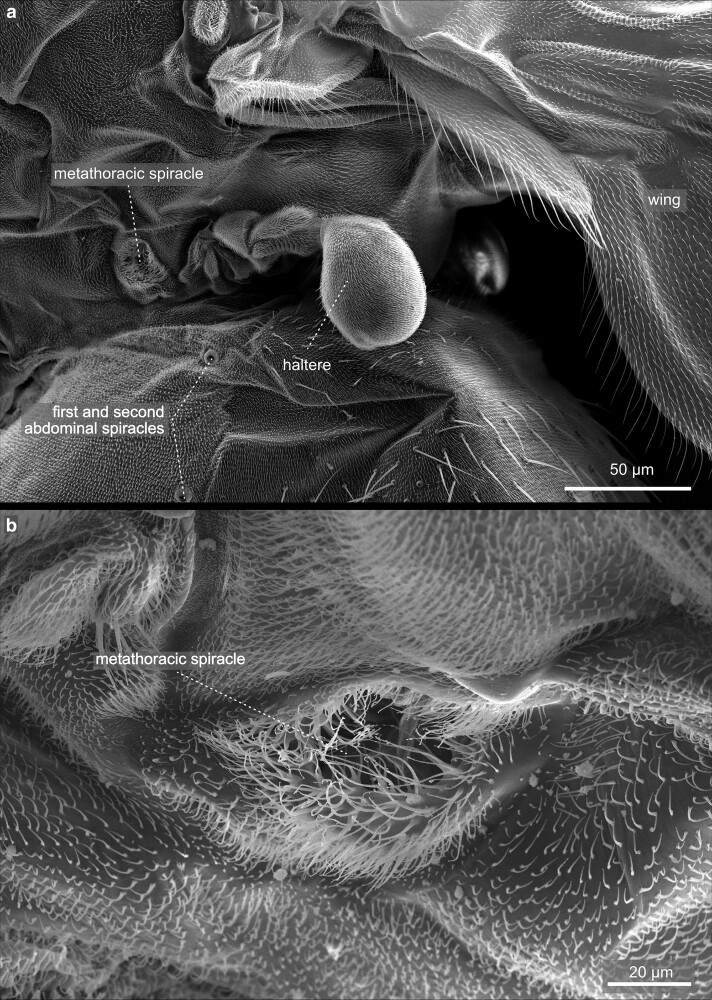
Thorax—Metathorax—Spiracles: a) Lateral view of the metathorax with the tracheal opening (spiracle) visible. The first 2 abdominal spiracles are also visible. b) The metathoracic spiracle at higher magnification. The meso- and the metathoracic spiracles are very similar in structure and size.

**Fig. 34.**
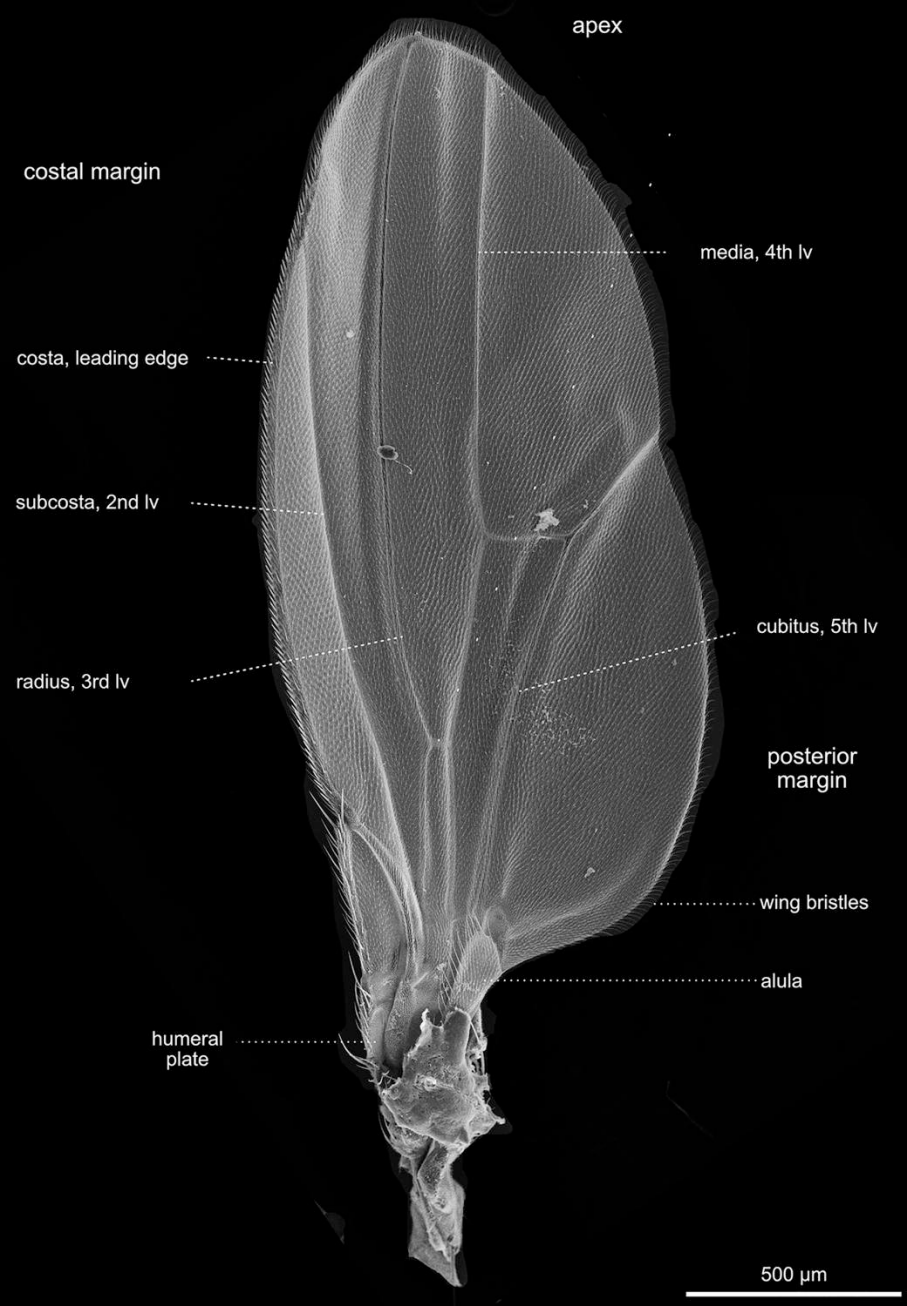
Thorax—wings—overview. Lateral view of the right wing with its intricate system of cells and veins. Within each of the prominent veins are a trachea and a nerve. The cavities of the veins are connected with the body cavity of the fly and are, therefore, filled with hemolymph. The veins serve as structural supports for the delicate wing membrane. These veins provide stability, strength, and shape to the wing, enabling the fly to generate lift and maneuver during flight. The wing venation pattern contributes to *Drosophila*'s remarkable flight capabilities, allowing for efficient movement through the air and successful navigation in its environment. Lv, longitudinal vein.

**Fig. 35.**
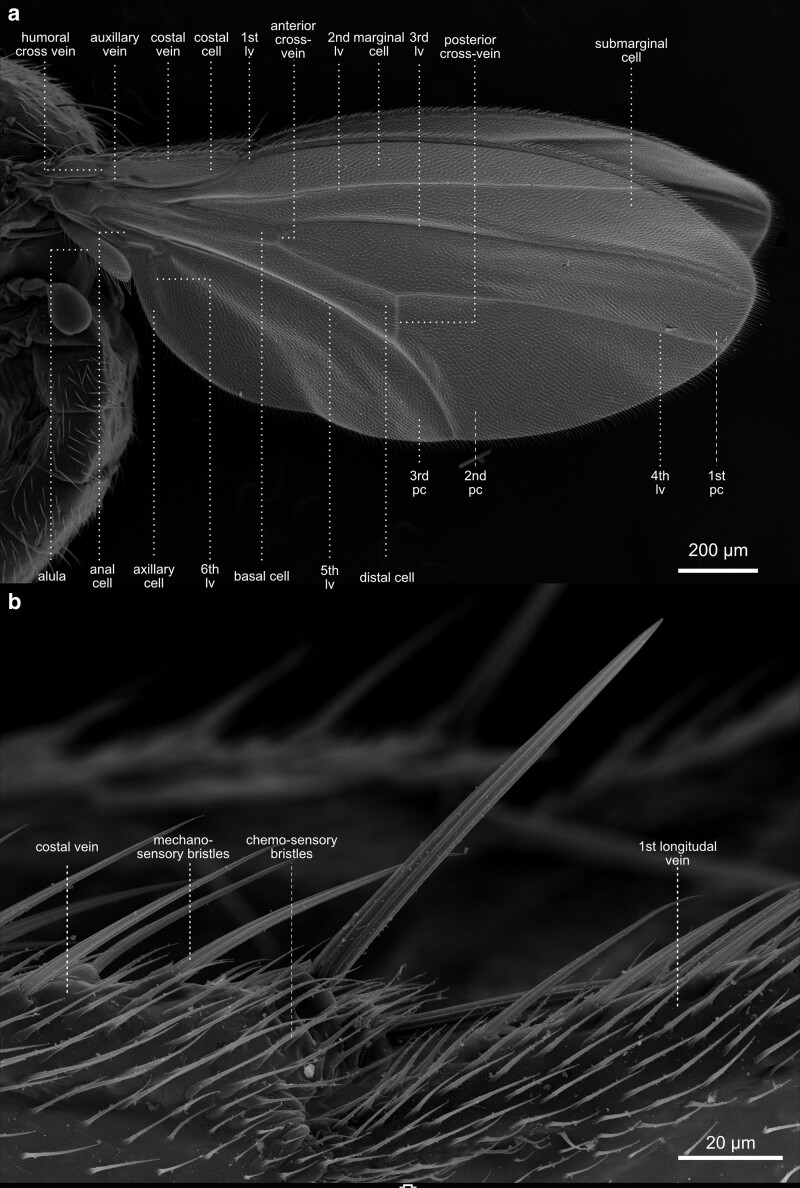
Thorax—wings—veins. a) Lateral overview of the different cells and veins of the wing. The cells make up the wing membrane itself, while veins are the rigid structures that support and reinforce the wing, forming a framework for its shape and function. b) Close-up of the cells and veins of the wing hinge. From the wing hinge, a network of veins radiates into the wing, forming a complex pattern. The costal cells are positioned along the leading edge of the wing and play a role in maintaining wing rigidity, aiding in lift generation during flight. The submarginal cells are near the wing's outer edge, contributing to wing flexibility and overall aerodynamics. Lv, longitudinal vein; Pc, posterior cross-vein.

**Fig. 36.**
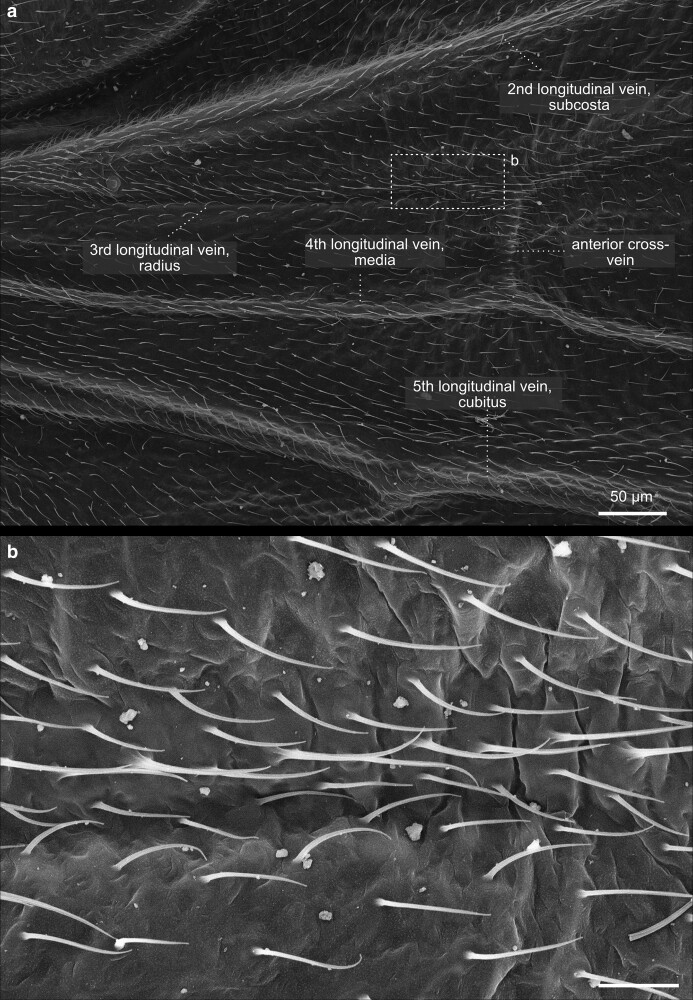
Thorax—wings—veins. a) Lateral close-up of the costal vein and the costal cells. b) The macrochaete on top of the costal vein are mechanosensory structures. They are specialized sensilla that detect mechanical stimuli and provide the fly with information about its wing movement and surrounding airflow dynamics. By sensing changes in air pressure and wing deformation during flight, these bristles contribute to the fly's abilities to navigate, maintain stability, and adjust its wing motions for efficient flight control.

**Fig. 37.**
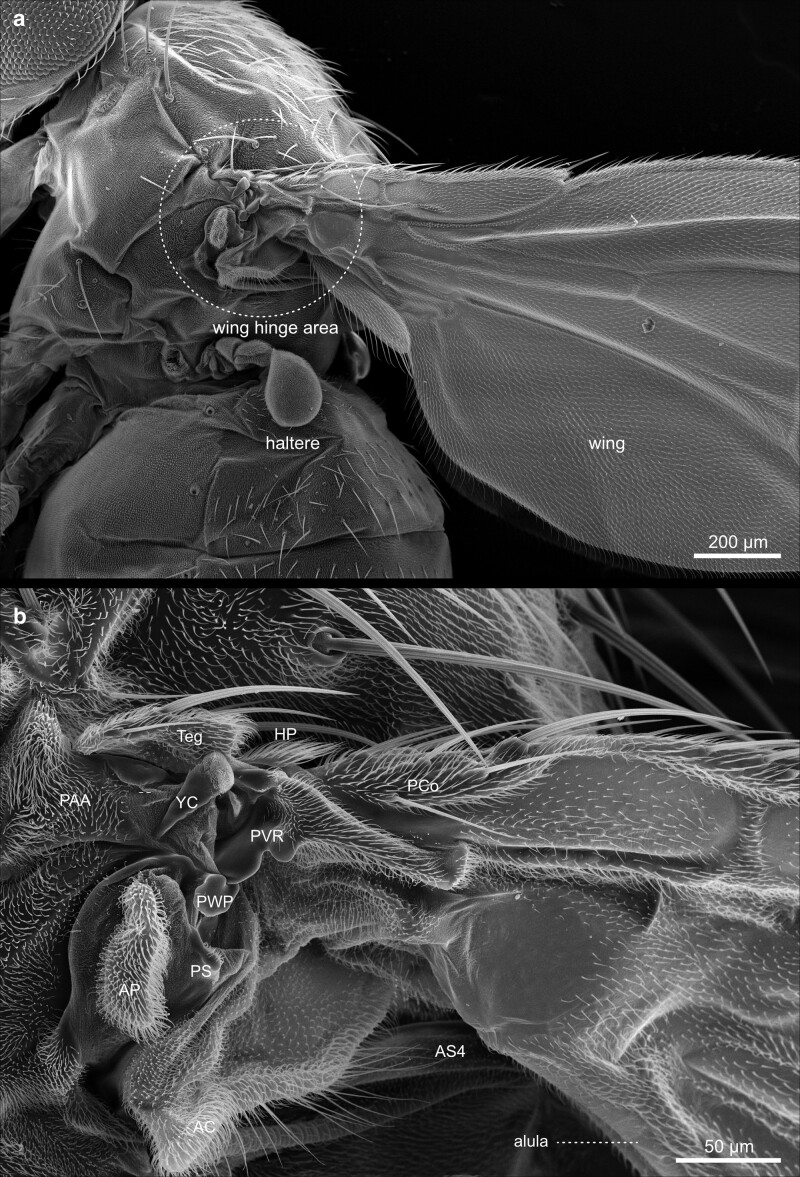
Thorax—wing hinge—details. a) Ventrolateral view of the wing hinge and its components in detail b). The wing hinge is a pivotal joint that enables controlled movement during flight. It allows the fly to generate lift, maneuver, and maintain stable flight patterns by precisely articulating the wings. AP, axillary pouch; AS4, fourth axillary sclerite; HP, humeral plate; PAA, prealar apophysis; PCo, proximal costa with bracted bristles; PS, pleural sclerite; PVR, proximal ventral radius; PWP, pleural wing process; Teg, tegula; YC, yellow club.

**Fig. 38.**
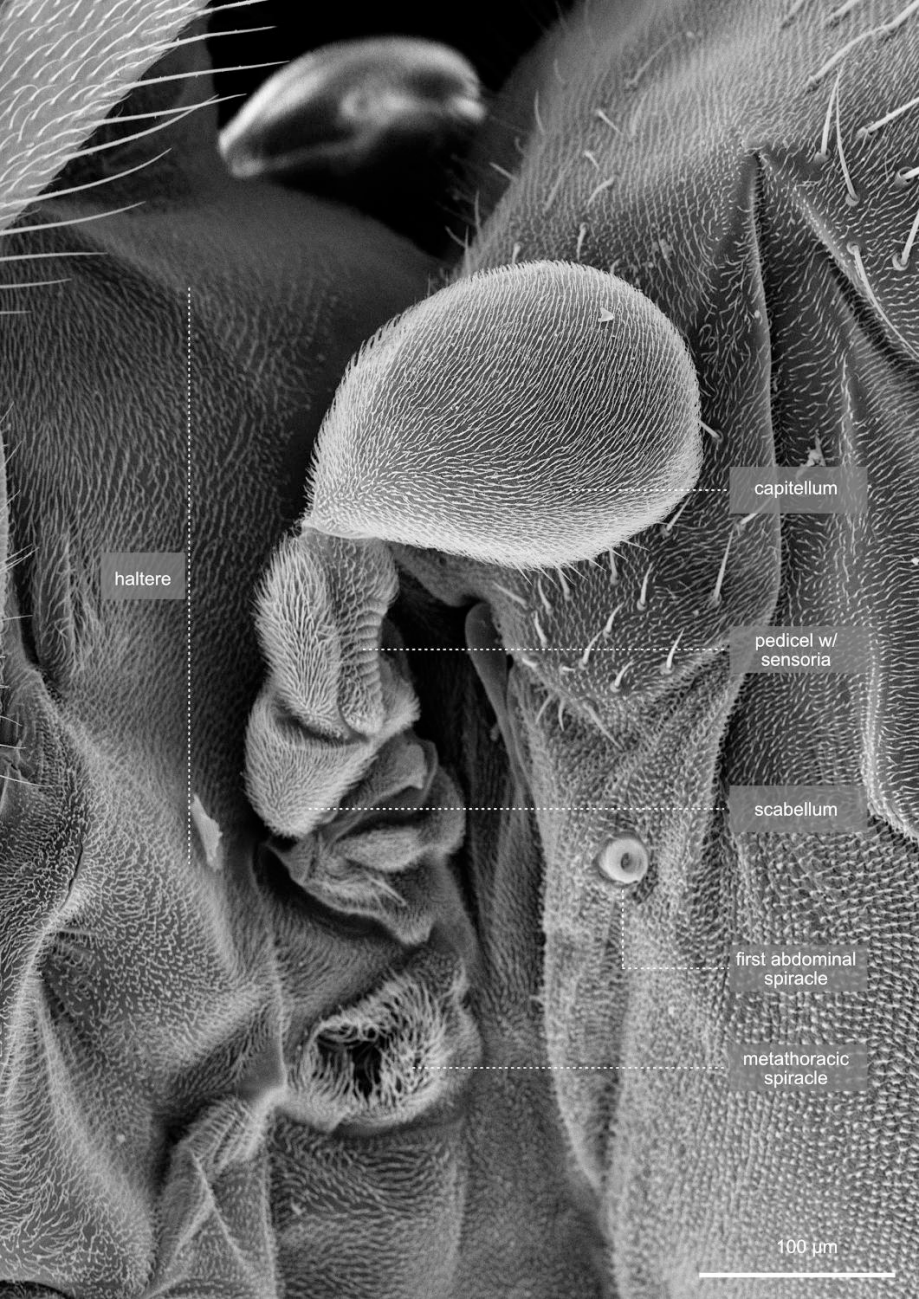
Thorax—halteres—overview. The metathorax is largely reduced in *Drosophila* and wings have been reduced to the halteres. Halteres are small, club-shaped structures that give *Drosophila* a remarkable sense of balance and orientation during flight. They act as gyroscopic sensors, aiding in mid-flight adjustments and maintaining stable flight patterns. The halteres on the metathorax are divided into 3 anatomical parts: the basal lobe (scabellum), a stalk (pedicullus), and a large apical end-knob (capitellum), all of which are visible.

**Fig. 39.**
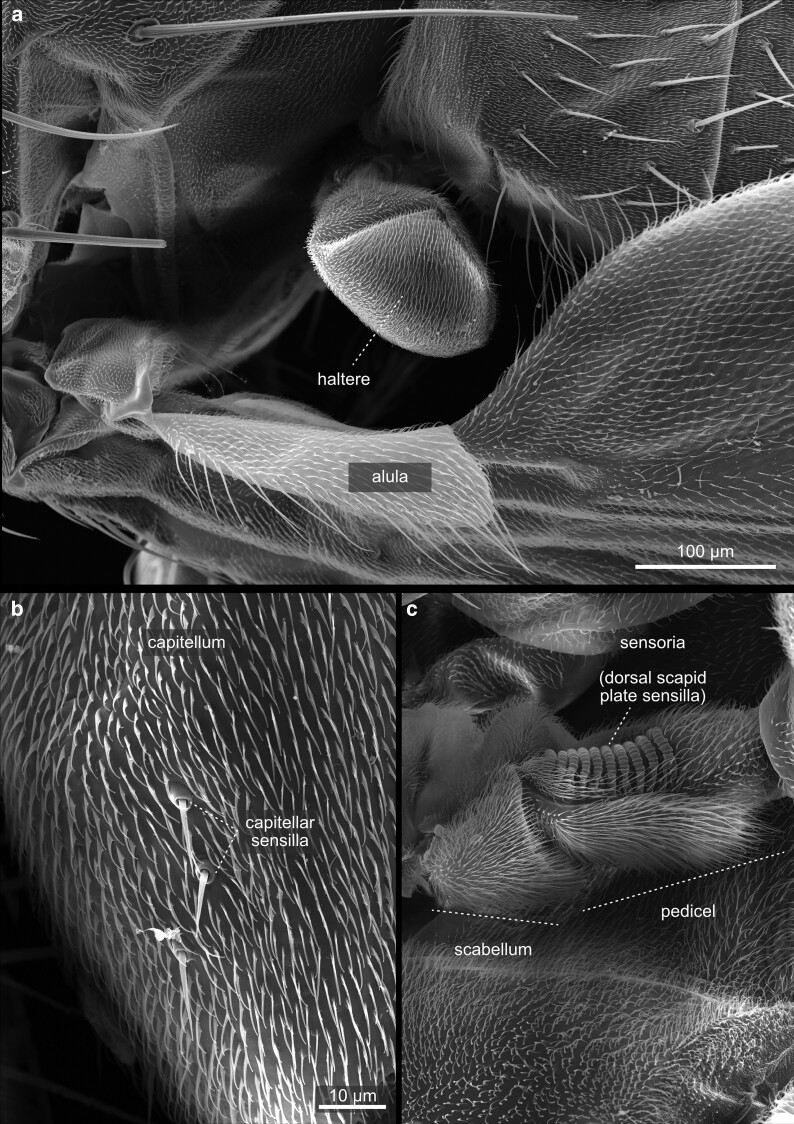
Thorax—halteres—sensilla. All 3 regions of the haltere host a variety of sensilla for sensing Coriolis forces, and proprioceptive forces. a) Dorsal side with Hicks papillae and a scapal plate with many individual sensilla. b) Hicks sensilla at higher magnification. c) Ventral side of the haltere with a second sensilla containing a scapal plate, and another group of Hicks papillae.

**Fig. 40.**
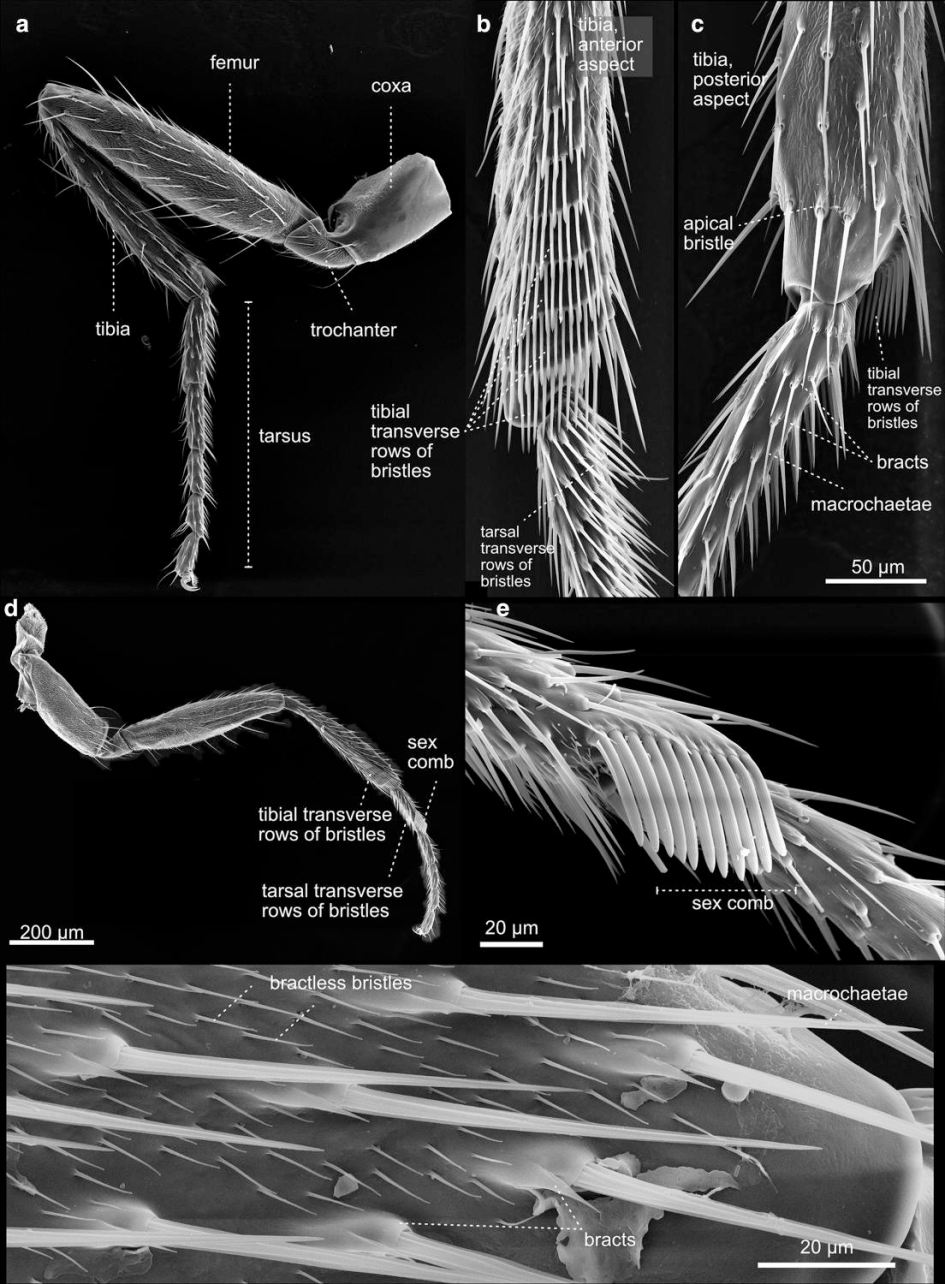
Fore leg—T1—female—male—details. a) Caudal aspect of a female left foreleg, overview. The leg is subdivided into the coxa, trochanter, femur, tibia, and the tarsus. Bristle anatomy and distribution differ in the anterior b) and c) posterior aspect of the leg. The transversal rows of bristles on the tibia and tarsus are a characteristic feature of the first leg. In *Drosophila*, the foreleg exhibits a sexual dimorphism. The male’s leg d) holds the metatarsal sex comb, displaying 11 bristles. e) The sex comb is one of the most rapidly evolving male-specific traits in *Drosophila*. The males employ their sex combs to grasp the abdomen and genitalia of females and to spread their wings before copulation. There are 11 sex-comb teeth, with slight variation among individuals and strains. The length of the comb, and thus the number of bristles, is species-specific. This makes the sex comb an attractive model for the study of sexual selection. f) Close-up of smaller tarsal bractless and larger bristles with bracts.

**Fig. 41.**
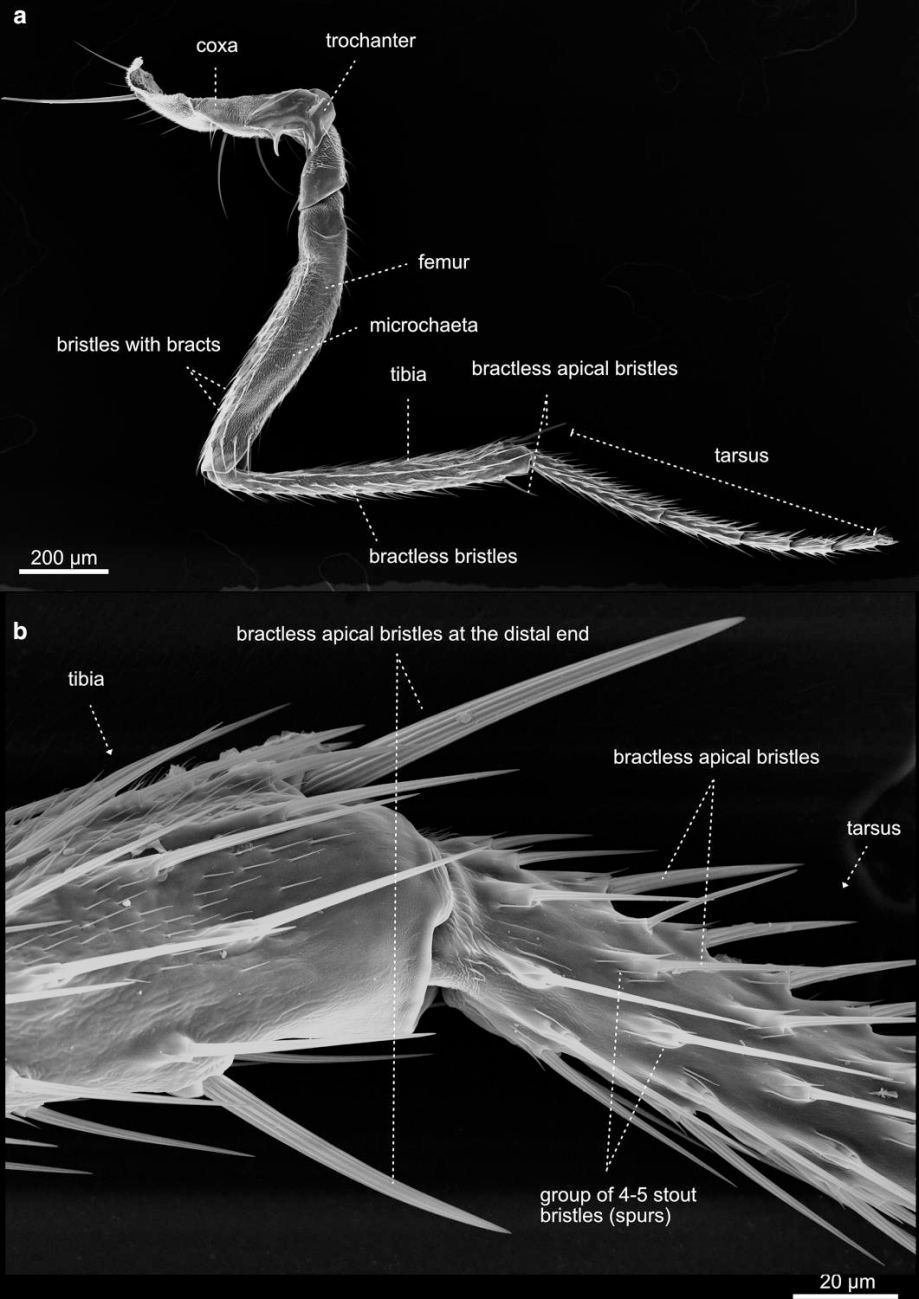
Legs—T2. The midleg of female *Drosophila*. The three legs of *D. melanogaster* differ in chaetotaxy, size, and structure. In contrast to the first pair of legs, the second a) and third pairs (Fig. 40) of legs do not show any signs of sexual dimorphism. b) In contrast to the bristles on the thorax, which can be divided easily into 2 classes, the macrochaetes, and microchaetes, the bristles on the legs vary greatly in size and show considerable morphological diversification.

**Fig. 42.**
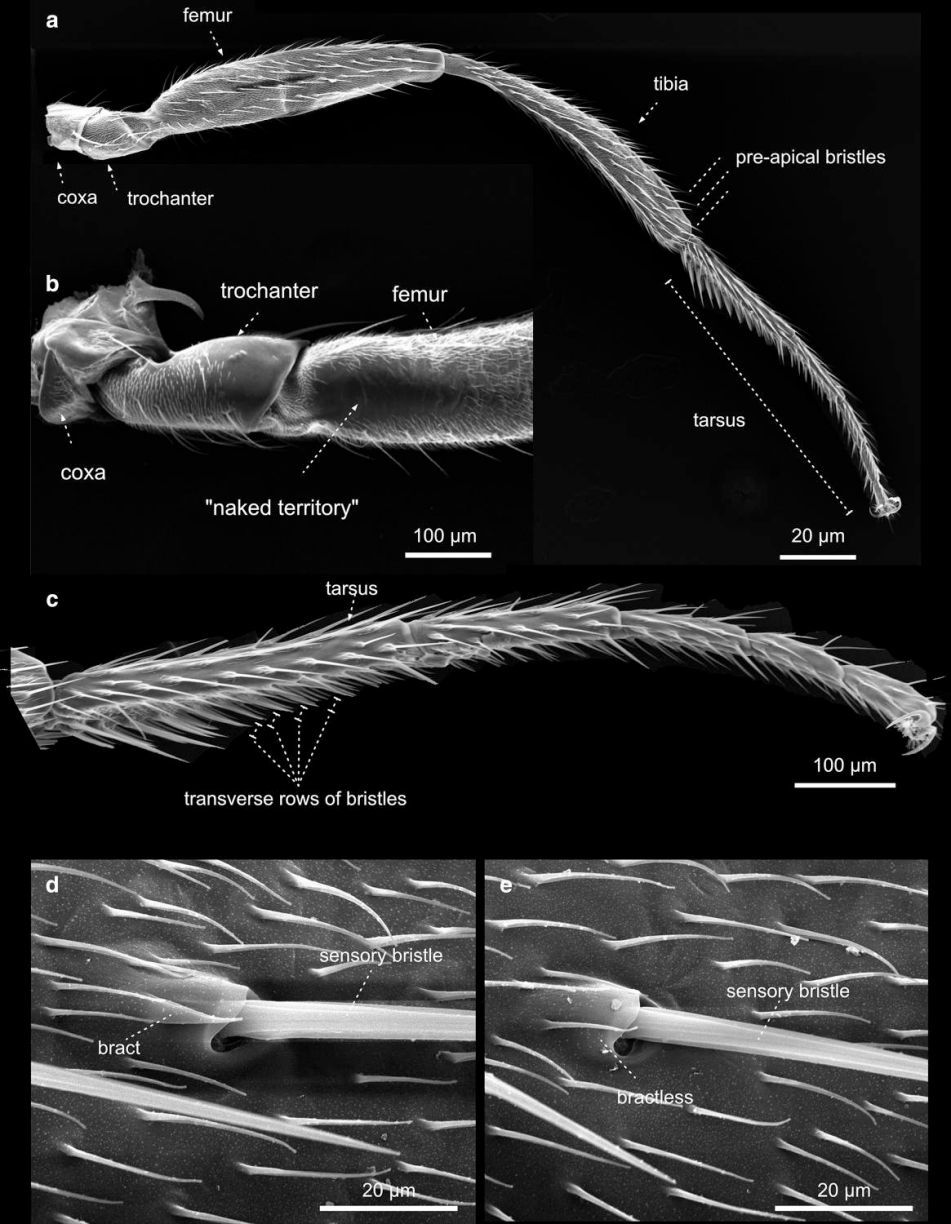
Legs—T3. a) The anterior aspect of a female hindleg, overview. b) The femur shows a bristleless “naked territory”, which varies in size between *Drosophilia* species. Posterior aspect. c) The tarsus of the hindleg shows characteristic arrangements of rows of small transverse bristles. d) and e) Enlargement of the sensory bristle d) with a bract or e) without.

**Fig. 43.**
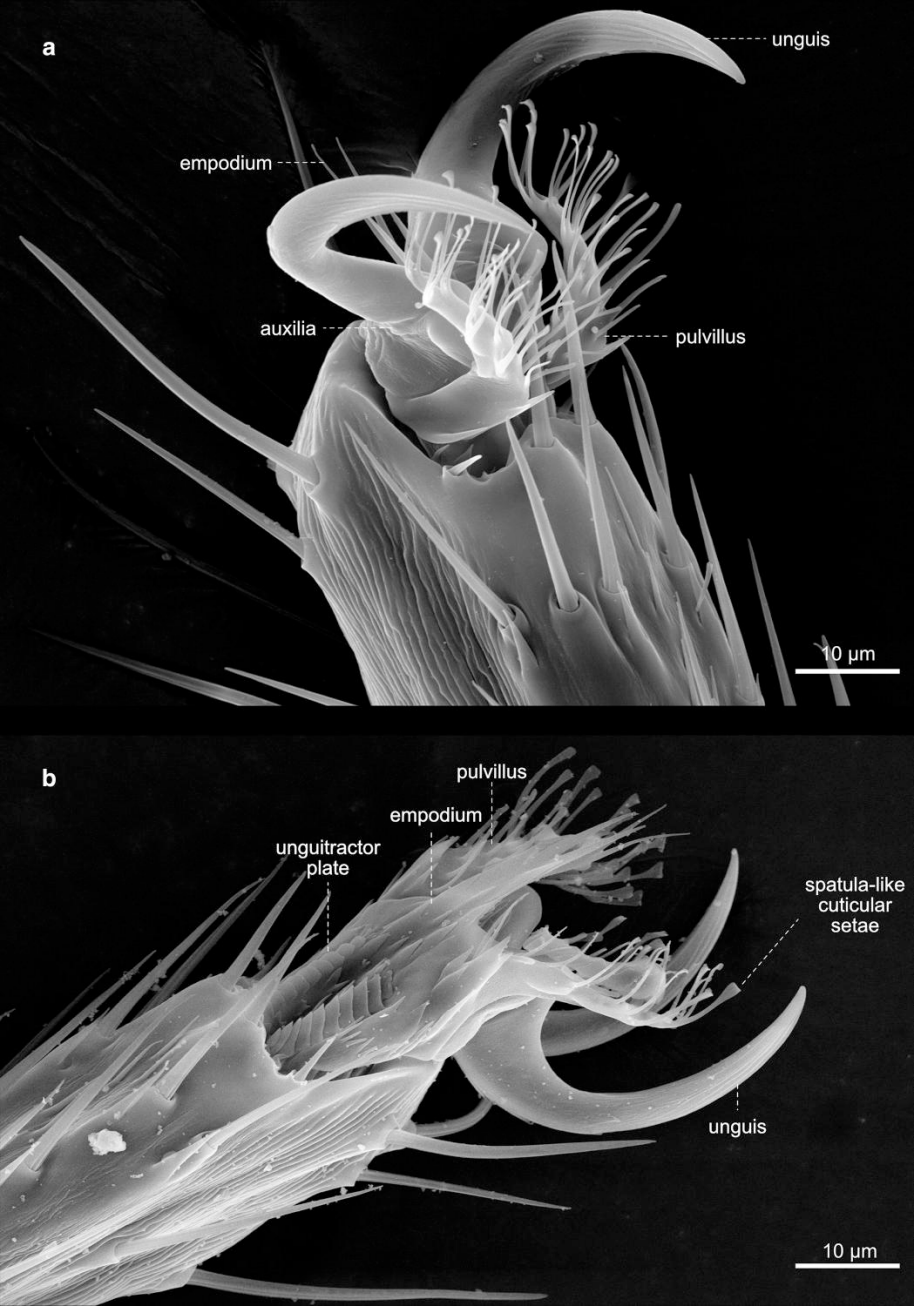
Feet a) in *Drosophila*, adhesion to smooth surfaces is mediated by “hairy” attachment devices, the pulvilli. Pulvilli are covered by hair-like cuticle outgrowths originating from epidermal cells. They possess spatula-like terminal contact zones. b) The claws, or ungues (sing. unguis), allow walking and attachment to rough surfaces. The structures shown are from T1.

The insect thorax (Figs. 29–43) bears the central organ systems, facilitating the locomotion of the animal—the wings (Figs. 35–37), the halteres (Figs. 38 and 39), and the legs (Figs. 40–43). Along the anterior-posterior axis, the thorax is, as for all insects, subdivided into the prothorax, mesothorax, and metathorax, which are formed de novo during metamorphosis but correspond to the larval thoracic segments T1–T3, respectively. Most of the visible thoracic anatomy is composed of the mesothorax, which is tremendously enlarged. At the same time, the anterior prothorax, as well as the posterior metathorax, are primarily reduced and formed by largely fuzed sclerites (Fabian *et al*. 2016). Along the dorsal–ventral axis, the fly's thorax subdivides into the pronotum, metanotum, and the ventral sternum (Figs. 30 and 31). If viewed from a dorsal perspective, the notum further subdivides into the scutum and the scutellum (Fig. 29). The pronotum is visible only when the head is removed and the thorax images from anterior (Fig. 30). The wings attach to the mesothoracic notum (T2) via cuticular hinges (Figs. 31 and 37). The sternum of each thoracic segment connects to one pair of legs. In the metathorax, halteres attach to the thoracic cuticle instead of wings (Fig. 38).

The thorax harbors 2 pairs of specialized openings to the tracheal system, the spiracles. They are located in the meso- and metathoracic segments (Figs. 31–33). The spiracles constitute large openings in the cuticle covered by a dense rim of cuticular bristles that hinder the inflow of dust particles and parasites. The mesothoracic spiracle (also referred to as *sp*1) exhibits a more oval shape. In contrast, the metathoracic spiracle (*sp*2), located just below the connection point to the haltere, appears nearly round (Figs. 32 and 33). Of note, in some textbooks, including the Demerec (Demerec 1950), the meso- and metathoracic spiracles are named *sp*2 and *sp*3. *Sp*1 is missing because the prothorax carries no spiracles due to its significant reduction. However, the thoracic spiracles are arranged to allow an efficient gas exchange for the flight musculature of the fly. Due to the increased metabolic load of the flight muscles during flight, especially the oxygen supply is of great importance. To regulate gas exchange and control the loss of water vapor, the opening area of the spiracles can be controlled and thus can vary in size and form when imaged under the microscope. For a more detailed description of spiracles and the regulation of gas exchange, we refer to the studies by Lehmann (2001) and Lehmann and Schützner (2010).

###  

#### Wings

Insect wings developed as protrusions from the second and third thoracic segments during evolution (Figs. 29–31 and 34–37). In *Drosophila*, as in all Diptera, the rear pair of wings was reduced to halteres (see next paragraph). Wings were one of the most innovative evolutionary novelties in insects. They enabled insects to conquer the air and to rapidly colonize new habitats. Wings were also highly beneficial in escaping predators. Thus, it is to be understood that more than 99% of all insect species belong to wing-bearing Pterygota. Insect wings have numerous sensilla identifiable on the leading edge (Figs. 34 and 35). These mechanoreceptors respond to airflow over the wings during flight, improving the ability to navigate during flight. Additional chemo- and mechanoreceptors on the wing react to external mechanical stimuli such as touch. Fully developed wings are thin, rigid flaps that arise during metamorphosis from epidermal outfolding (Alexander 2018). Hemolymph streaming, coordinated by wing hearts, is essential for wing unfolding and hardening (Tögel *et al*. 2008, 2013, 2022). The wing veins, arranged in a species-specific pattern, carry nerves and tracheal branches, continuously supporting such wings with hemolymph flow (Figure 36). The wing's anatomy is highly adapted to optimize the wing for aerodynamic, elastic, and inertial forces during active flight. For example, flies exhibit exceptionally high flight performance, with the highest agility and maneuverability.

#### Wing venation

The mechanical stability of the wings depends on the intactness of the main veins, which run along the wings’ length (Fig. 36). Cross-veins connect the central veins. The wing venation is highly invariable and has been extensively utilized to identify gene mutations responsible for cell polarity, wing differentiation, and other cellular processes. The original naming of the individual veins is based on the Comstock–Needham system, which was introduced over 100 years ago (Comstock and Needham 1898). The main longitudinal veins are named costa (C, meaning rib, the leading edge of the wing), subcosta (Sc, meaning below the rib, second longitudinal vein), radius (*R*, analogue to a bone in the forearm, third longitudinal vein), media (M, meaning middle, fourth longitudinal vein), cubitus (Cu, meaning elbow fifth longitudinal vein) and anal veins (A, meaning in posterior location, unbranched veins behind the cubitus). However, it should be mentioned that the terminology is still varying in the literature. For a more detailed description of wing venation, we refer to the studies by Snodgrass (1935), Ferris (1950), Triplehorn *et al*. (2005), and Chapman *et al*. (2013). For a more detailed description of the sensilla of the wings, we refer to the study by Cole and Palka (1982).

#### Wing hinge

Wings are connected to the thorax by flexible, membranous joins (Fig. 37). Within the membranous structures, the so-called axillary sclerites are embedded. They allow the transmission of muscular forces into the movement of wings for flight. Axillary sclerites also control the alula, a hinged flap at the base of the wing that, in some insects, accounts for up to 10% of the total wing area. The alula enables the wings to be folded back over the thorax and also contributes to the movement of the wing. They are, therefore, involved in flight-performance, for example, in terms of maneuverability (Walker *et al*. 2012). For additional details on the articulation of insect wings with the thorax, we refer to the studies by Demerec (1950), Bryant (1975), and Chapman *et al*. (2013).

#### Halteres

The halteres (Figs. 38 and 39) are susceptible sensory organs that measure the speed of rotational movements of the body during flight, based on the Coriolis forces. They also detect small deformations of the cuticle that occur during flight to gather more information. The halteres help to stabilize the flight by acting back on the motoneurons of the flight musculature. Halteres consist of a basal lobe (scabellum), a stalk (pedicellus), and a larger end-knob (capitellum). They carry a series of sensory sensilla, including groups of campaniform sensilla at the base, likely to be homologous with sensilla at the wing base. On the dorsal side of the halteres, two large groups of sensilla, organized into the basal and scapal plates, are present. Additional sensilla, known as Hicks papillae, are found near the basal plate, although they are reduced in number in Drosophilids and challenging to see without dissection, thus not shown in this atlas. For a comparison of haltere morphology and sensilla arrangement across Diptera, we refer to the study by Agrawal *et al*. (2017). On the ventral surface of the halteres, an additional scapal plate with sensilla and several Hicks papillae are located (not shown). Mechanosensory proprioceptive organs, so-called chordotonal organs, are also present and act in sensing stretch-induced forces acting at the basis of the halteres during flight. For a more detailed description of the sensilla of the halteres, we refer to the study by Cole and Palka (1982).

#### Legs

The legs connect to the body at the sternum of the thorax and are the crucial organ system allowing insects to walk and adhere to surfaces (Figs. 40–43). Each leg corresponds to a specific thoracic segment. Thus, the legs are annotated according to the segment they are attached to as prothoracic [T1 (Fig. 40)], mesothoracic [T2 (Fig. 41)], and metathoracic [T3 (Fig. 42)] legs. Despite an overall similarity, there are specific differences in the posture of each leg, the size of different leg segments, and the arrangement of bristles. The prothoracic leg (foreleg) usually bends toward the anterior to allow cleaning of the eyes and head appendages. The mesothoracic leg (midleg) has no apparent bias in its orientation, while the metathoracic leg (hindleg) usually bends posteriorly to allow cleaning of the wings and abdominal structures.

Along the proximal (closest to the body) to distal (furthest away from the body) axis, each leg consists of a coxa, a trochanter, a femur, a tibia, and a quinquepartite tarsus, and comprises a distal claw (Fig. 43). The most proximal segment of the tarsus is specifically referred to as the metatarsus. Generally, the prothoracic leg harbors the longest coxa. Compared with the coxa of the mid- and hindleg, which appear club-shaped, the coxa of the foreleg exhibits an apparent elongation along the proximal-distal axis. The femurs of the three legs all display a similar length. The tibia, on the other hand, is shorter at the forelegs than the other two.

The legs harbor hundreds of mechanosensitive bristles and specific gustatory sensilla. The number and patterning of bristles are species-specific. While *D. melanogaster* shows a small “naked territory” on the femur of the third thoracic (T3) leg (Fig. 42), *Drosophila virilis* has no such naked region. It has been postulated, for example, that mutations in the regulatory regions of the homeotic gene Ubx drive such species-specific novelties during evolution (Carroll *et al*. 2005). We cannot go into all the details of the arrangement and variation of the bristles on the legs here. For a detailed description of the chaetotaxy of the fly leg, we refer to the study by Hannah-Alava (1958).

In *Drosophila*, similar to other dipterans, adhesion to smooth surfaces is enabled by specialized attachment structures, the pulvilli, located on the tarsus of each leg (Fig. 43) (West 1862; Walker *et al*. 1985; Gorb 1998; Hüsken *et al*. 2015). Pulvilli harbor a number of cuticle outgrowth with a spatula-like terminal contact zone, which mediated adhesion to the smooth substrate.

## The abdomen with terminalia (analia and genitalia)

### Tergites and sclerites

**Fig. 44.**
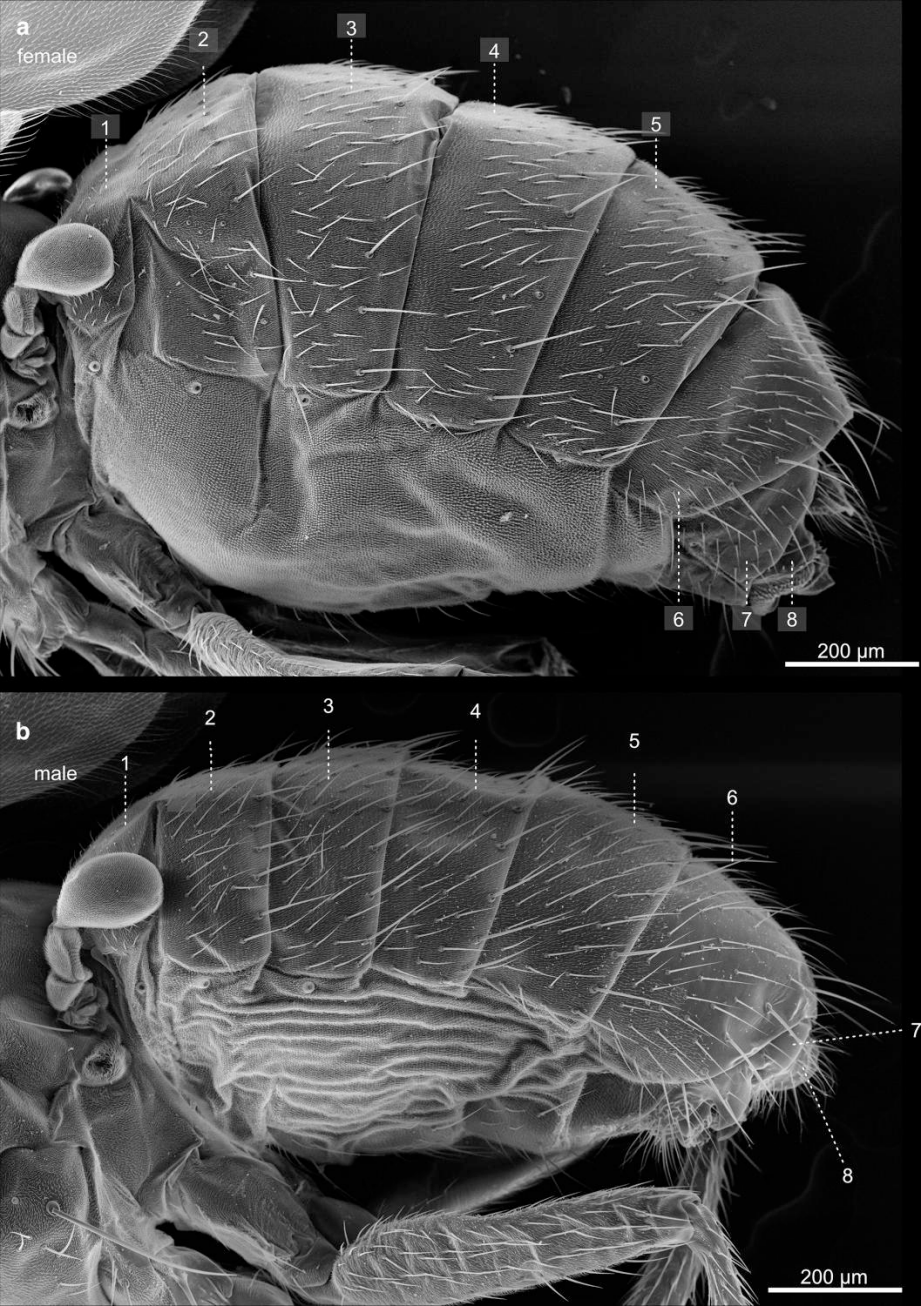
Abdomen—lateral view—female—male. Lateral view of the abdomen of a female a) and male b). Due to secondary modification, *Drosophila* shows an altered segmental pattern in both sexes compared with the ground pattern. Seven pairs of abdominal spiracles, the opening of the tracheal system, are built; some are visible in the upper image. The dorsal part of the abdomen carries the tergites, and the ventral part, the sternites, is visible between a flexible membranous epidermis. This soft area of the body may show wrinkles or folds. We speculate that this may depend on the amount of liquid or material (well-developed ovaries) in the abdominal cavity. The abdominal segments house vital internal organs, including the digestive system, reproductive organs, and part of the respiratory system.

**Fig. 45.**
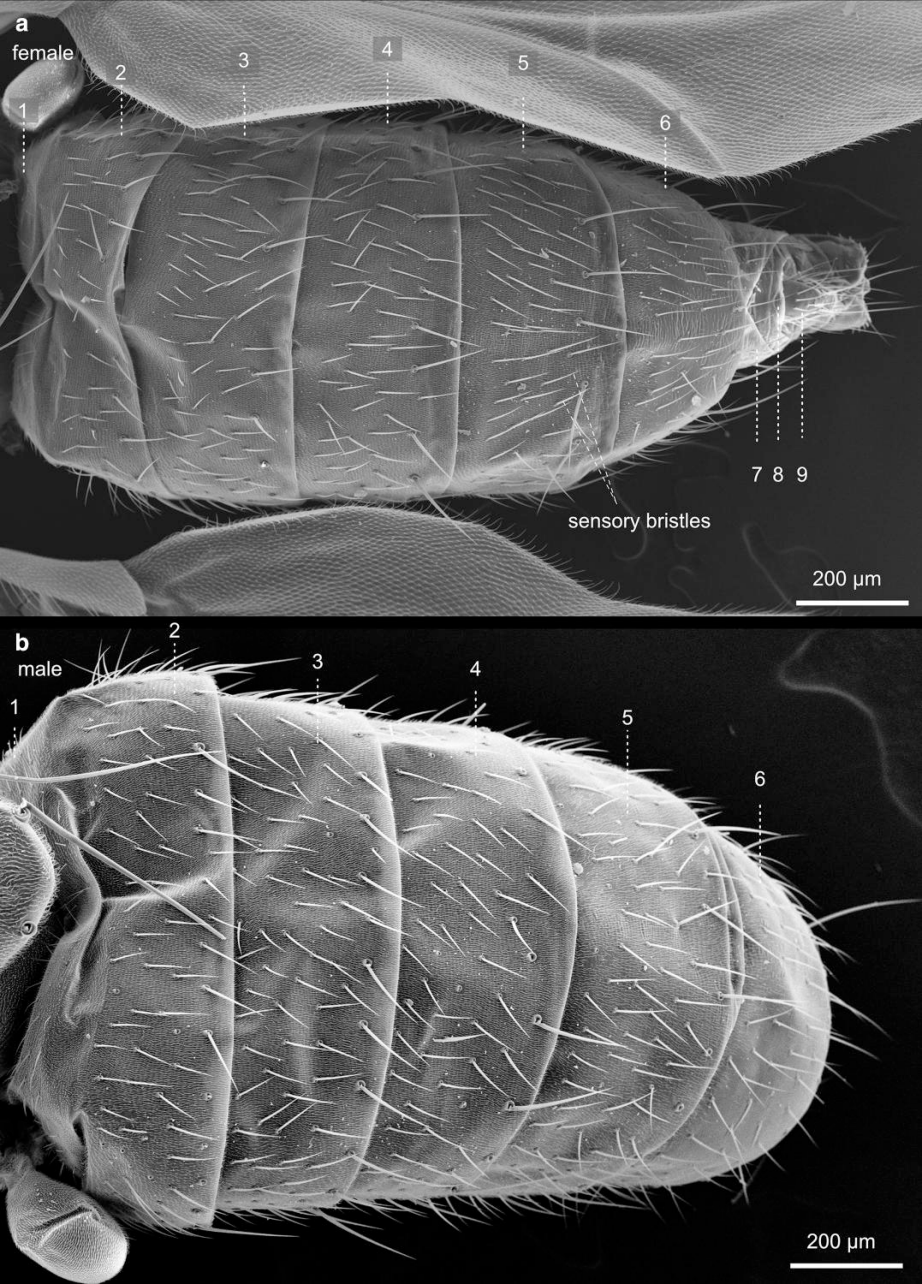
Abdomen—dorsal view—female—male—tergites. Close-ups of the tergites of a female a) and a male b). The abdomen's cuticle displays intricate patterns and textures unique to each species and can aid in identifying different *Drosophila* species. Tergites are interconnected by a flexible, membraneous tissue to allow flexibility and movement, enabling the fly to perform various behaviors, such as grooming, stretching, and bending. Moreover, these structures provide stability and support during flight, walking, feeding, and reproduction.

**Fig. 46.**
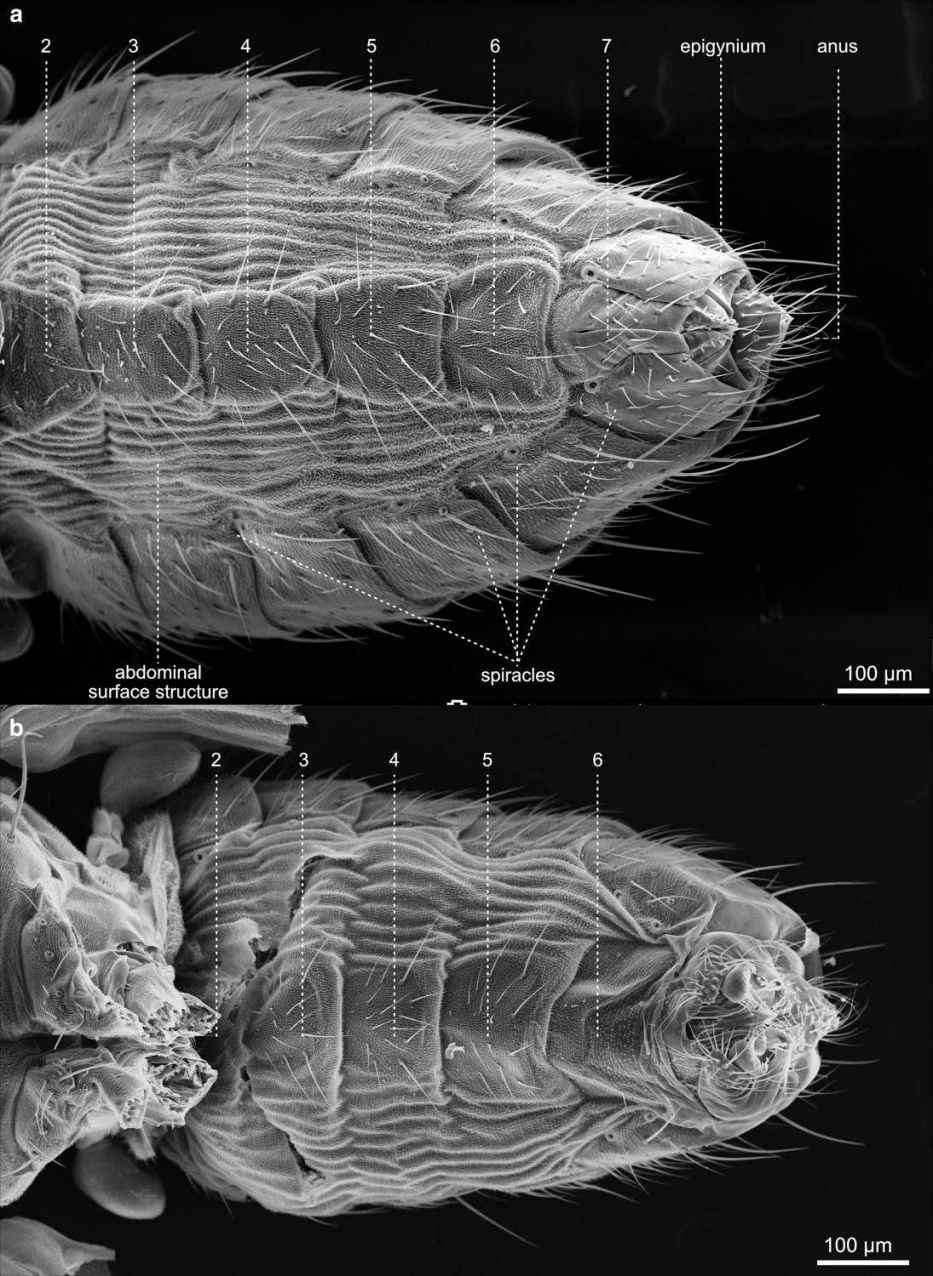
Abdomen—ventral view—female—male—sternites. Close-ups of the ventral abdominal surface structures of a) a female and b) a male. The sternites are, like the tergites, interconnected by flexible tissue. *Drosophila* typically has eight abdominal sternites, each corresponding to one of its abdominal segments. The surface structure has various uses in *Drosophila.* It is specialized to accommodate sensory functions, reproduction, communication, and other behaviors contributing to the fly's survival and successful reproduction. The abdominal sternites are pivotal in coordinating the fly's motor functions, allowing for precise control of body movements, posture, and interactions with the environment.

**Fig. 47.**
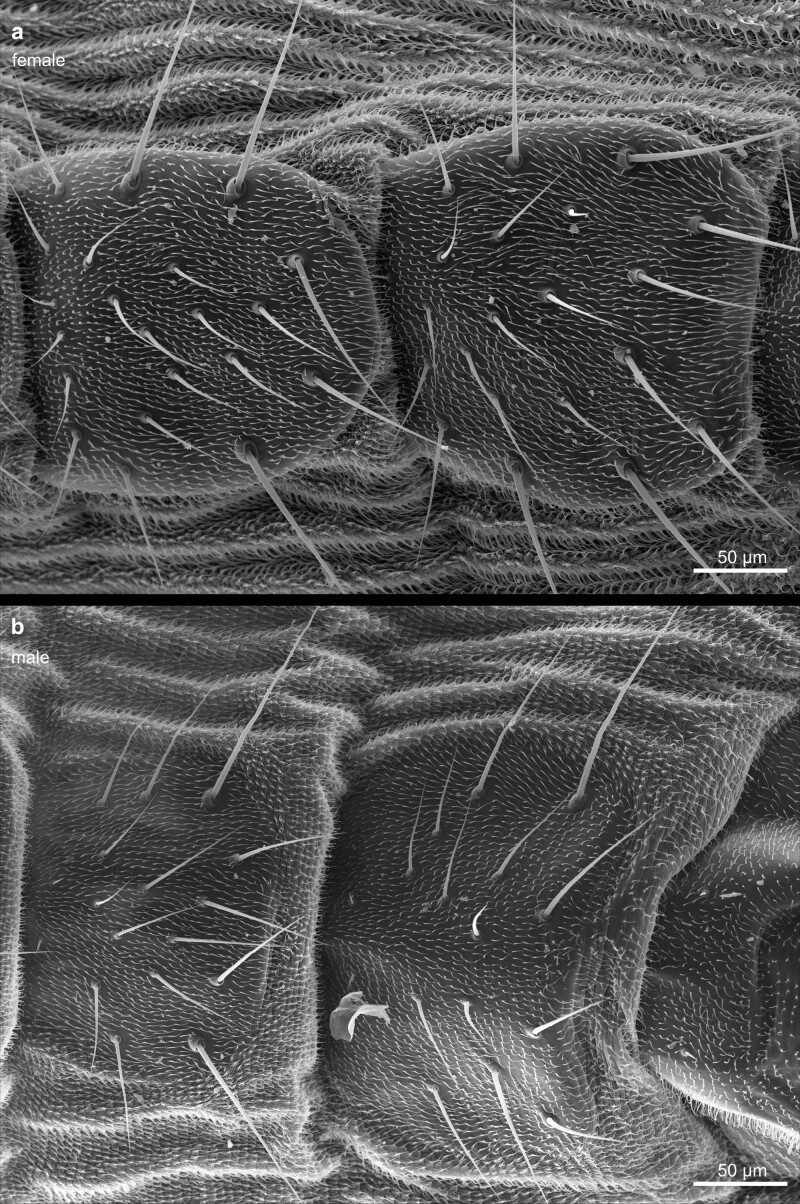
Abdomen—sternites. Close-ups of a) a female and b) a male sternites. These sternites provide attachment points for muscles and play a crucial role in the fly's movement, stability, and overall functionality.

Compared with the total length of the fly, the abdomen constitutes the largest of the three main body parts. It contains the central organ systems, including parts of the digestive tract, the gonads, the fat body, part of the heart, and the Malpighian tubules. The most prominent feature of the outer morphology of the abdomen is its metameric arrangement of cuticular segments along the anterior-posterior axis (Fig. 44). The dorsal half of the abdomen is built by sclerotized cuticle plates—the tergites (Fig. 45). Each tergite is separated from the next by a thin, flexible sheet of membranous cuticle, which accounts for high flexibility, allowing the animal to bend the abdomen extensively. On the lateroventral half of the abdomen, we found the sclerites, also sclerotized cuticular plates (Figs. 46 and 47). The ventral part of the sclerites, which represent the bottom of the abdomen, carry several large bristles that may serve to sense the ground surface. Many arthropods, including insects, harbor pleurites, which are, like tergites and sclerites, chitinous plates at the lateral wall of the insect body. They support wings and the legs structurally, as well as the lateral wall of the abdomen. From an evolutionary point of view, pleurites may have evolved from subcoxal leg segments (Coulcher *et al*. 2015). However, abdominal pleurites are not present in *Drosophila* or are largely reduced or fuzed with other cuticular structures, as happened in the thorax.

### Terminalia

**Fig. 48.**
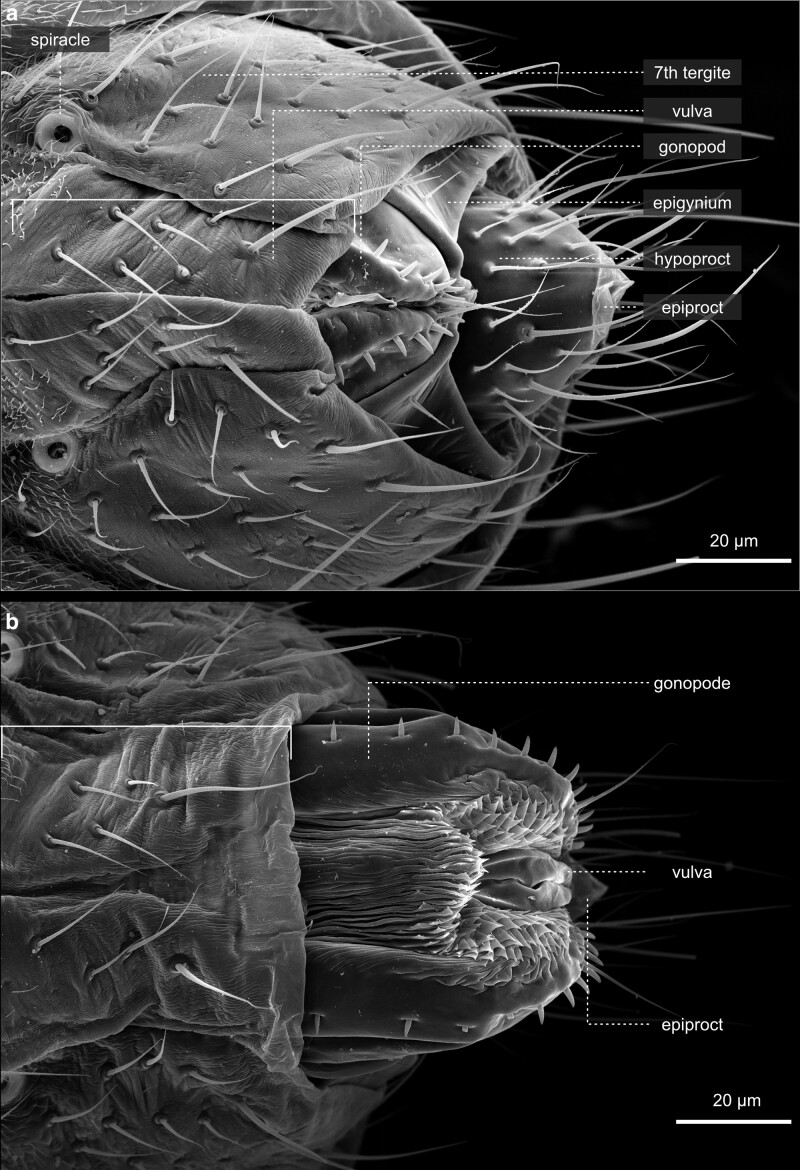
Terminalia—female—overview. The terminalia of the female fly are specialized structures involved in egg-laying, sperm storage, and reproductive adaptations. The egg-laying apparatus can be retracted a) or bulged out b). The terminalia showcase the fascinating complexity of reproductive anatomy in these insects and are directly integrated into the tergite structures. The female genital opening is between the eighth and ninth abdominal segments.

**Fig. 49.**
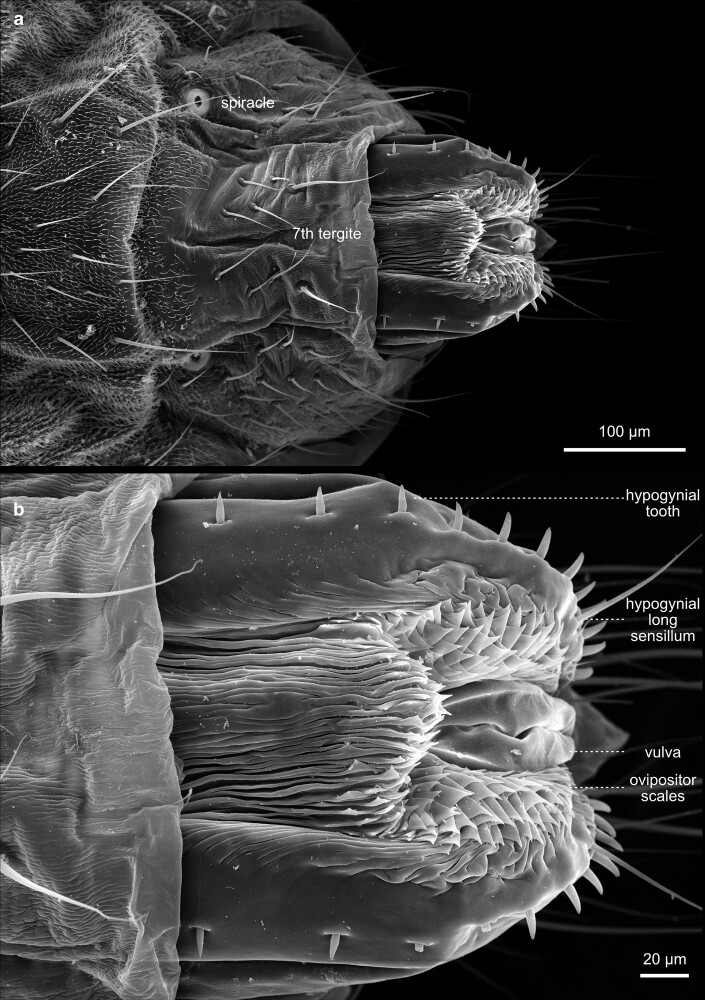
Terminalia—female—details. a) Lateral view of the posterior genital region of a female. Female flies possess a movable oviposition apparatus, the ovipositor, which attaches the eggs to a suitable substrate. b) Close-up of the ovipositor.

**Fig. 50.**
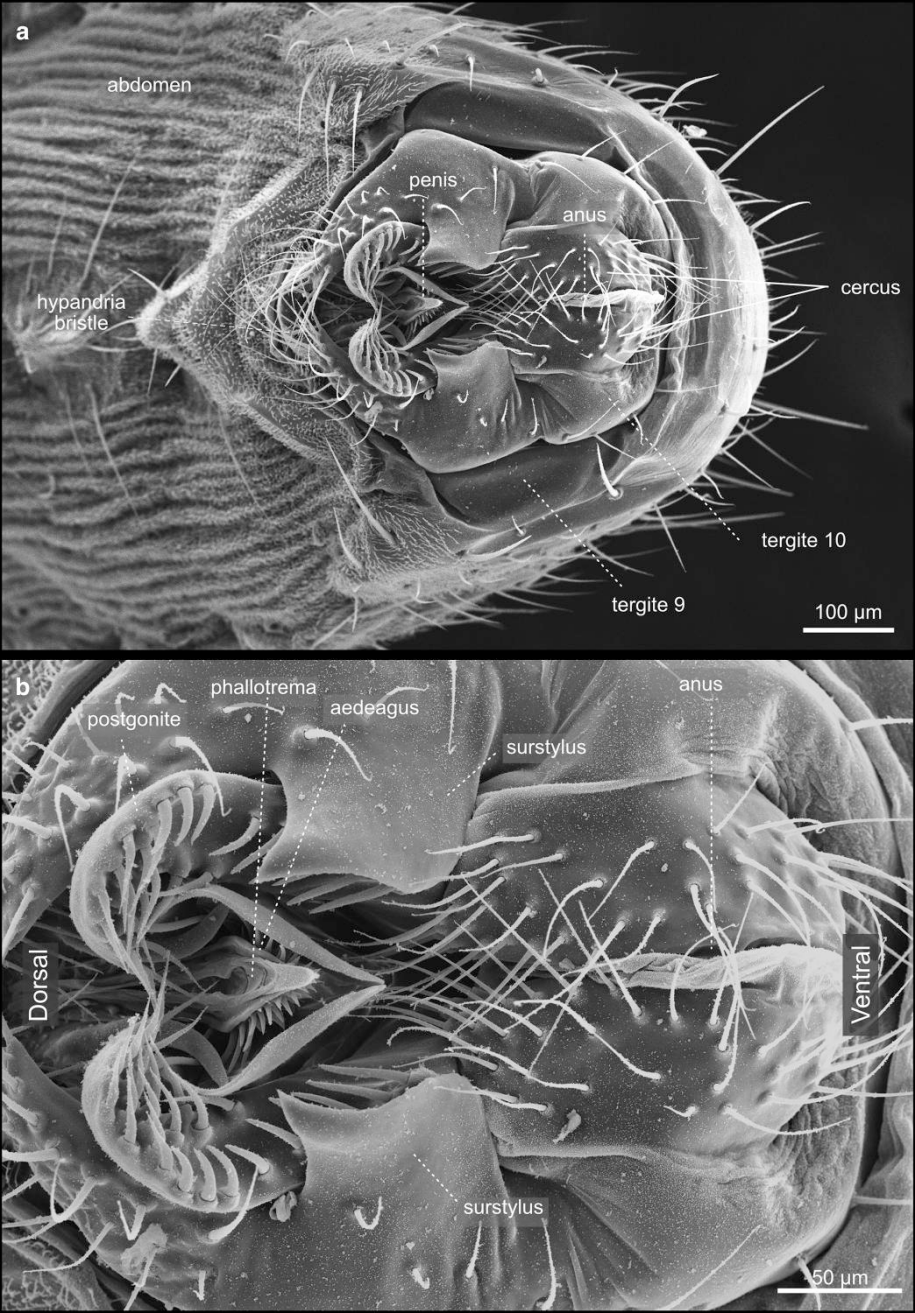
Terminalia—male—overview. a) Caudal view. The terminalia of the male holds the structures required for the formation of a spermatophore and sperm transmission to the female. The genital opening of the male is located between the ninth and tenth segments. b) Close-up of the male genital structures.

**Fig. 51.**
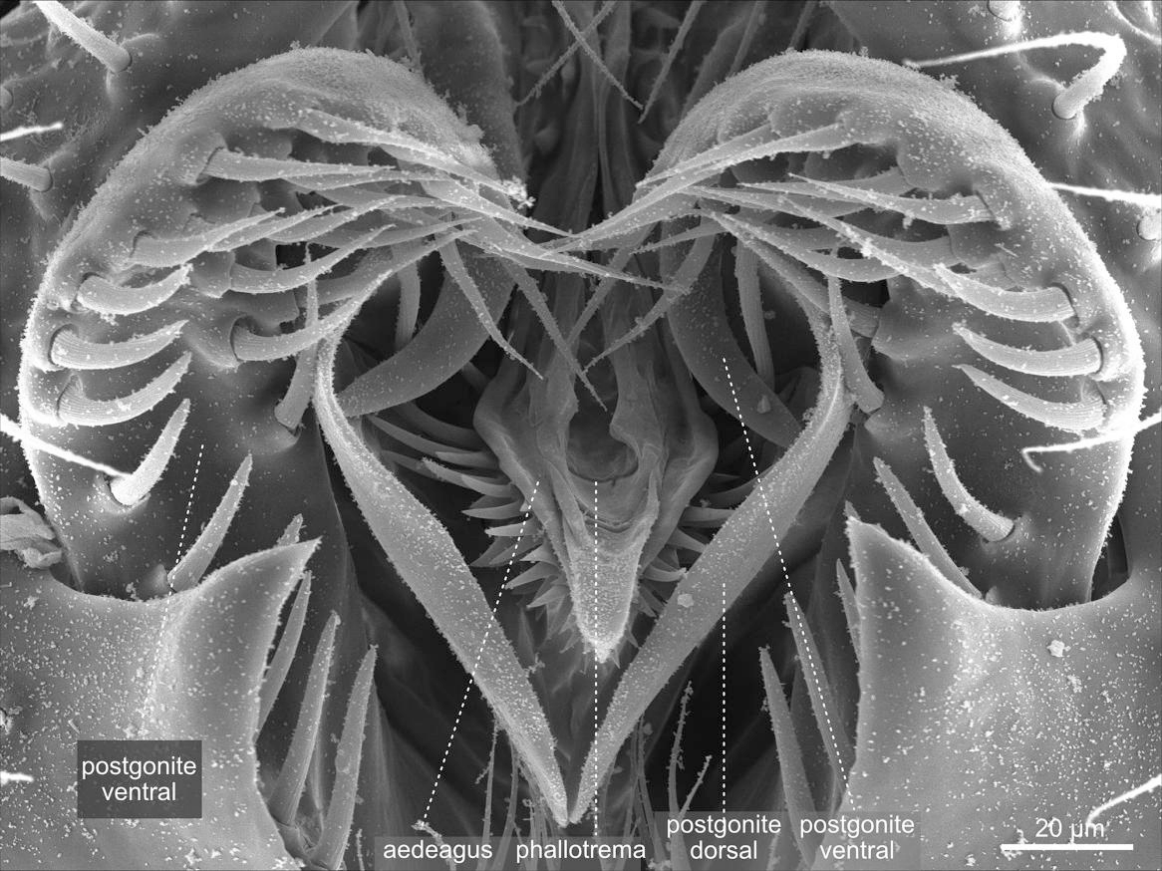
Terminalia—male—phallic structures: Caudal view of the penis and associated structures.

**Fig. 52.**
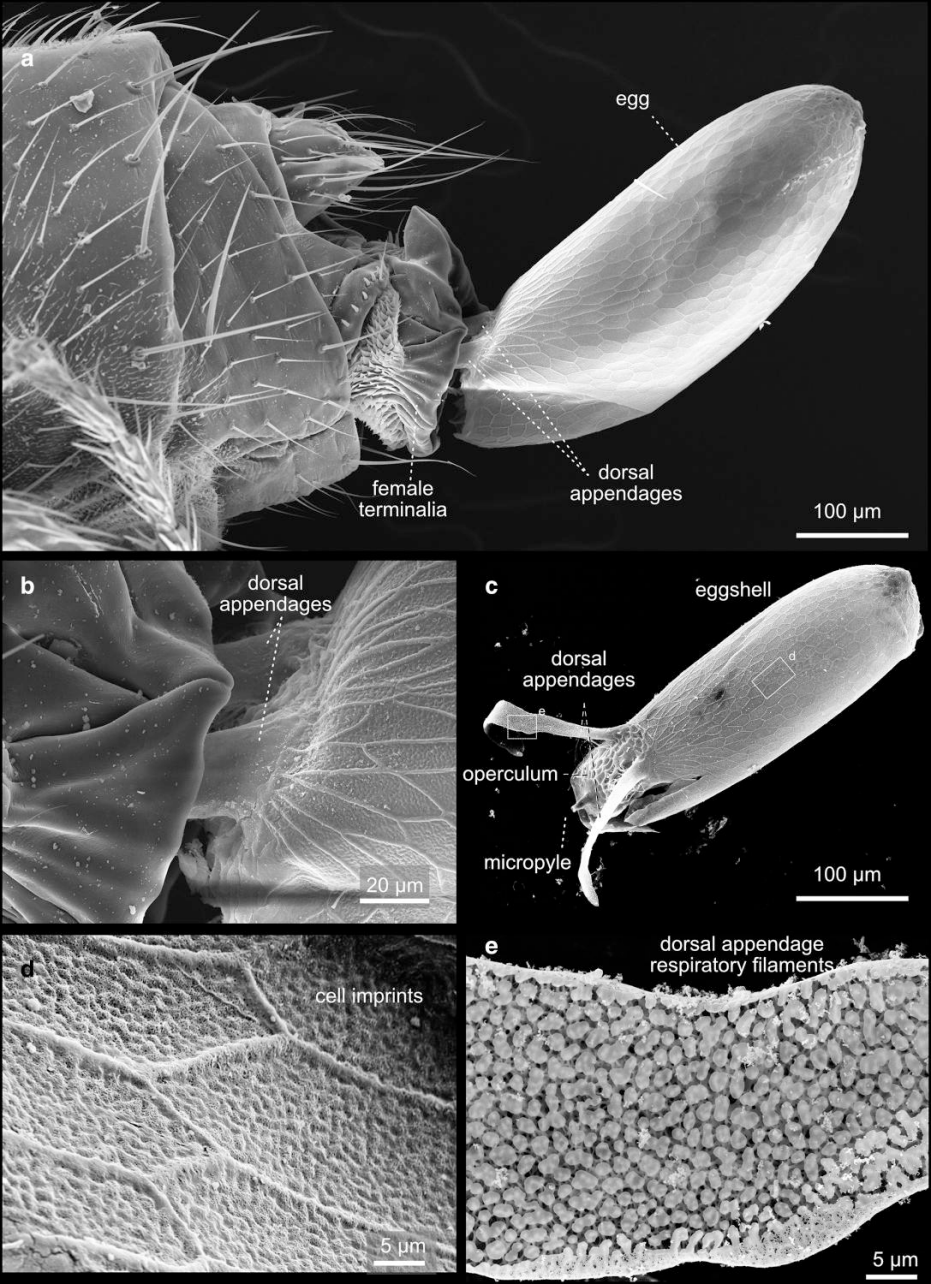
Terminalia—female—eggs: a) Lateral view of a female laying an egg. b) Close-up of the opening of the ovipositor and the released egg, lateral view. The respiratory filaments are still inside the female’s genital tract. c) Overview of an eggshell with an egg. d) Close-up of the surface of an eggshell. The remains of the imprints of the follicle cells are visible. e) Close-up of the dorsal appendages (respiratory filaments).

As with all insects, terminalia are the terminal body structures of the abdomen that build the external genitalia (Figs. 48–52). External visible genital structures arise from the genital disc and correspond to segments 8–10.

During metamorphosis, the genital disc develops into 3 major primordia, which are different in females and males. The first primordium, which corresponds to a reduced abdominal segment 8, develops in females into most of the visible genital structures. In males, this part is highly reduced and gives rise to a tiny eighth tergite called epandrial anterodorsal phragma in recent literature (Keisman *et al*. 2001; Rice *et al*. 2019). The second primordia that arises from the genital disc represents the abdominal segment 9, which builds the visible external male genitalia. Finally, the third primordium represents abdominal segment 10 and forms the analia in females and males (Keisman *et al*. 2001; Rice *et al*. 2019). During development, the *D. melanogaster* male genitalia rotate 360 degrees clockwise, causing the internal organs to loop around the gut; this rotation and, thus, the dorsal–ventral designation of the genitalia varies within Diptera (Suzanne *et al*. 2010).

Recent work by Rice and colleagues to unify the nomenclature used to describe male terminalia in *Drosophila* suggested subdividing the terminal structures into two parts: periphallic structures and phallic structures. The latter once comprises all intromittent organ aspects and structures that are connected to it (Rice *et al*. 2019). Periphallic structures include the cercus (former anal plate), the epandrium (former genital arch), the pair of surstyli (former claspers), and the subepandrial sclerite (former pons) that connects the surstyli to other periphallic structures (Rice *et al*. 2019). Periphallic structures support sperm transfer by, e.g. supporting phallic parts, but are not directly involved in sperm transmission. In females, the ovipositor structures are required for egg-laying (Fig. 2). The egg leaves the mother with its posterior end first.
